# Global, regional, and national time trends in the burden of epilepsy, 1990–2019: an age-period-cohort analysis for the global burden of disease 2019 study

**DOI:** 10.3389/fneur.2024.1418926

**Published:** 2024-08-15

**Authors:** Tao Shan, Yahui Zhu, Haozhi Fan, Zeye Liu, Jing Xie, Mao Li, Shenqi Jing

**Affiliations:** ^1^Department of Outpatient, The First Affiliated Hospital with Nanjing Medical University, Nanjing, China; ^2^School of Information Management, Nanjing University, Nanjing, China; ^3^Department of Medical Informatics, School of Biomedical Engineering and Informatics, Nanjing Medical University, Nanjing, China; ^4^Institute of Medical Informatics and Management, Nanjing Medical University, Nanjing, China; ^5^Jiangsu Province Engineering Research Center for Chronic Disease Big Data Application and Intelligent Health Service, Nanjing, China; ^6^Department of Neurology, Beijing Tiantan Hospital, Capital Medical University, Beijing, China; ^7^Jiangsu Institute of Clinical Medicine, The First Affiliated Hospital with Nanjing Medical University, Nanjing, China; ^8^School of Cyber Science and Engineering, Southeast University, Nanjing, China; ^9^Department of Cardiac Surgery, Peking University People’s Hospital, Peking University, Beijing, China; ^10^Department of Pharmacy, Zhongda Hospital, School of Medicine, Southeast University, Nanjing, China; ^11^Department of Neurology, the First Medical Center, Chinese PLA General Hospital, Beijing, China; ^12^Center for Data Management, The First Affiliated Hospital with Nanjing Medical University, Nanjing, China; ^13^Department of Information Management, The First Affiliated Hospital with Nanjing Medical University, Nanjing, China

**Keywords:** epilepsy, prevalence, disease burden, years lived with disability, age-period-cohort

## Abstract

**Background:**

Epilepsy is a non-communicable chronic brain disease that affects all age groups. There are approximately 50 million epilepsy patients worldwide, which is one of the most common neurological disorder. This study reports the time trends in the burden of epilepsy from 1999 to 2019.

**Methods:**

We evaluated the disease burden and its temporal trends of epilepsy using the prevalence and years lived with disability (YLDs), which was estimated based on the Global Burden of Disease (GBD) 2019 study. The age-period-cohort (APC) model was used to estimate the temporal trends of the epilepsy prevalence and YLDs rates, and to analyze the relative risks of age, periods and queues (age/period/queue effects).

**Results:**

In the past 30 years, the global age-standardized prevalence rate and age-standardized rate has increased by 29.61% and 27.02%, respectively. Globally, the APC model estimated the net drift of prevalence and YLDs were 0.88% (95% CI: 0.83–0.93) and 0.80% (95% CI: 0.75–0.85) per year. Among 204 countries and territories, the YLDs in 146 and prevalence 164 showed an increasing trend. And the risk of YLDs and prevalence increases with age, with the lowest risk among 0–4 years old and the highest risk among 75–79 years old. Unfavorable increasing period and cohort risks of YLDs and prevalence were observed.

**Conclusion:**

Over the past 30 years, the YLDs and prevalence of epilepsy have gradually increased globally and unfavorable increasing period and cohort risks were observed. Emphasizing epilepsy prevention, strengthening epilepsy health education, optimizing older adults epilepsy diagnosis and treatment plans, and actively promoting epilepsy diagnosis and treatment plans can effectively reduce new cases of epilepsy and related disabilities.

## 1 Introduction

Epilepsy is a chronic non-communicable brain disease that affects populations around the world. There are approximately 50 million epilepsy patients worldwide, which is one of the most common neurological disorders worldwide. Its typical symptoms are tonic clonic seizures, strong clonic seizures, dystonia, irritable seizures, and focal sudden weakness (1). Although some neurological and systemic conditions are important causes of epilepsy, about 50% of epilepsy cases worldwide have unknown causes (2). In many countries and territories, epilepsy is unacceptable and stigmatized. Due to the social, economic, and cultural limitations, the etiology discovery and diagnosis of epilepsy may be difficult to make in some low-income countries (3). Different understandings of epilepsy in society, economy and culture, and some environmental risk factors, have led to differences in the prevalence, course, and treatment effectiveness of the disease worldwide (4). The recurrent seizures and the physiological and psychological effects they have on patients make epilepsy one of the heavy burdens on the nervous system. However, up to 70% of people with epilepsy can be effectively controlled with appropriate treatment, thus avoiding seizures (5, 6).

The Global Burden of Disease (GBD) publishes disease data for the world and various regions/countries, and is a comprehensive indicator for measuring the impact of various diseases on the health status of the global population. GBD 2015 found that the global age-standardized prevalence of epilepsy ranked fifth among neurological diseases from 1990 to 2015, only behind stroke, migraine, dementia, and meningitis. However, age-standardized Disability Adjusted Life Years (DALYs) and epilepsy-attributable mortality significantly decreased, accounting for 5.0 and 1.3% of all neurological disease deaths, respectively (7, 8).

For patients with epilepsy, there are age-specific differences in the risk of epilepsy incidence, disability. In addition, due to the persistent impact of early diagnosis and treatment on the life outcomes of epilepsy patients, there may also be differences between birth cohorts due to the application of new diagnostic and treatment methods (cohort effect) (9). Over a period of time, technological advancements or changes in health policies related to epilepsy management can affect the prevalence, disability, and mortality risks of epilepsy patients during the same period (cyclical effect). Thus, when analyzing the prevalence and disease burden of epilepsy patients, it is important to pay attention to age, period, and cohort effects (9, 10). This current study used age-period-cohort (APC) models to estimate the temporal trends of the epilepsy prevalence and Years Lived with Disability (YLDs) rates in 204 countries and regions from 1990 to 2019, and to analyze the relative risks of age, periods and queues (age/period/queue effects).

## 2 Materials and methods

### 2.1 Data sources

GBD 2019 analyzed and estimated the disease burden of 369 diseases and injuries in 204 countries and territories using a unified and comparable method by collecting all available data from different ages, times, geography, health reasons and fields, and further systematically sorted out the attributed disease burden of 87 risk factors (11, 12). For countries and territories without primary data sources, GBD estimates the disease burden using a Bayesian framework. GBD displays the prevalence and YLDs values of all diseases, along with their 95% uncertainty interval (UI) (12). All data in this study were from GBD 2019, and detailed information on data input, processing, synthesis, and final model, please refer to the relevant requirements of GBD 2019 publication (12). Due to patient information desensitization in the GBD study, the Institutional Review Board of the University of Washington approved the waiver of informed consent.

To analyze the prevalence and disease burden of epilepsy with different social and demographic development status, this study used the Social Demographic Index (SDI) as an indicator. SDI is a comprehensive indicator that combines three components: Lag Distributed Income *per Capita* (LDI), Mean Education for Those Age 15 and Older (EDU15+), and Total Fertility Rate Under 25 (TFU25). The value ranges from 0 to 1 and can be classified as low SDI, low-middle SDI, middle SDI, high-middle SDI, and high SDI countries based on the SDI values of each country (12).

### 2.2 Definition of epilepsy and GBD study metrics

The patients included in this study included idiopathic epilepsy and secondary epilepsy, with ICD-9 diagnosis code of Code 345.00 and ICD-10 diagnosis codes of G40 and G41 (7, 13). The diagnostic criteria for epilepsy were based on the Epidemiological Study Guide for Epilepsy published by the International League Against Epilepsy (ILAE), including: (1) at least 2 non-induced or non-reflexive seizures with an interval of 24 h and consistent with epilepsy syndrome; (2) only 1 non-induced or non-reflexive seizure with a recurrence risk in the next 10 years equivalent to that of two non-induced seizures (at least >60%) (14). The YLDs rate range from 0 (completely healthy) to 1 (death), which was calculated by multiplying the number of cases in a specific period by the average duration of the disease, and then multiply by a weight coefficient (15).

### 2.3 Analysis of overall time trends

The disease burden of epilepsy was assessed using the overall age prevalence (crude prevalence) and overall age YLDs (crude YLDs), age-standardized prevalence and age-standardized YLDs, as well as the time trends of prevalence and YLDs. The age-standardized prevalence and age-standardized YLDs were calculated using the global age-standardized population data. The changes in prevalence and YLDs over time were assessed using the relative change in the percentage of prevalence rate and YLDs, and the time trend of the prevalence and YLDs was assessed using the joinpoint regression model. The annual percentage change was calculated and compared to 0 to investigate whether the fluctuation trend in different regions was statistically significant (16). The joint point regression model was developed by the National Cancer Institute of the United States to conduct detailed assessments of disease change characteristics at different time intervals. In addition, the population was divided into five age groups (1–19, 20–39, 40–59, 60–79, 80+) according to age, and the age distribution of prevalence and YLDs was calculated to estimate the proportions of prevalence and YLDs from each age group each year.

### 2.4 Age-period-cohort modeling analysis

The age-period-cohort (APC) model was used to analyze the potential trend of epilepsy prevalence and YLDs. APC model is a model commonly used to analyze the change trend and causes of incidence, prevalence, and mortality of chronic diseases. The classic APC model can describe the change trend of diseases according to the impact of age, period and cohort on incidence, prevalence or mortality, and predict according to the change trend. There is a complete linear relationship of “period = age + queue” among the three factors in the APC model in the APC model, making parameter estimation difficult. In this study, this issue can be avoided by developing estimable APC parameters and functions without imposing arbitrary restrictions on model parameters. We implemented the APC model using R tools, the details of which are described in previous literature (17–19).

This study used the estimated prevalence and YLDs values of epilepsy in GBD 2019, as well as population data, as input data for the APC model. Among them, GBD data for 16 age groups (5–9, 10–14, …, 80–84) were arranged into a single unit framework to represent characteristic ages, GBD data for six time points (1990–1994 [1992], 1995–1999 [1997], …, 2015–2019 [2017]) were arranged into a single unit framework to represent specific periods, and GBD data for 21 birth cohorts represented by birth years (1913–1917 [1915], 1918–1922 [1920], …, 2013–2017 [2015]) were arranged into a single unit framework to represent specific birth cohorts. A fitted APC model was used to estimate the net shift in epilepsy prevalence and epilepsy YLD, considering age, period, and cohort effects. To reflect the birth cohort and period effects, the APC model calculated the annual percentage change in age-specific prevalence and age-specific YLDs, and randomly selected group as the reference period (cohort) (17). Relative risk was used to calculate the age specific incidence rate ratio of each period (cohort) to the reference period (cohort). The Wald χ^2^ test was used to test the significance, and *p* values <0.05 suggested statistically significant. All analyses were conducted in R(4.3.0).

## 3 Results

### 3.1 Global and regional trends

**Figure 1:**
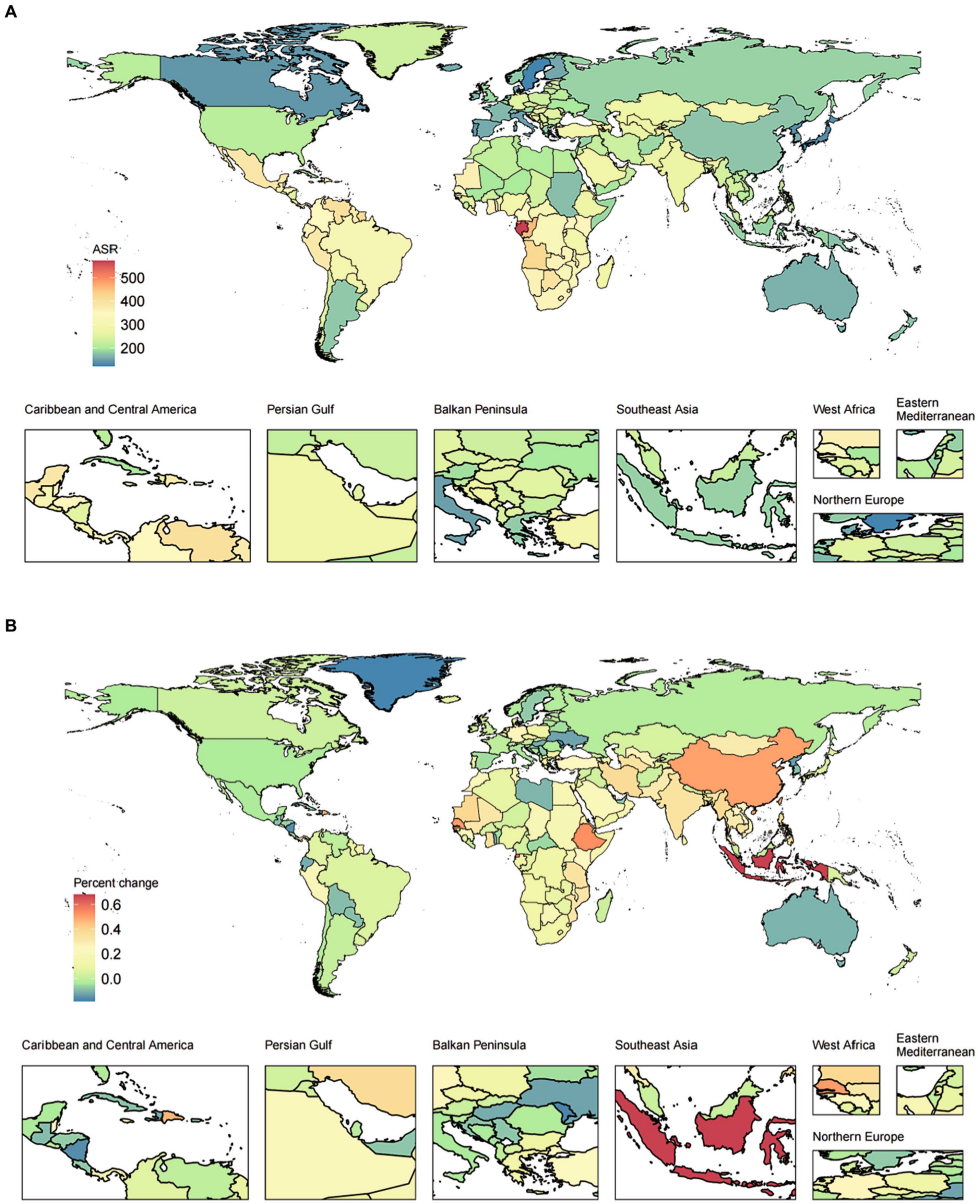
The ASR in 2019 **(A)** and percent change (%) of the ASR during 1990–2019 **(B)** for epilepsy YLDs. ASR, age-standardized rates; YLDs, years lived with disability.

Table 1 and Figure 1 show the global population and the total number of YLDs, all-age YLDs rate, age-standardized YLDs, and net YLD drift of epilepsy worldwide. From 1990 to 2019, the global population increased from 5.40 billion (95% UI: 5.20–5.50) to 7.70 billion (95% UI: 7.50–8.00), an increase of 44.60%, and the number of epilepsy YLDs increased from 10074.53 thousand (95% UI: 6604.44–14181.97 thousand) to 18305.39 thousand (95% UI: 12284.95–24795.74 thousand), an increase of 81.70% (62.43,104.91). Among them, the highest increase in the number of epilepsy YLDs was in low-SDI regions (166.14% [116.21,240.30]), while the lowest increase was in high-SDI regions (29.82% [13.74,46.95]). The all-age YLDs rate (25.63% [12.31–41.68]) and the age-standardized YLDs rate (27.02% [14.32–42.25]) for epilepsy had increased in each SDI regions worldwide. Among them, the highest increase in all-age YLDs rate was in low-middle-SDI regions (33.35% [10.21–65.49]), while the lowest increase was in high SDI regions (5.30% [−7.74–19.20]). The highest increase in age-standardized YLDs rate was in middle-SDI regions (33.42% [15.46,55.61]), while the lowest increase was in high SDI regions (04105973927% [−7.36,17.51]). In addition, the estimated net drift of YLDs for epilepsy through the APC model was 0.80% (95% CI: 0.75–0.85) per year, the highest increase was in low-middle-SDI regions (1.00% [0.95,1.05]), while the lowest increase was in high-SDI regions (0.46% [0.44,0.48]) and high-middle-SDI regions (0.46% [0.42,0.50]). Similar trends were also observed in the prevalence of epilepsy, as detailed in Supplementary Table S1 and Supplementary Figure S1. The results of YLDs and prevalence in male and female were detailed in Supplementary Tables S2, S3 and Supplementary Figures S2, S3.

**Table 1:** Trends in epilepsy YLDs for both genders across SDI quintiles, 1990–2019.

	Global		High SDI		High-middle SDI		Middle SDI		Low-middle SDI		Low SDI	
	1990	2019	1990	2019	1990	2019	1990	2019	1990	2019	1990	2019
**Population**												
Number, n × 1,000,000	5,350 (5,239,5,460)	7,737 (7,483,7,993)	822	1,013	1,150	1,430	1,717	2,397	1,130	1,764	528	1,128
Percentage of global, %	100	100	15.40	13.10	21.50	18.50	32.10	31.00	21.10	22.80	9.90	14.60
**YLDs**												
Number, n × 1,000	10074.53(6604.44,14181.97)	18305.39(12284.95,24795.74)	1503.70(964.08,2085.47)	1952.07(1254.20,2796.50)	2092.67(1379.60,2849.39)	2961.97(1953.53,4024.78)	3053.01(1977.86,4275.38)	5583.51(3724.53,7574.34)	2248.70(1383.15,3212.68)	4682.52(3110.03,6356.67)	1170.22(697.27,1799.50)	3114.45(2051.29,4406.89)
Percentage of global, %	100	100	14.93	10.66	20.77	16.18	30.30	30.50	22.32	25.58	11.62	17.01
Percent change of YLDs 1990–2019, %	81.70(62.43,104.91)		29.82(13.74,46.95)		41.54(22.85,61.92)		82.89(58.31,113.35)		108.23(72.10,158.41)		166.14(116.21,240.30)	
**All-age YLDs rate**												
Rate per 100,000	188.31(123.45,265.09)	236.58(158.77,320.46)	182.93(117.28,253.70)	192.63(123.76,275.96)	181.90(119.92,247.68)	207.07(136.57,281.37)	177.84(115.21,249.04)	232.98(155.41,316.05)	199.06(122.44,284.40)	265.45(176.31,360.36)	221.57(132.02,340.72)	275.94(181.74,390.45)
Percent change of rate 1990–2019, %	25.63(12.31,41.68)		5.30(−7.74,19.2)		13.84(−1.20,30.23)		31.01(13.41,52.83)		33.35(10.21,65.49)		24.54(1.17,59.24)	
**Age-standardized YLDs rate**												
Rate per 100,000	187.88(122.92,263.21)	238.65(159.5,324.69)	184.30(117.87,255.91)	193.27(125.03,272.76)	182.86(120.82,247.78)	211.81(139.89,289.31)	177.93(115.72,248.19)	237.40(157.82,322.76)	201.38(125.12,286.98)	265.67(176.77,360.85)	227.78(137.75,348.71)	271.56(177.12,383.44)
Percent change of rate 1990–2019, %	27.02(14.32,42.25)		4.86(−7.36,17.51)		15.83(1.02,32.20)		33.42(15.46,55.61)		31.93(9.53,63.31)		19.22(−2.84,51.99)	
**APC model estimates**												
Net drift of YLDs, % per year	0.80(0.75,0.85)		0.46(0.44,0.48)		0.46(0.42,0.50)		0.93(0.88,0.98)		1.00(0.95,1.05)		0.56(0.49,0.62)	

**Figure 2:**
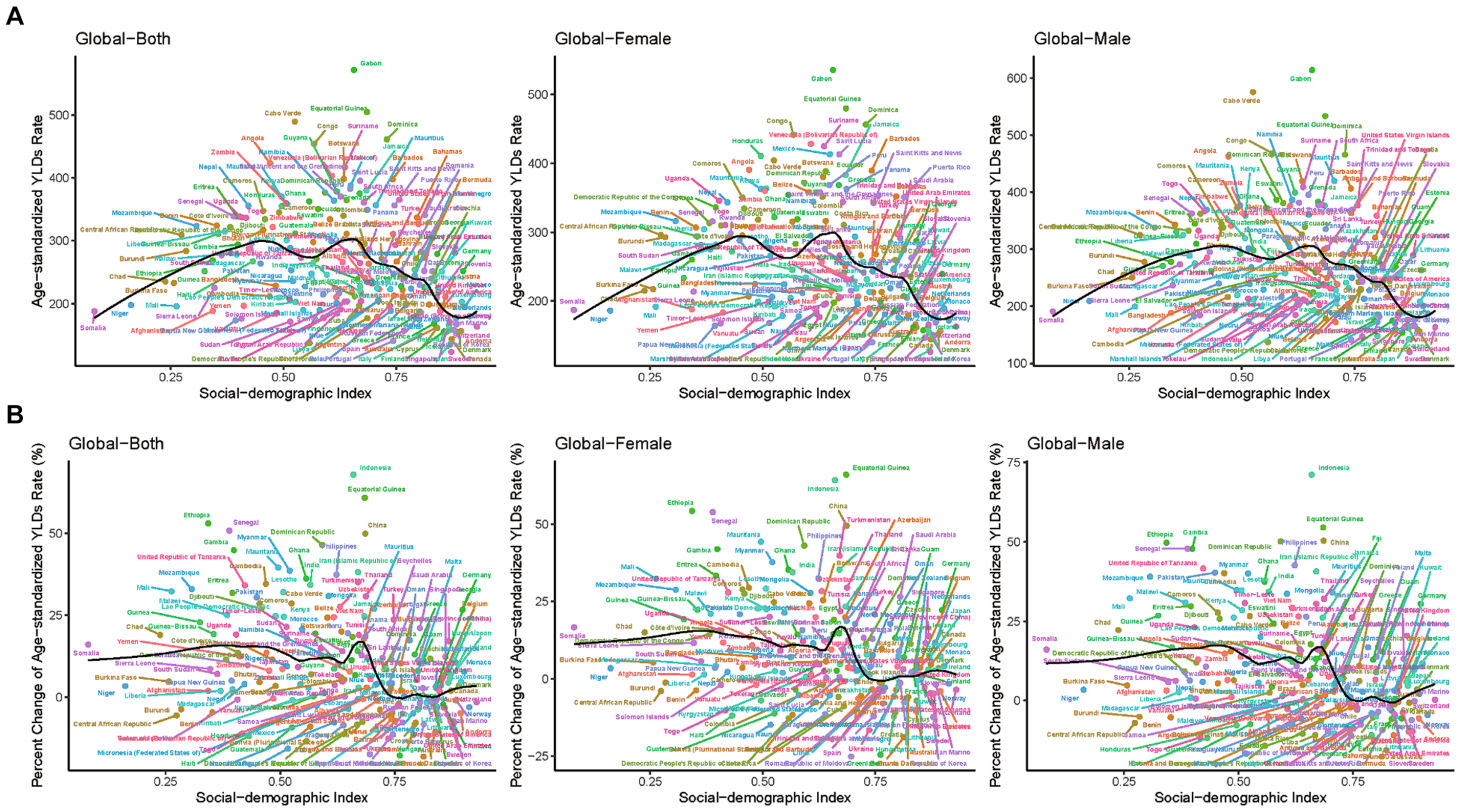
The ASR in 2019 **(A)** and percent change (%) in the ASR for epilepsy YLDs during 1990–2019 **(B)** for 204 countries and territories by SDI. ASR, age-standardized rates; SDI, socio-demographic index; YLDs, years lived with disability.

In addition, we also evaluated the relationship between the SDI and YLDs/prevalence from 1990 to 2019. With the increase of SDI level, the age-standardized YLDs rates showed a trend of increasing first and then gradually decreasing. With the increase of SDI level, the percent change (%) of the age-standardized YLDs rates showed a relatively stable trend, followed by a slight increase, and finally a gradual decrease. Similar patterns can be observed for both females and males, as shown in Figure 2 and Supplementary Figure S4.

### 3.2 Joinpoint regression analysis

**Figure 3:**
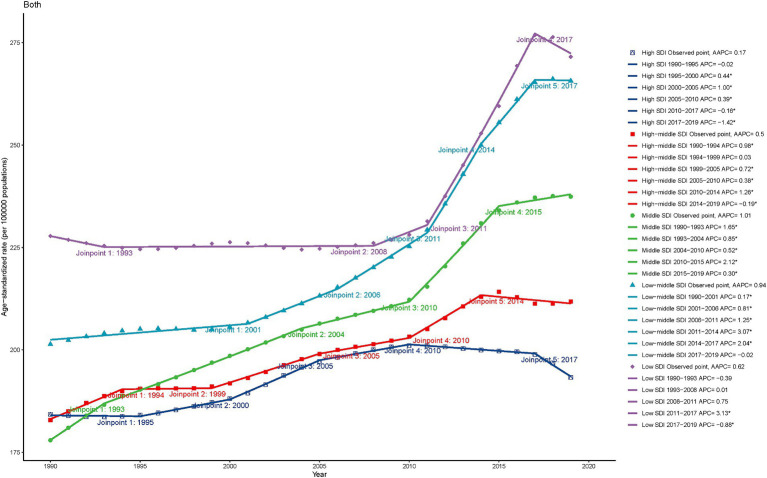
The Joinpoint regression analysis of the age-standardized YLDs rate for epilepsy by SDI quintiles, 1990–2019. YLDs, years lived with disability; SDI, socio-demographic index.

Figure 3 shows the trend of the age-standardized YLDs rates of epilepsy over time based on joinpoint regression analyses. In high-SDI countries, the age-standardized YLDs rate gradually increased from 1995 to 2010, gradually decreased after 2010, and abruptly decreased after 2017. In high-middle-SDI countries, the age-standardized YLDs rate has gradually increased since 1990, and gradually decreased after 2014. For middle-SDI and low-middle-SDI countries, the age-standardized YLDs rates have steadily increased until 2017. In low-SDI countries, the YLDs rate gradually increased after 2011, and began to decline in 2017. In general, the higher the SDI, the lower the YLDs rate. The trends for males and females in different SDI regions are generally consistent with each other, as represented in Supplementary Figure S5. The change of age-standardized prevalence rate showed a similar trend, as shown in Supplementary Figures S6, S7.

### 3.3 Age distribution of epilepsy YLDs and prevalence

**Figure 4:**
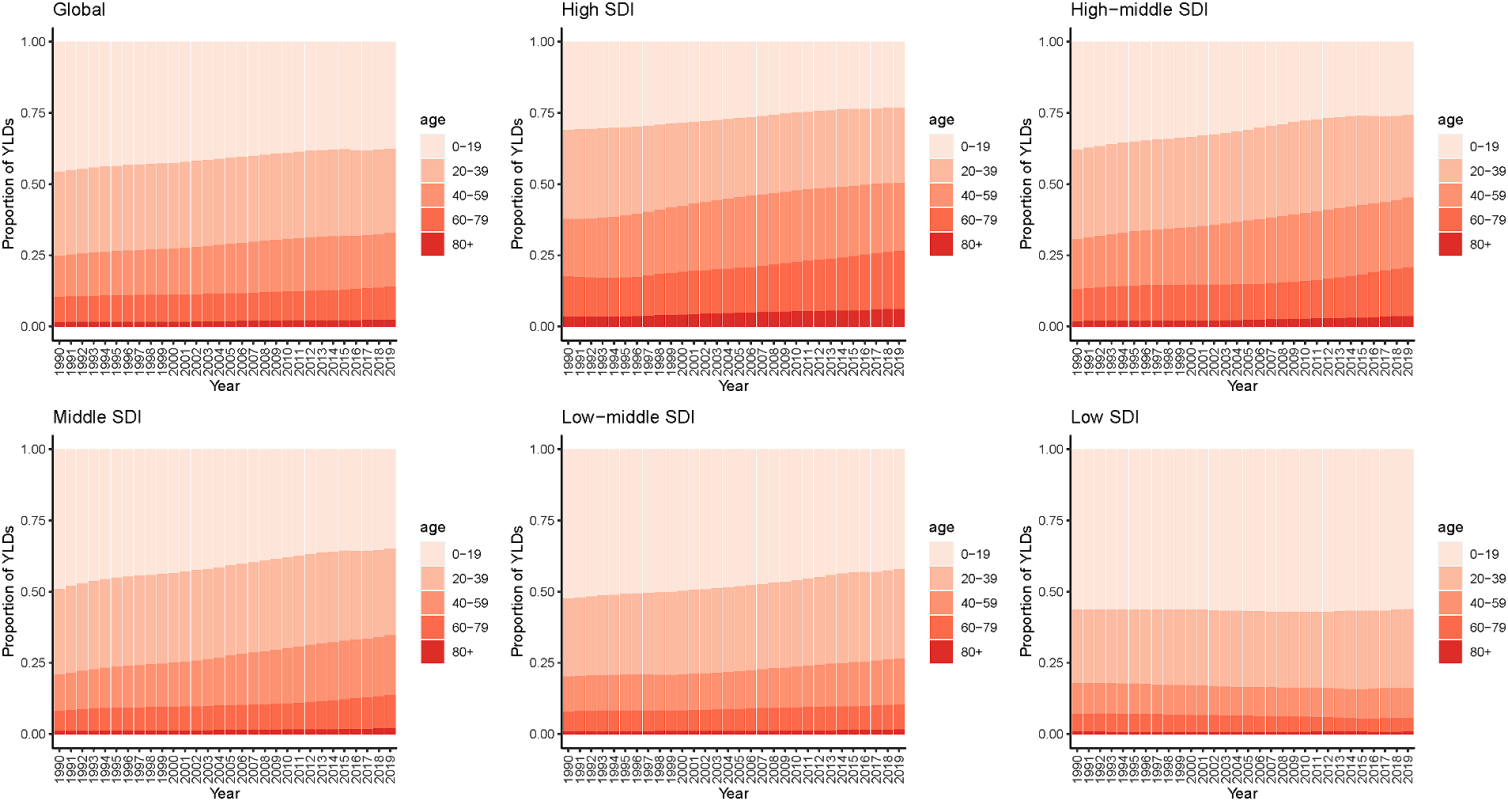
Age distribution of YLDs for epilepsy by SDI quintiles, 1990–2019. YLDs, years lived with disability; SDI, socio-demographic index.

Figure 4 shows the trend of age distribution of YLDs in epilepsy patients. Globally, from 1999 to 2019, the proportion of older age groups (>40 years) in YLDs gradually increased, with this trend becoming more pronounced in high-SDI, high-middle-SDI and middle-SDI countries. More than 80% of YLDs in low-SDI countries were concentrated in population under 40 years old. Prevalence showed a similar trend (Supplementary Figure S8). In addition, the results for both genders are generally consistent for prevalence and YLDs, as shown in Supplementary Figures S9, S10.

### 3.4 Age, period, and cohort effects

**Figure 5:**
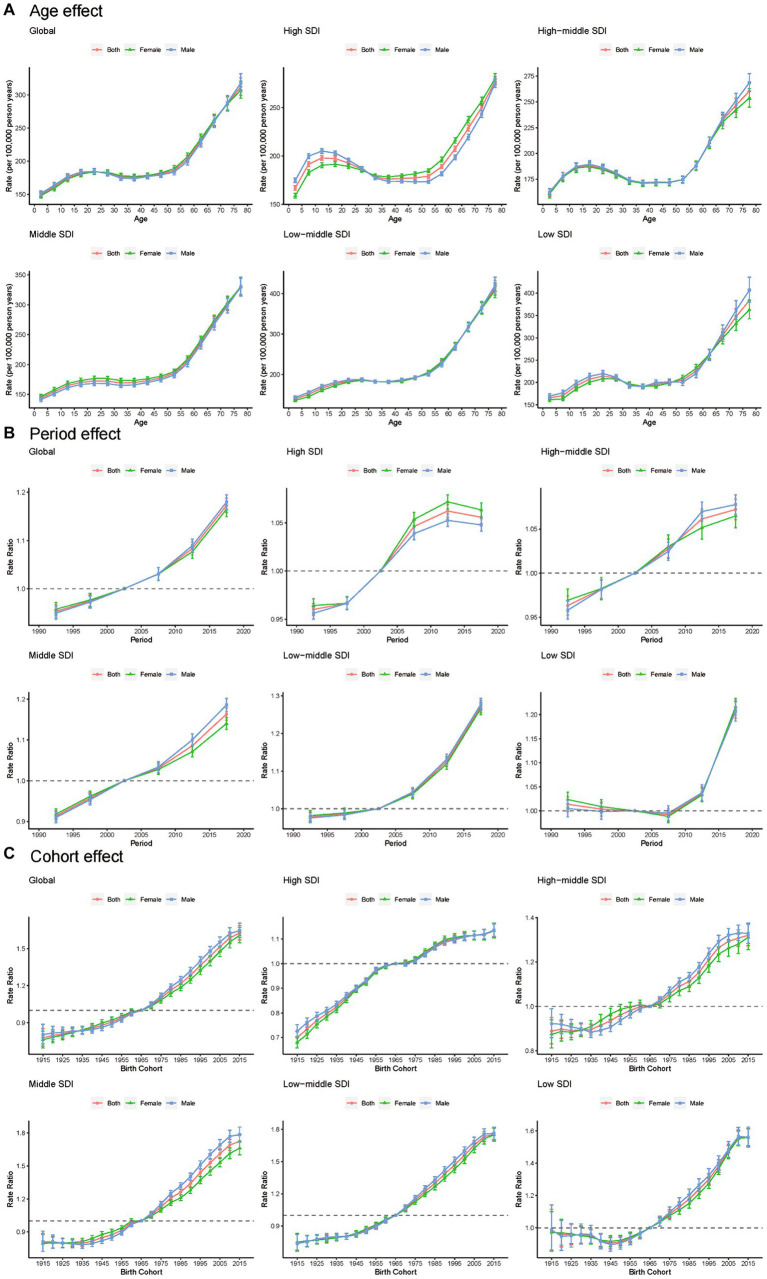
Age **(A)**, period **(B)** and cohort **(C)** effects on epilepsy YLDs by SDI quintiles. YLDs, years lived with disability; SDI, socio-demographic index.

Figure 5 shows the results analyzed using the APC model. The results shown that the risk of epilepsy YLDs increases with age, and no significant gender differences were found. Particularly, the age group aged 75–79 has the highest risk, while the age group aged 0–4 has the lowest risk. Further analysis of age-specific risks in different SDI countries revealed that the risk of epilepsy YLDs began to decrease before the age of 40 in high-SDI, high-middle-SDI, and low-SDI countries, respectively. Regarding the period effect, this study found that the risk of epilepsy YLDs increased with period. Further analysis of period-specific risks in different SDI countries revealed that the risk of epilepsy YLDs began to decrease after 2013 in high-SDI countries, while the risk of YLDs had not increased until after decade ago in low-SDI countries. In addition, we found that the period risk of middle- and low-middle-SDI countries was higher compared to high- and high-middle SDI countries. Regarding the birth cohort effect, this study found that the risk of epilepsy YLDs increases with the advancement of birth cohort time. Further analysis of birth cohort-specific risks in different SDI countries revealed that the YLDs risk showed a relatively slow decline in population born in 1915–1965 cohort in low-SDI countries, and increased significantly in population born after 1965 cohort. In particular, the relative risk of population born in the 2015 cohort was 1.14 (1.11–1.16) in high SDI countries and 1.75 (1.70–1.81) in medium low SDI countries.

**Figure 6:**
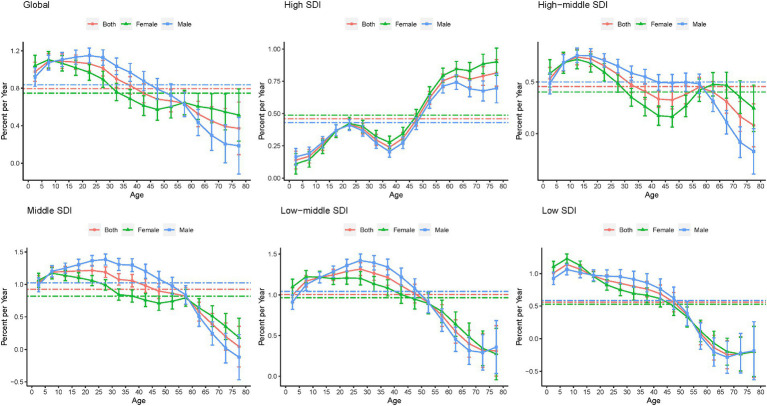
Local drifts in the YLDs of epilepsy by SDI quintiles, 1990–2019. YLDs, years lived with disability; SDI, socio-demographic index.

Figure 6 shows the local drift in the YLDs of epilepsy for each age group. Globally, epilepsy YLDs had increasing trends across all age groups, especially the 5–9 age group (1.089% [1.008–1.171] per year). Among pediatric population aged 0–19, YLDs increase was the lowest in high-SDI countries (0.140% [0.071–0.209] in children 0–4 years; 0.369% [0.325–0.413] in those 15–19 years). Among the population over 60 years old, YLDs increase was the greatest in high-SDI countries (0.795% [0.740–0.850] in those aged 60–64 years; 0.815% [0.712–0.918] in those 75–79 years). Moreover, in the low-SDI countries, epilepsy YLDs show a trends in those aged over 60 years. Prevalence showed a similar trend with YLDs in APC model, as shown in Supplementary Figures S11, S12.

### 3.5 APC effects in exemplary countries

**Figure 7:**
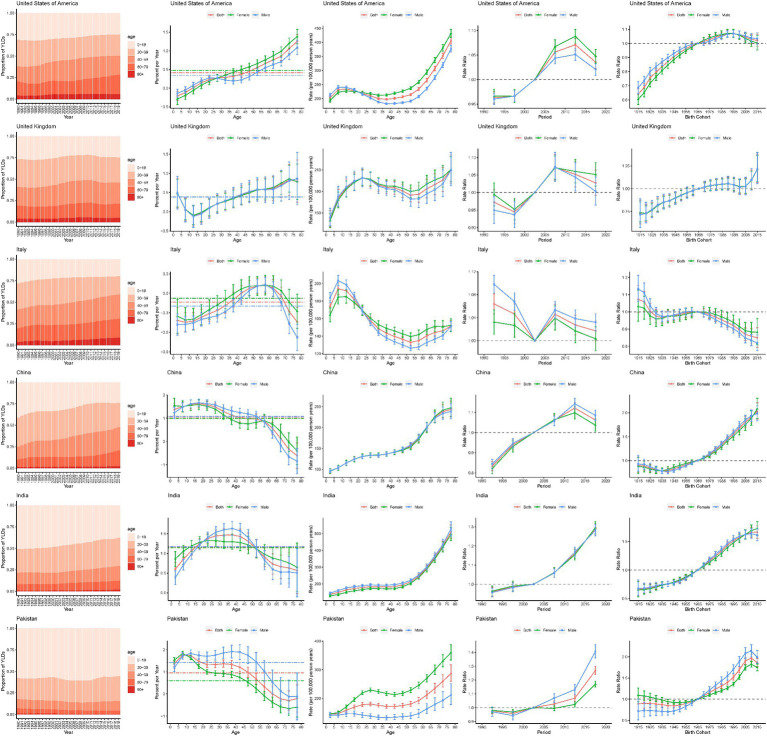
Age, period and cohort effects on epilepsy YLDs for representative countries. YLDs, years lived with disability.

To further analyze the age-period-cohort effects of different SDI countries, this study selected exemplary countries with different SDIs to analyze the major trends in epilepsy YLDs and prevalence. As shown in Figure 7, epilepsy YLDs in the United States of America was a typical trend in high-SDI countries. In the past 30 years, the proportion of epilepsy YLDs in older patients has been increasing, and the risk of epilepsy YLDs was positively correlated with age, period, and birth cohort. However, risk of YLDs showed a declining trend in the past 5 years and in those born after the 1995 cohort. Age-standardized YLDs rate gradually increased during the period 1995–2010, and then began to decline in 2010.

United Kingdom (UK) was one of the high-SDI countries. Except for children aged 10–19, epilepsy YLDs have increased in all age groups. Unfavorable increasing cohort risks were showed. The risk in the UK decreased from 1990 to 1999 and from 2010 to 2019, and increased from 2000 to 2009. Over the past 30 years, United Kingdom ASR YLDs rate has changed unsteadily, with an upward trend after 2016.

Italy was one of the high-middle SDI country. This study found that the proportion of patients with epilepsy YLDs aged 40 and above was increasing, especially in the age group of 40–64, while the proportion of YLD in the age group of 64 and above was decreasing. The risks of YLDs in cohorts born from 1930 to 1980 were relatively smooth, but the risks of YLDs cohorts born after 1980 have decreased. YLDs rates gradually decline with age, among the ages of 10 and 55.

China was one of the middle-SDI country. This study found that the proportion of epilepsy YLDs in older patients has been increasing, and the YLDs rate gradually increased with age. Age-standardized YLDs rate has been rising steadily since 1990 and did not begin to decline until 2014. In addition, the risks of YLDs in cohorts born after 1980 have increased.

India was one of the low-middle-SDI country. This study found that the YLDs rate gradually increased with age, and age-standardized YLDs rate has shown a gradual increase over the past three decades. Moreover, the risk significantly increases over the periods and birth cohorts.

Pakistan was one of the low-SDI country. This study found that age proportion of epilepsy YLDs has been no significant change in the last 30 years. The YLDs in the population aged 0–65 significantly increased, while YLDs in populations aged 65 and above significantly decreased. In general, YLDs rate increased with age. The rate of age-standardized YLDs has shown a gradual upward trend since 1994, and has gradually declined in the last 2 years. Moreover, the risk significantly increases over the periods and birth cohorts.

Prevalence showed a similar trend with YLDs in APC model in exemplary countries (Supplementary Figure S13).

### 3.6 National trends in epilepsy YLDs and prevalence

Analyzing the epilepsy YLDs in different countries from 1990 to 2019, it was found that the YLDs in most countries and territories (146/204) have showed an increasing trend (Supplementary Table S4). Among them, Ethiopia had the highest increase in YLDs rate (68.66% [10.44–192.82]) In high-SDI countries, the all-age and age-standardized YLDs rates of epilepsy has generally decreased or moderately increased in North America, Europe, and Asia-Pacific regions, but it has increased abnormally high in Germany (29.97% [−43.28–181.8] and 26.64% [−39.89–162.04]), with a net drift of YLDs at 1.2% (1.16–1.25) per year. In low-SDI countries, Ethiopia showing notable increase in the number of YLDs (253.09% [131.21–513.04]), with the increase in all-age and age-standardized YLDs rates (68.66% [10.44–192.82] and 53.03% [1.68–160.45]). In South Africa, all-age and age-standardized YLDs rates increased by 16.54% (−7.56–50.66) and 12.8% (−10.2645.63), respectively, but the net drift of YLDs was only −0.12% (−0.22–−0.03) per year. In 2019, the highest YLDs rate for all age groups was in the Dominica, Cabo Verde, Equatorial Guinea and Gabon, while the lowest YLDs rate were in Sweden, Japan, North Korea, and Canada. Moreover, the all-age YLDs rate and age-standardized YLDs rate of the three countries (Cape Verde, Equatorial Guinea, and Gabon) were more than twice higher than the global average.

Analyzing the epilepsy YLDs and prevalence in different countries from 1990 to 2019, it was found that the YLDs in most countries and territories (146/204) have showed an increasing trend (Supplementary Table S5). Among them, Equatorial Guinea had the highest increase in prevalence (81.97% [−10.17–268.71]), while the Republic of Moldova had the largest decrease in all-age prevalence rate (−14.9% [−44.14–34.17]). In low-SDI countries, Ethiopia showing notable increase in the number of prevalence (263.74% [155.85–477.59]), with the increase in all-age prevalence rate (73.74% [22.21–175.89]). In high-SDI countries, a decrease or moderate increase in all-age and age-standardized prevalence rates was generally observed, but in Germany, the increase in prevalence rates and standardized prevalence rates for all ages and ages is exceptionally high, but the increase in the number of prevalence (53.57% [−32.57–216.4]), all-age and age-standardized prevalence rates (44.58% [−36.52–197.88] and 38.68% [−34.93–175.29]) in Germany was abnormally high, with a net drift of prevalence at 1.48% (1.43–1.54) per year. In 2019, the highest all-age prevalence was in the Gabon, Cabo Verde, Dominica and Equatorial Guinea, while the lowest all-age prevalence were in North Korea, Sweden, Japan and Papua New Guinea. Moreover, the all-age prevalence in 26 countries and age-standardized prevalence in 24 countries was more than twice higher than the global average.

Overall, these results suggest that trends in epilepsy YLDs and prevalence are uneven across countries. The change in YLDs and prevalence in countries may not be fully commensurate with the expected conditions of their corresponding SDI. In addition, the trend of change in YLDs and prevalence rates shown by traditional measurement methods (all-age and age-standardized rates) may not be exactly consistent with the change shown by the APC models, indicating the need to distinguish the period and cohort trends in YLDs and prevalence.

## 4 Discussion

Our findings suggested a gradual increase in the burden of epilepsy from 1999 to 2019, the prevalence and YLDs of epilepsy in the Global and all SDI quintiles countries and territories increased in 2019. The net drift of prevalence and YLDs of epilepsy was >0% globally and in all SDI quintiles countries and territories. The risk of epilepsy prevalence and disease burden increases with age. Similarly, the risk of epilepsy prevalence and disease burden increases with the increase of time period and cohort time, especially in middle-SDI, low-middle-SDI and low-SDI quintiles countries and territories compared with the previous GBD 2016 publication, this study provides more detailed analysis of disease trends and further uses the analysis results to generate public health insights for disease management (7, 20). Specifically, this includes estimating local drift to understand the changing trends in prevalence and YLDs rate for each age group, and revealing the period and cohort effects that affect the temporal trends of prevalence and YLDs rates to provide information on the effectiveness of epilepsy related healthcare services.

This study found that in 2019, the age-standardized prevalence and age-standardized YLDs of epilepsy in low-middle-SDI and low-SDI countries were higher than those in other countries. Program launched in Pakistan in recent years to reduce the high epilepsy treatment gap and stigma have had good success. This activity increased public awareness of epilepsy, which may have further improved the diagnosis of epilepsy. This may explain the increased prevalence of epilepsy in Pakistan. However, many risk factors that contribute to the gap in epilepsy treatment have not been effectively addressed, especially since epilepsy has rarely become a public health priority in low-SDI countries. The World Health Organization (WHO) reported that the epilepsy treatment gap in most low-income countries exceeds 75%, while the epilepsy treatment gap in middle-income countries exceeds 50% (21). As in most low-SDI and lower-middle-SDI countries, epilepsy treatment is a serious economic burden in Pakistan due to government neglect and insufficient health budgets (22, 23). Moreover, the lack of highly skilled clinical experts, the poor quality of medical and health services, and the poor nutritional status of residents have jointly exacerbated the health loss. This may be the reason for the severe health loss of epilepsy in Pakistan. Therefore, it is urgent to develop appropriate strategies to strengthen the attention of low-SDI countries to epilepsy, and popularize the diagnosis and treatment plan for epilepsy, and narrow the treatment gap.

China is one of the middle-SDI countries with over 1.4 billion inhabitants. This study found that YLDs and prevalence has been rising steadily since 1990 and did not begin to decline until 2014. The net drift of YLDs and prevalence are 1.04 and 1.23%, respectively. The prevalence was determined by the incidence rate and mortality (24). Therefore, the significant increase in epilepsy prevalence in China may be related to the increase in life expectancy and rapid aging process in China (25, 26). Over the past three decades, the treatment of epilepsy in China has improved rapidly, with nearly 20 different anti-epileptic drugs now available, as well as non-pharmacological treatment options, but there is still an unmet need in epilepsy management (27). In China, due to a lack of proper understanding and stigmatization related to epilepsy diagnosis, and due to financial constraints, these factors may affect the patient’s personal medical behavior and compliance (28). In addition, the lack of electroencephalogram and neuroimaging equipment, and personnel with neurology expertise in primary medical institutions also greatly limits the diagnosis and treatment of patients. This may indicate the need to continue to increase public awareness of epilepsy, increase financial support for epilepsy, and further reduce the health loss caused by epilepsy.

For United States of America, the changes in epilepsy YLDs rate and prevalence in 2019 were not significant compared to 1990. In general, YLDs rate and prevalence gradually increased from 1999 to 2009, and then began to decline in the recent 5 years. According to the CDC (29), the increase in epilepsy prevalence may be related to population growth and increasing levels of aging, or other unknown factors (such as the increasing number of people willing to disclose that they have epilepsy). Immigration from high-prevalence areas of epilepsy, such as Latin America, may also be one of the reasons for the increase of epilepsy prevalence rate in America.

The prevalence and YLDs rates of epilepsy increase with age, with the highest prevalence and YLDs in the older adults. In particular, as countries and territories with higher-SDI experience increasing population aging, the proportion of older adults people in epilepsy prevalence and mortality was increasing in these countries and territories. This may be associated with more complications in the older adults. Studies have found that metabolic diseases such as high fasting blood glucose, high body mass index, and high systolic blood pressure can increase the risk of stroke, Alzheimer’s disease, and other neurological diseases, and many neurological diseases are associated with epilepsy (12, 30). With the steady growth of the rapid increase in the number of older adults people, epilepsy will gradually become one of the great burdens of society in the future. Therefore, it is necessary to increase research on the occurrence and development of epilepsy in the older adults, improve the diagnosis and treatment effectiveness of older adults epilepsy patients, and reduce their burden.

There are several limitations in this study. First, like other studies based on the GBD data, it was not possible to fully reflect the global prevalence and disease burden of epilepsy due to the fact that epilepsy disease data in some low-income and middle-income countries in the GBD data were predictive data rather than raw data. Secondly, the reliability of the research results depends on the completeness and robustness of previous research work due to this study was a secondary study. Thirdly, this study only analyzed the prevalence and YLDs data of epilepsy at the national level, and cannot further analyze the trend of disease burden changes in epilepsy in different regions of the country. In conclusion, more comprehensive and high-quality collection of raw data is the fundamental measure to address these limitations.

Although there are differences in the prevalence and YLDs of epilepsy among different countries and territories, the prevalence and YLDs of epilepsy has shown an increasing trend worldwide, and the disease burden was still increasing, which may be mainly attributed to the population growth and aging. In order to address the disease burden caused by epilepsy, this study proposes the following solutions. Firstly, highlighting the importance of healthy aging for the burden of epilepsy in older adults, following resolution WHA 73.10 and the Intersectoral Global Action Plan on Epilepsy and Other Neurological Disorders (IGAP), countries and regions need to strengthen epilepsy prevention efforts. Secondly, strengthen health education on epilepsy, increase public awareness of epilepsy, and prevent people with epilepsy and their families becoming poorer and more marginalized due to the misconceptions and negative attitudes that surround epilepsy. Thirdly, optimize the diagnosis and treatment plan for epilepsy in the older adults, especially for those with other complications, to improve the diagnosis and treatment effectiveness of older adults epilepsy patients and reduce the disease burden. Fourthly, popularize epilepsy diagnosis and treatment plans in economically underdeveloped areas to narrow the treatment gap.

## Data availability statement

Publicly available datasets were analyzed in this study. This data can be found at: https://ghdx.healthdata.org/gbd-results-tool.

## Author contributions

TS: Formal analysis, Writing – original draft. YZ: Formal analysis, Writing – original draft. HF: Writing – original draft, Methodology, Writing – review & editing. ZL: Investigation, Supervision, Writing – review & editing. JX: Methodology, Visualization, Writing – review & editing. ML: Supervision, Validation, Writing – review & editing. SJ: Supervision, Validation, Writing – review & editing.
