# Supplementary material for: Global, regional, and national time trends in the burden of epilepsy, 1990–2019: an age-period-cohort analysis for the global burden of disease 2019 study
Source: Front Neurol. 2024 Aug 15;15:1418926. doi: 10.3389/fneur.2024.1418926 (PMC11373253; doi:10.3389/fneur.2024.1418926)
Supplement: Supplementary file 1 [file Data_Sheet_1.docx]

**SUPPLEMENT**

**Figure S1. The ASR in 2019 (A) and percent change (%) of the ASR during 1990-2019 (B) for epilepsy prevalence for both gender.**

**Figure S2. The ASR in 2019 (A) and percent change (%) of the ASR during 1990-2019 (B) for epilepsy YLDs for Female and Male.**

**Figure S3. The ASR in 2019 (A) and percent change (%) of the ASR during 1990-2019 (B) for epilepsy prevalence for Female and Male.**

**Figure S4. The ASR in 2019 (A) and percent change (%) in the ASR for epilepsy prevalence during 1990-2019 (B) for 204 countries and territories by SDI.**

**Figure S5. The Joinpoint regression analysis of the age-standardized YLDs rate for epilepsy by SDI quintiles for Female and Male, 1990-2019.**

**Figure S6. The Joinpoint regression analysis of the age-standardized prevalence rate for epilepsy by SDI quintiles for both gender, 1990-2019.**

**Figure S7. The Joinpoint regression analysis of the age-standardized prevalence rate for epilepsy by SDI quintiles for Female and Male, 1990-2019.**

**Figure S8. Age distribution of prevalence for** **epilepsy by SDI quintiles for both gender, 1990-2019.**

**Figure S9. Age distribution of YLDs for epilepsy by SDI quintiles for Female and Male, 1990-2019.**

**Figure S10. Age distribution of prevalence for epilepsy by SDI quintiles for Female and Male, 1990-2019.**

**Figure S11. Age, period and cohort effects on epilepsy prevalence by SDI quintiles.**

**Figure S12. Local drifts in the prevalence of epilepsy by SDI quintiles, 1990-2019.**

**Figure S13. Age, period and cohort effects on epilepsy prevalence for representative countries.**

**Table S1. Trends in epilepsy Prevalence for both genders across SDI quintiles, 1990−2019.**

**Table S2. Trends in epilepsy YLDs for Male and Female genders across SDI quintiles, 1990−2019.**

**Table S3. Trends in epilepsy Prevalence for Male and Female genders across SDI quintiles, 1990−2019.**

**Table S4. Trends in epilepsy YLDs for both genders in 204 countries and regions, 1990-2019.**

**Table S5. Trends in epilepsy Prevalence for both genders in 204 countries and regions, 1990-2019.**

**Figure S1. The ASR in 2019 (A) and percent change (%) of the ASR during 1990-2019 (B) for epilepsy prevalence for both gender.**

**
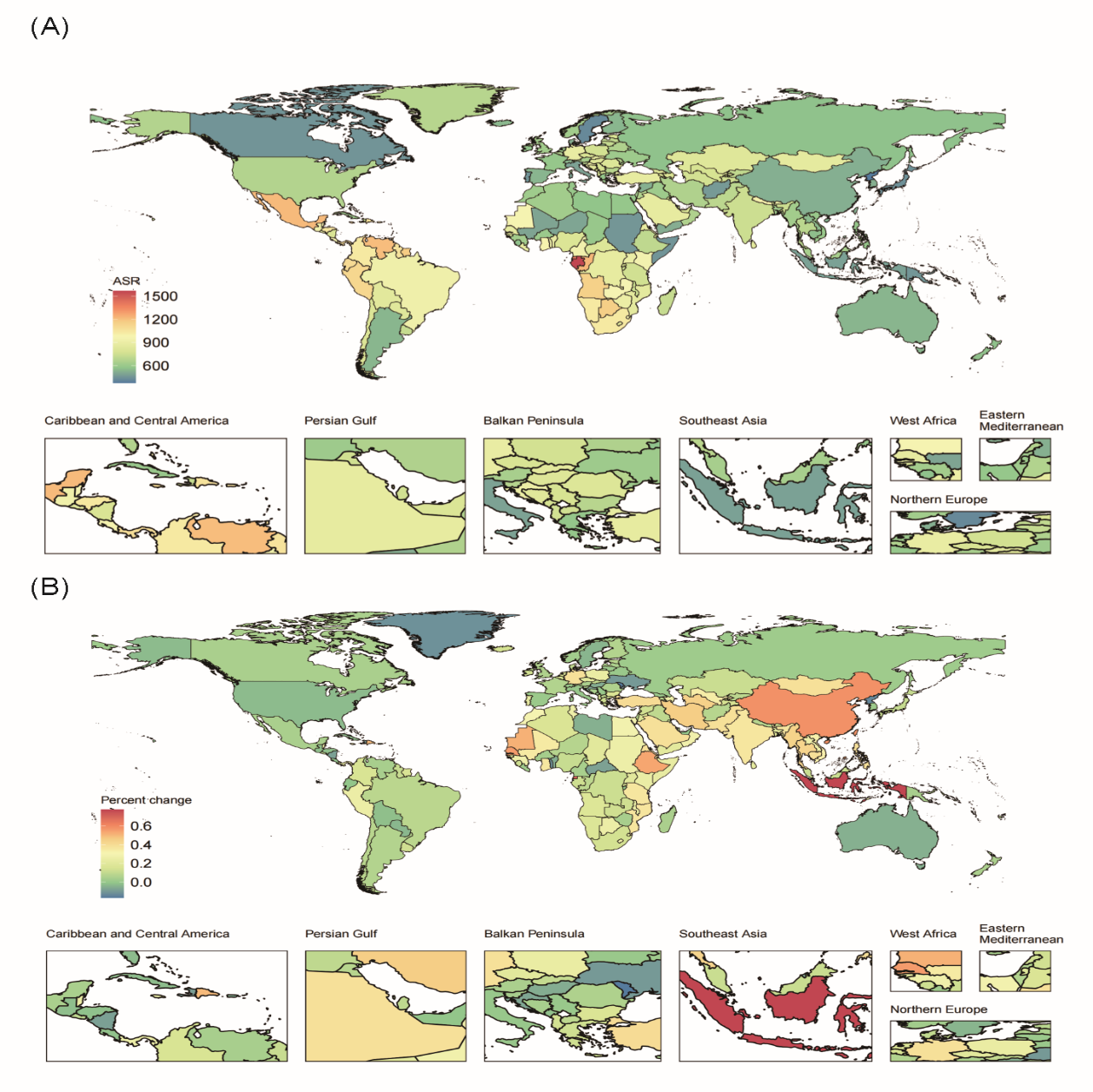
**

**Figure S2. The ASR in 2019 (A) and percent change (%) of the ASR during 1990-2019 (B) for epilepsy YLDs for Female and Male.**

**
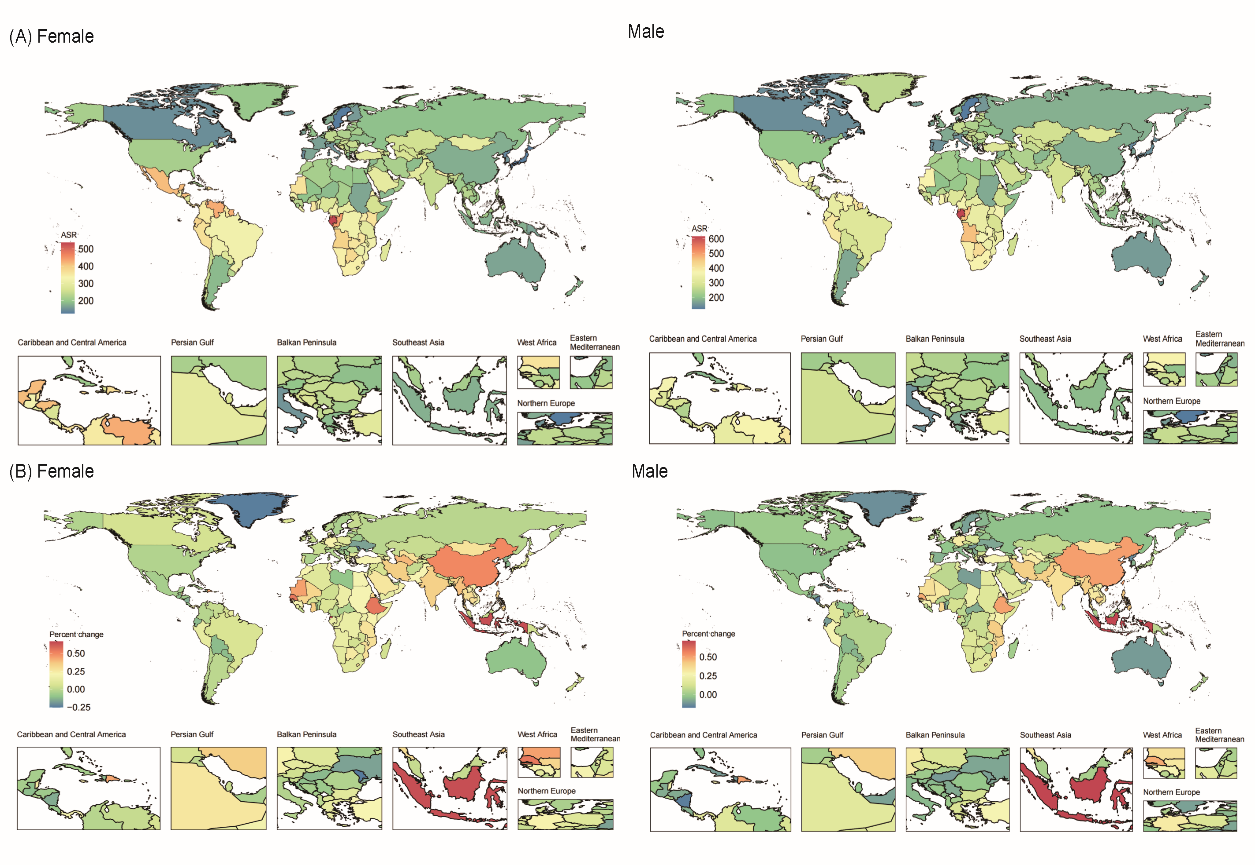
Figure S3. The ASR in 2019 (A) and percent change (%) of the ASR during 1990-2019 (B) for epilepsy prevalence for Female and Male.**

**
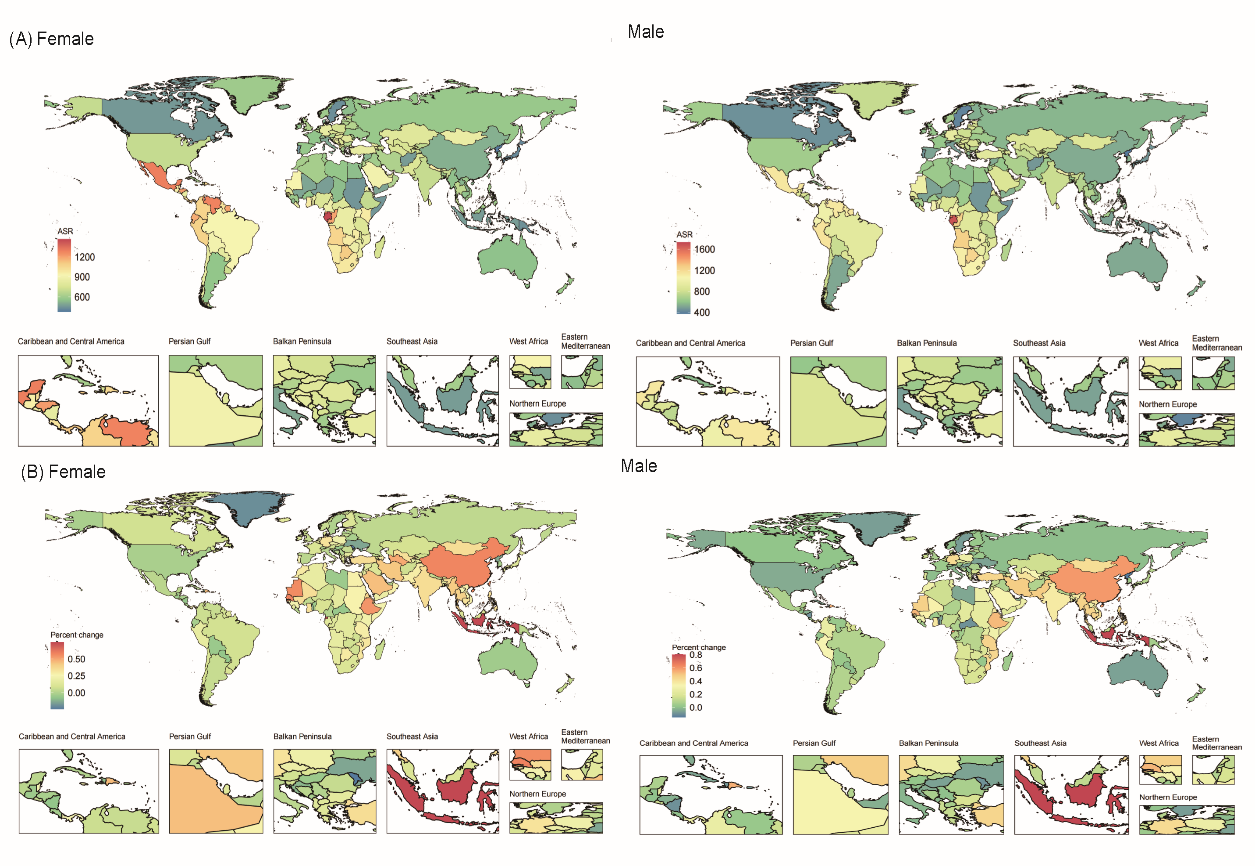
**

**Figure S4. The ASR in 2019 (A) and percent change (%) in the ASR for epilepsy prevalence during 1990-2019 (B) for 204 countries and territories by SDI.**

**
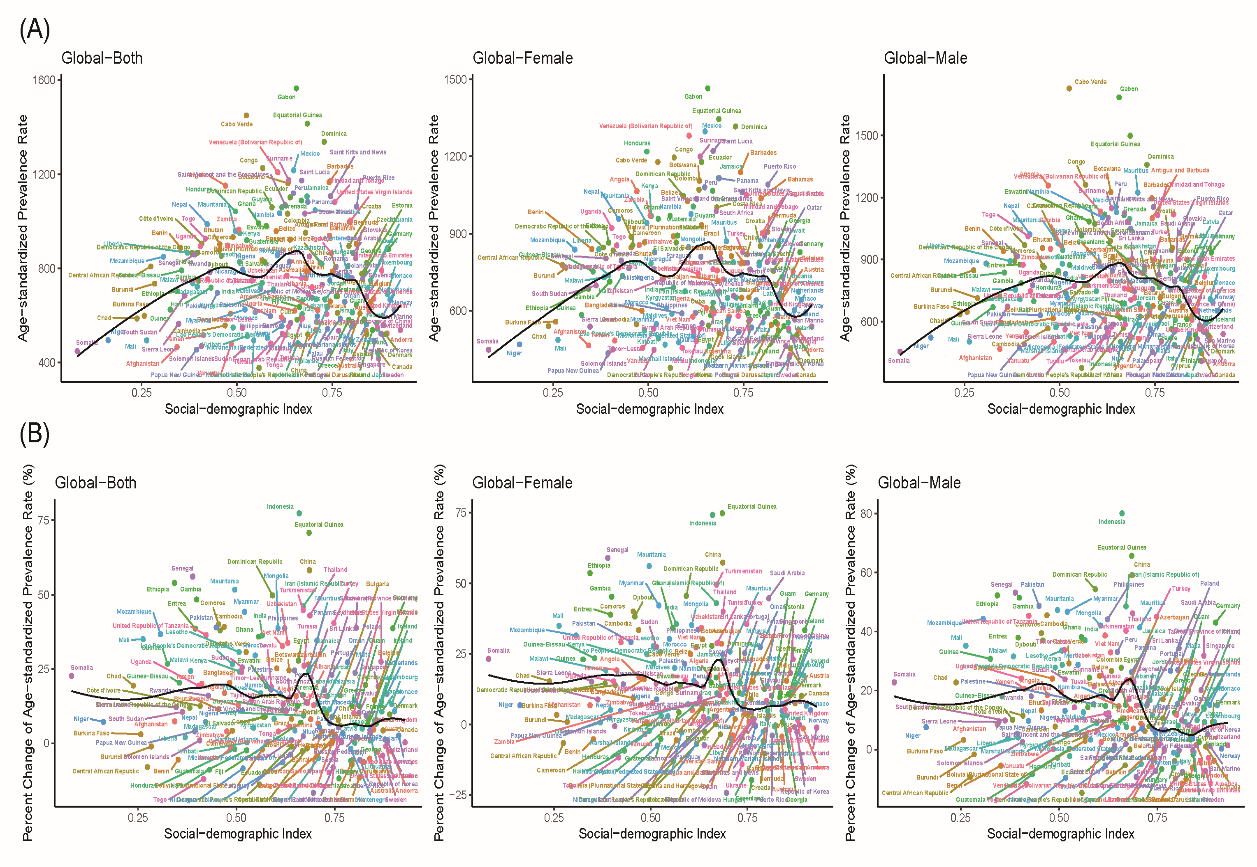
**

**Figure S5. The Joinpoint regression analysis of the age-standardized YLDs rate for epilepsy by SDI quintiles for Female and Male, 1990-2019.**

**
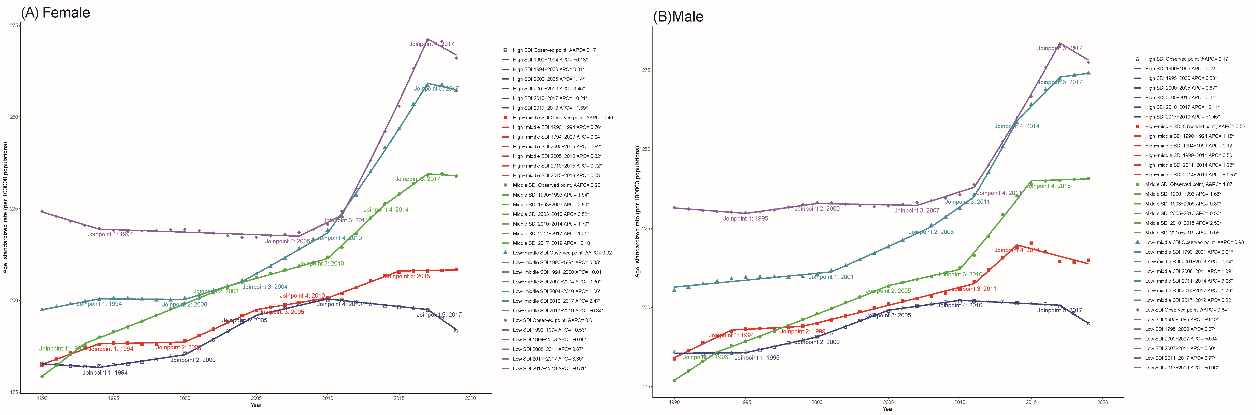
**

**Figure S6. The Joinpoint regression analysis of the age-standardized prevalence rate for epilepsy by SDI quintiles for both gender, 1990-2019.**

**
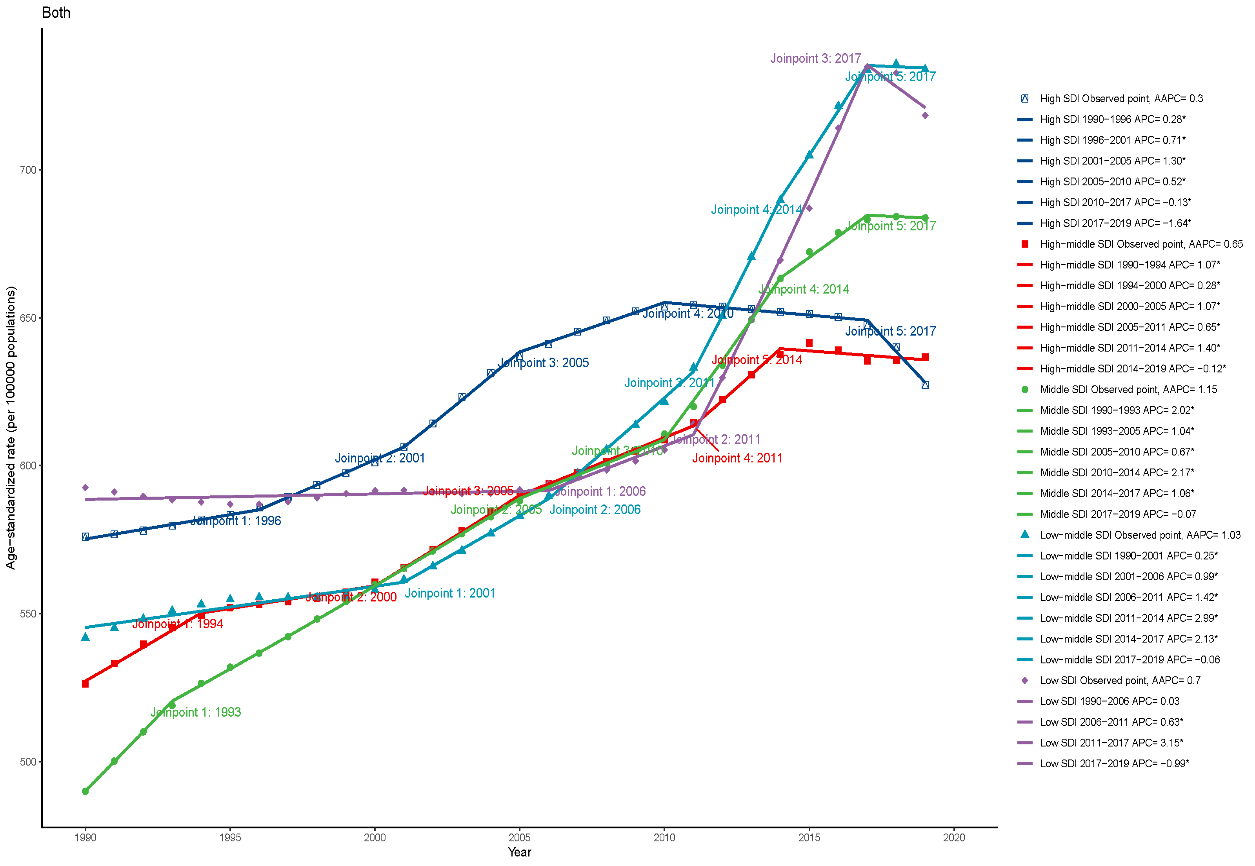
**

**Figure S7. The Joinpoint regression analysis of the age-standardized prevalence rate for epilepsy by SDI quintiles for Female and Male, 1990-2019.**

**
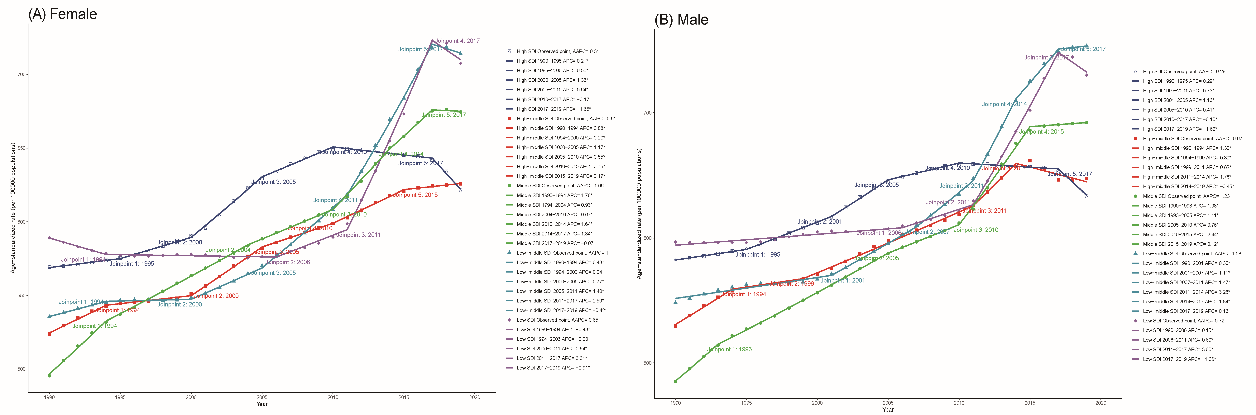
**

**Figure S8. Age distribution of prevalence for epilepsy by SDI quintiles for both gender, 1990-2019.**

**
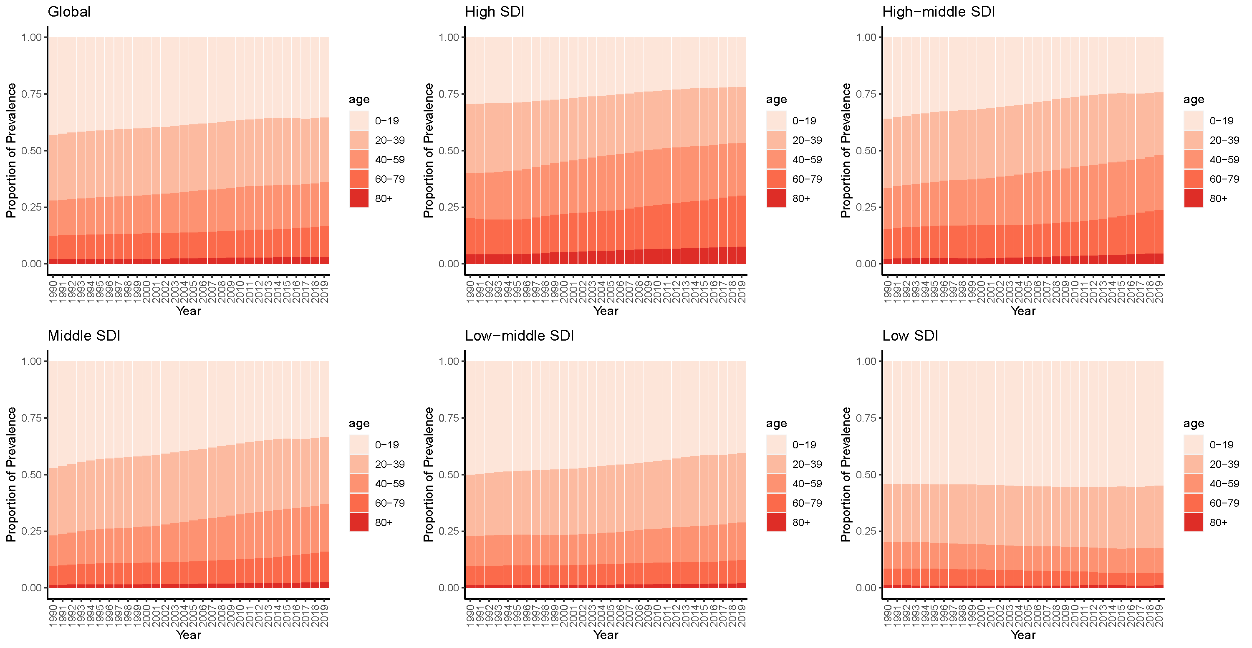
**

**Figure S9. Age distribution of YLDs for epilepsy by SDI quintiles for Female and Male, 1990-2019.**

**
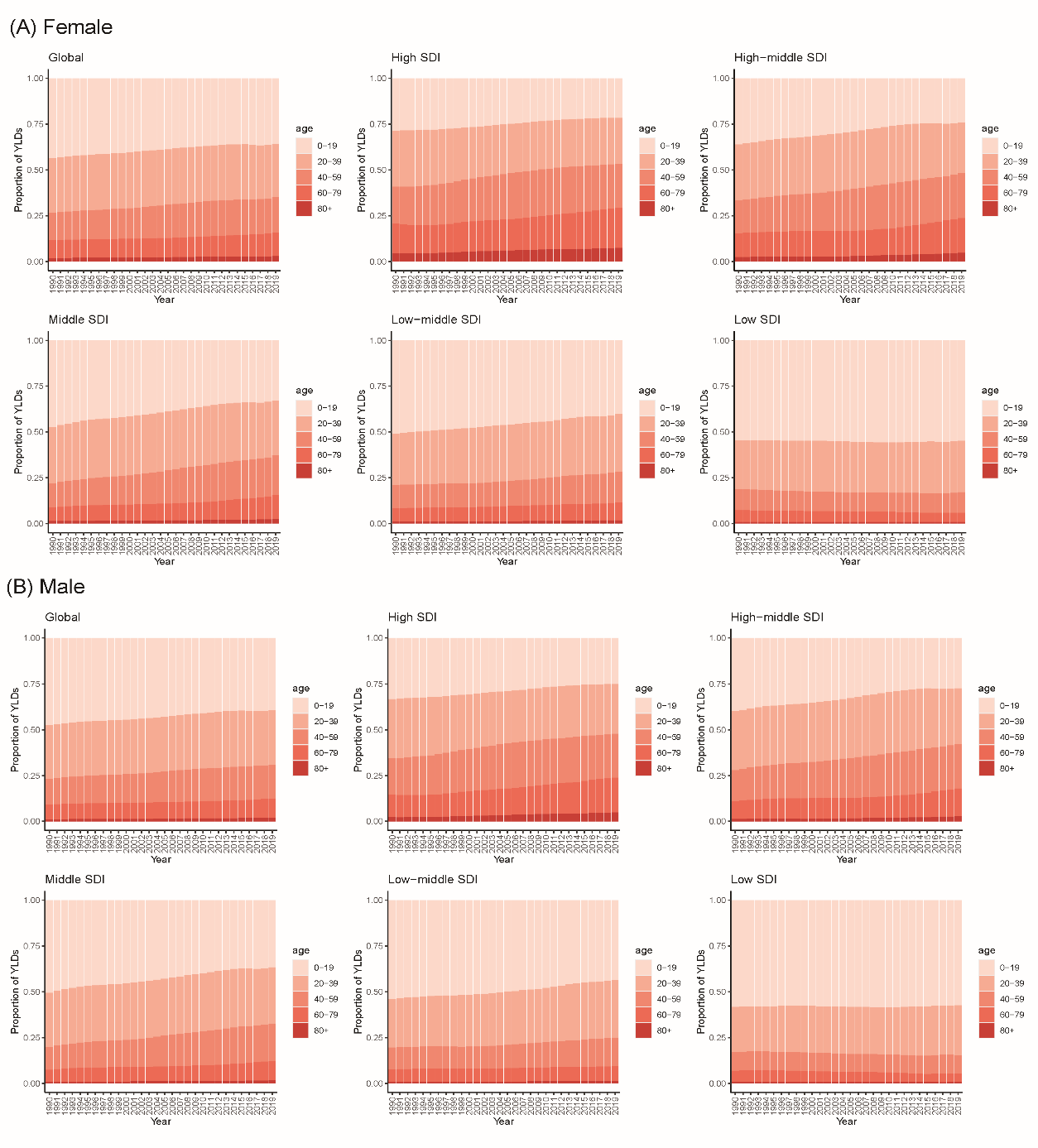
**

**Figure S10. Age distribution of prevalence for epilepsy by SDI quintiles for Female and Male, 1990-2019.**

**
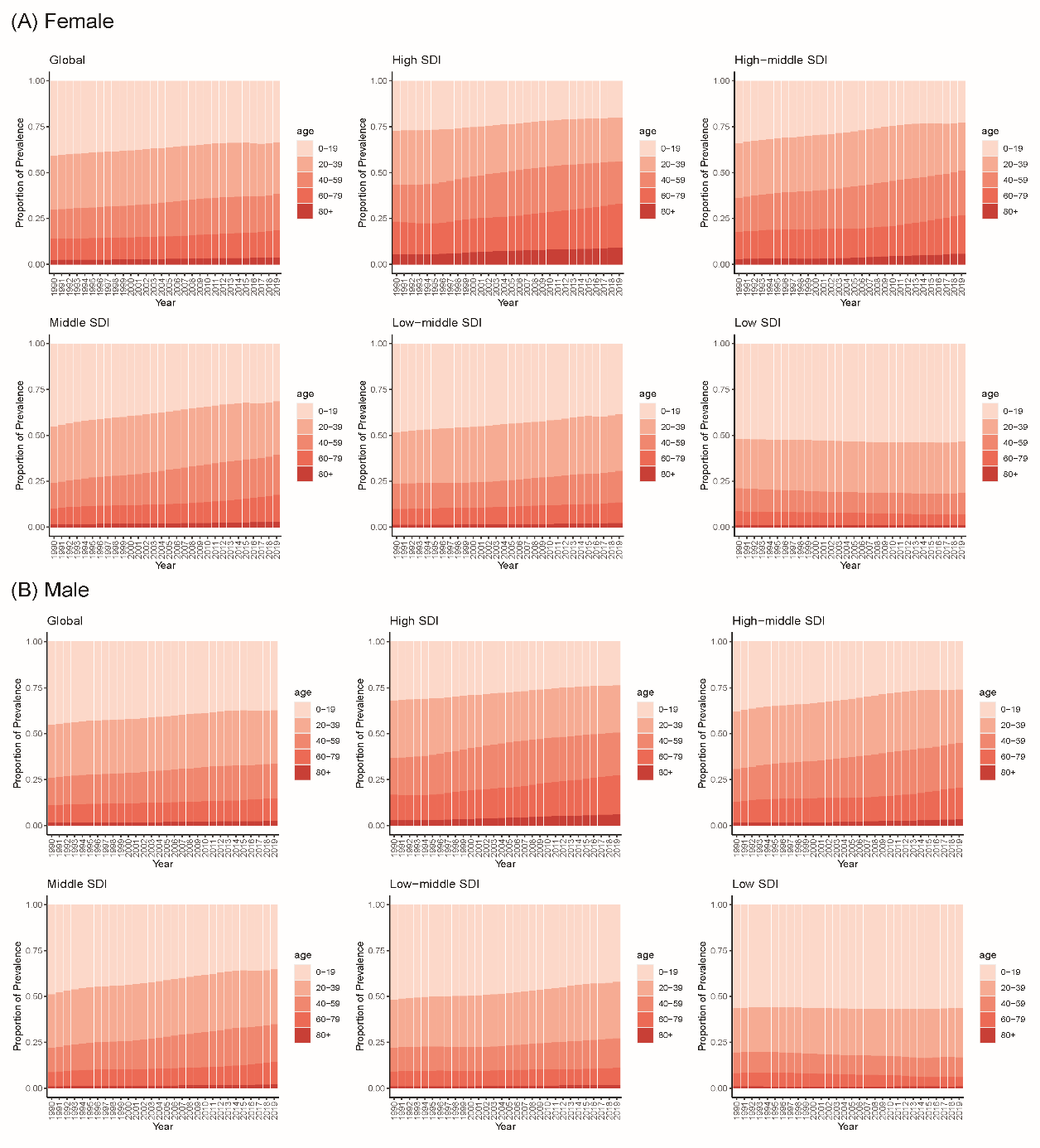
**

**Figure S11. Age, period and cohort effects on epilepsy prevalence by SDI quintiles.**


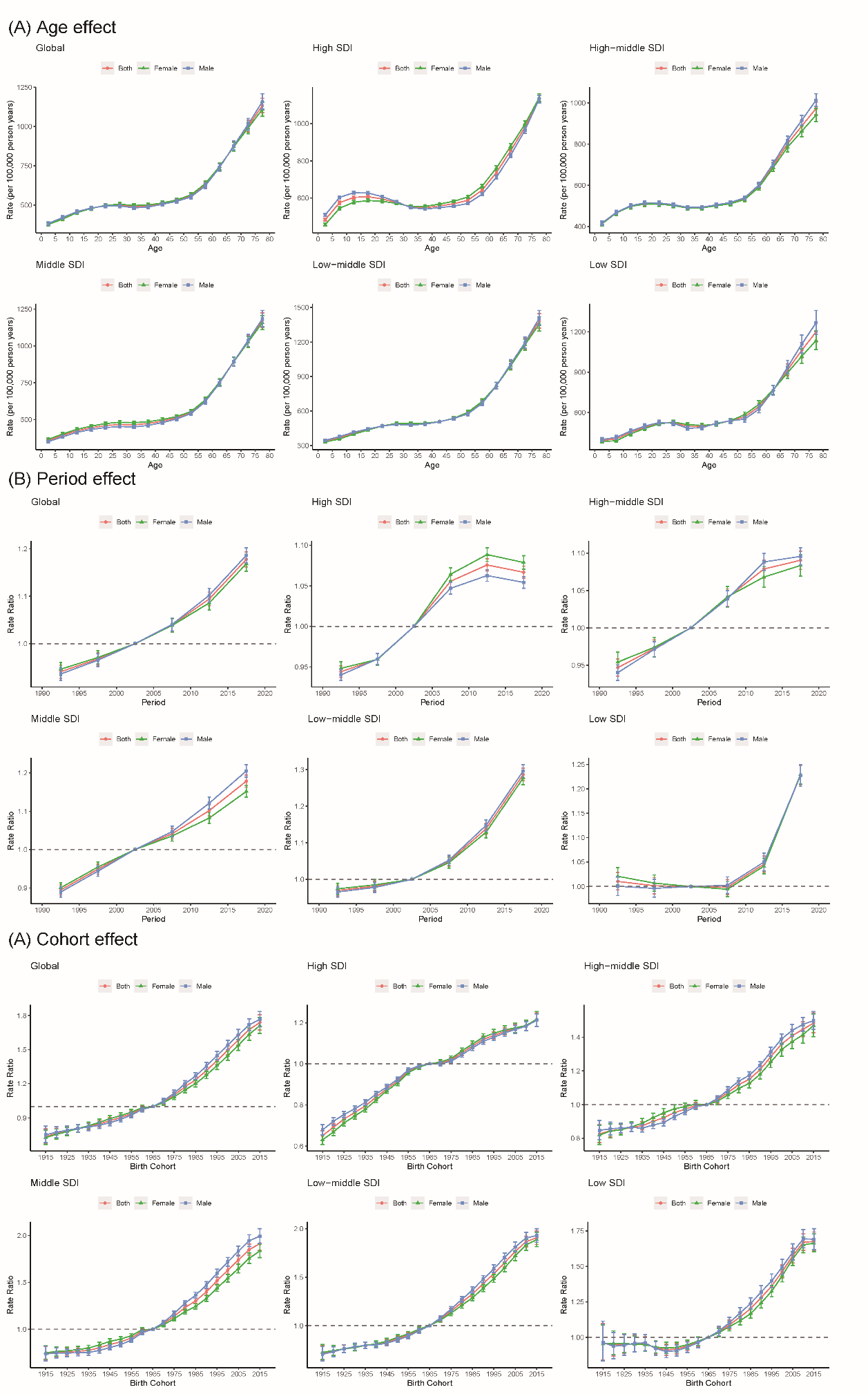


**Figure S12. Local drifts in the prevalence of epilepsy by SDI quintiles, 1990-2019.**

**
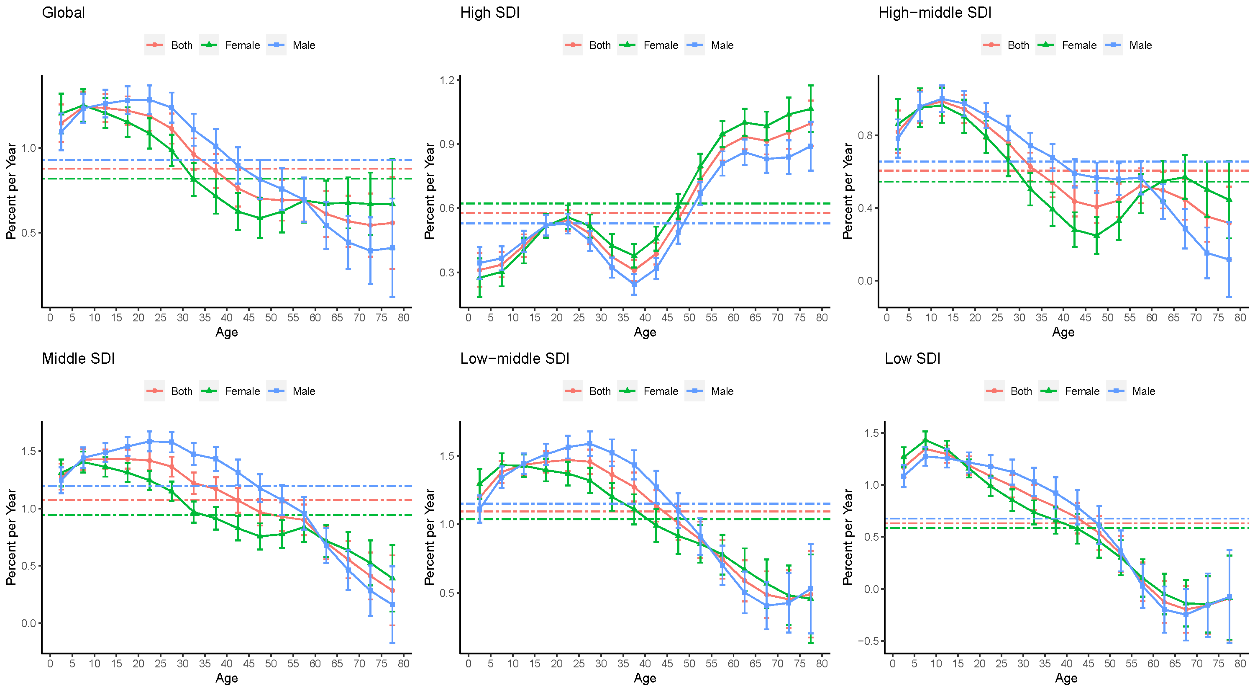
**

**Figure S13. Age, period and cohort effects on epilepsy prevalence for representative countries.**

**
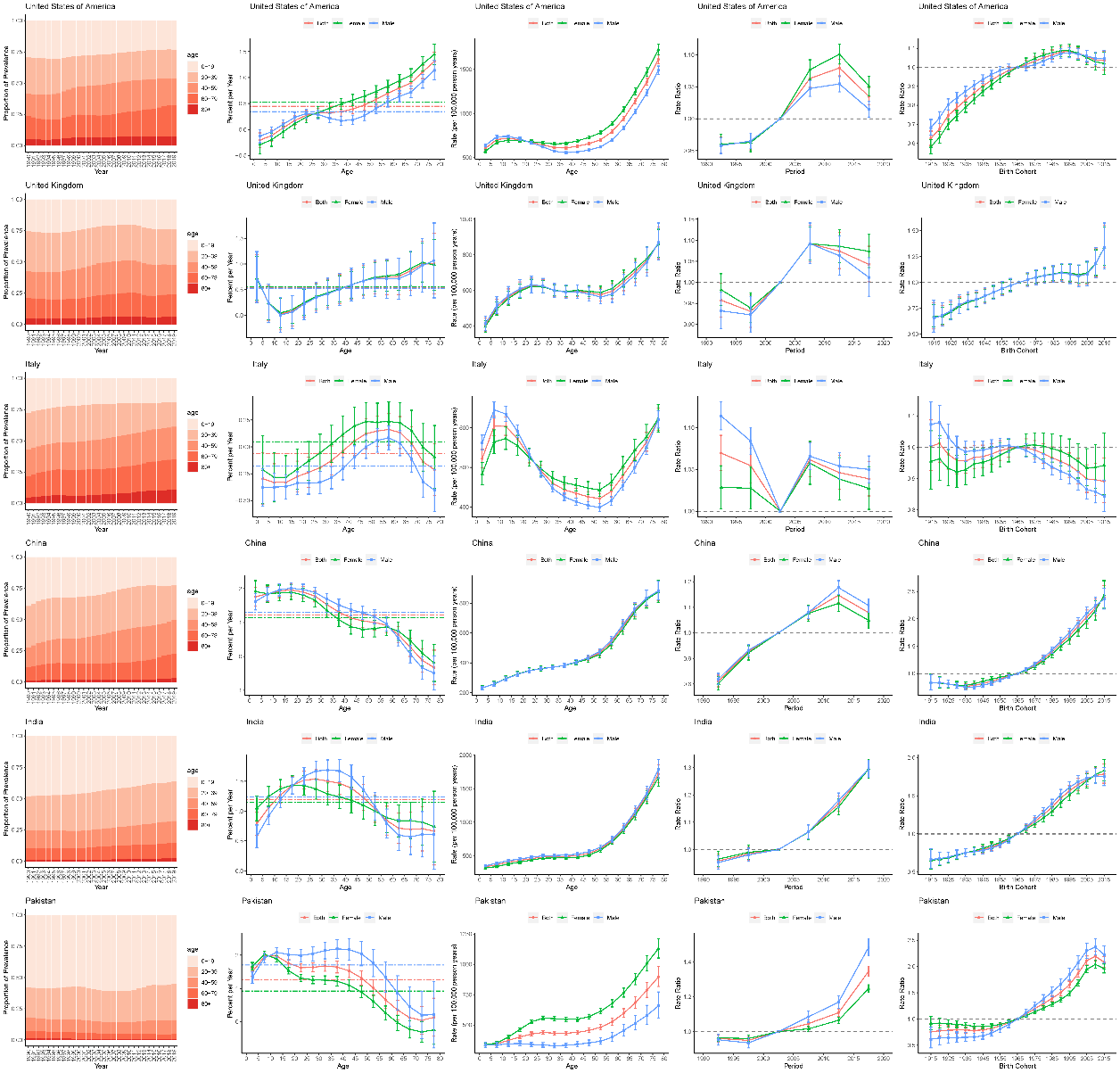
**

**Table S1. Trends in epilepsy Prevalence for both genders across SDI quintiles, 1990−2019.**

|  | **Global** | | **High SDI** | | **High-middle SDI** | | **Middle SDI** | | **Low-middle SDI** | | **Low SDI** | |
| --- | --- | --- | --- | --- | --- | --- | --- | --- | --- | --- | --- | --- |
|  | **1990** | **2019** | **1990** | **2019** | **1990** | **2019** | **1990** | **2019** | **1990** | **2019** | **1990** | **2019** |
| Population | | | | | | | | | | | | |
| Number, n ×1,000,000 | 5350 (5239,5460) | 7737 (7483,7993) | 822 | 1013 | 1150 | 1430 | 1717 | 2397 | 1130 | 1764 | 528 | 1128 |
| Percentage of global, % | 100 | 100 | 15.40 | 13.10 | 21.50 | 18.50 | 32.10 | 31.00 | 21.10 | 22.80 | 9.90 | 14.60 |
| Prevalence | | | | | | | | | | | | |
| Number, n× 1,000 | 27806.79(23324.55,32665.67) | 52543.74(45034.55,60494.58) | 4744.82(3855.11,5640.24) | 6516.78(5256.51,7739.63) | 6004.61(5105.39,6978.93) | 9051.27(7523.73,10728.74) | 8238.75(6773.34,9944.64) | 16103.91(13720.07,18825.34) | 5866.47(4595.04,7169.53) | 12799.75(10698.87,14986.74) | 2935.18(2163.7,3813.22) | 8041.43(6597.98,9643.34) |
| Percentage of global, % | 100 | 100 | 17.06 | 12.40 | 21.59 | 17.23 | 29.63 | 30.65 | 21.10 | 24.36 | 10.56 | 15.30 |
| Percent change of Prevalence 1990–2019, % | 88.96(71.64,108.55) |  | 37.34(21.68,53.01) |  | 50.74(33.67,68.96) |  | 95.47(73.11,124.15) |  | 118.18(86.67,164.46) |  | 173.97(128.75,237) |  |
| All-age Prevalence rate | | | | | | | | | | | | |
| Rate per 100,000 | 519.77(435.99,610.59) | 679.08(582.03,781.84) | 577.22(468.98,686.15) | 643.07(518.71,763.74) | 521.94(443.78,606.64) | 632.78(525.99,750.05) | 479.9(394.54,579.27) | 671.96(572.49,785.51) | 519.32(406.77,634.67) | 725.62(606.52,849.6) | 555.76(409.68,722.01) | 712.47(584.58,854.39) |
| Percent change of rate 1990–2019, % | 30.65(18.67,44.19) |  | 11.41(-1.30,24.12) |  | 21.23(7.51,35.89) |  | 40.02(24.01,60.57) |  | 39.72(19.54,69.36) |  | 28.20(7.04,57.69) |  |
| Age-standardized Prevalence rate | | | | | | | | | | | | |
| Rate per 100,000 | 526.70(443.11,615.02) | 682.67(586.2,789.36) | 575.98(470.06,686.95) | 627.30(510.29,738.88) | 526.21(447.48,611.95) | 636.64(530.91,757.20) | 489.97(405.96,584.64) | 683.73(582.69,798.85) | 541.83(431.69,651.64) | 733.89(618.49,853.71) | 592.63(442.84,755.70) | 718.39(595.68,849.42) |
| Percent change of rate 1990–2019, % | 29.61(18.82,41.80) |  | 8.91(-2.84,20.740) |  | 20.98(7.59,34.97) |  | 39.55(24.44,58.86) |  | 35.45(16.64,61.69) |  | 21.22(2.10,47.98) |  |
| APC model estimates | | | | | | | | | | | | |
| Net drift of Prevalence, % per year | 0.88(0.83,0.93) |  | 0.58(0.55,0.60) |  | 0.61(0.56,0.65) |  | 1.07(1.02,1.13) |  | 1.09(1.04,1.15) |  | 0.63(0.56,0.70) |  |

**Table S2. Trends in epilepsy YLDs for Male and Female genders across SDI quintiles, 1990−2019.**

|  | **Global** | | **High SDI** | | **High-middle SDI** | | **Middle SDI** | | **Low-middle SDI** | | **Low SDI** | |
| --- | --- | --- | --- | --- | --- | --- | --- | --- | --- | --- | --- | --- |
|  | **1990** | **2019** | **1990** | **2019** | **1990** | **2019** | **1990** | **2019** | **1990** | **2019** | **1990** | **2019** |
| **Male** | | | | | | | | | | | | |
| Number, n× 1,000 | 5112.10(3338.63,7215.77) | 9344.94(6260.28,12700.96) | 741.2(471.56,1038.78) | 968.38(618.76,1389.19) | 1041.06(688.13,1423.31) | 1491.31(988.91,2026.03) | 1544.28(998.37,2161.14) | 2844.52(1901.73,3859.81) | 1177.23(723.33,1688.10) | 2432.12(1610.06,3320.77) | 605.23(355.86,925.10) | 1603.11(1047.43,2279.88) |
| Percentage of global, % | 100 | 100 | 14.50 | 10.36 | 20.36 | 15.96 | 30.21 | 30.44 | 23.03 | 26.03 | 11.84 | 17.15 |
| Percent change of YLDs 1990–2019, % | 82.8(63.28,106.81) |  | 30.65(13.61,48.67) |  | 43.25(23.48,64.94) |  | 84.20(59.22,115.5) |  | 106.6(70.68,156.78) |  | 164.87(115.08,241.46) |  |
| All-age YLDs rate | | | | | | | | | | | | |
| Rate per 100,000 | 189.78(123.94,267.87) | 240.79(161.31,327.26) | 183.08(116.48,256.59) | 191.49(122.36,274.7) | 181.98(120.29,248.80) | 208.72(138.4,283.56) | 176.93(114.38,247.60) | 235.89(157.70,320.08) | 204.31(125.54,292.98) | 274.36(181.62,374.60) | 227.19(133.58,347.26) | 283.15(185.01,402.69) |
| Percent change of rate 1990–2019, % | 26.88(13.33,43.54) |  | 4.59(-9.05,19.02) |  | 14.69(-1.13,32.06) |  | 33.32(15.24,55.98) |  | 34.28(10.94,66.90) |  | 24.63(1.21,60.67) |  |
| Age-standardized YLDs rate | | | | | | | | | | | | |
| Rate per 100,000 | 189.52(123.84,265.61) | 243.23(162.66,330.25) | 185.77(118.83,258.39) | 194.71(125.45,276.12) | 183.75(121.07,250.54) | 214.93(141.78,292.82) | 176.87(114.93,246.97) | 240.69(160.3,326.18) | 205.28(126.57,293.98) | 273.97(182.12,373.65) | 231.59(138.52,353.28) | 277.41(178.5,390.73) |
| Percent change of rate 1990–2019, % | 28.34(15.34,44.22) |  | 4.81(-8.11,18.51) |  | 16.97(1.27,34.53) |  | 36.08(17.54,58.94) |  | 33.46(10.86,65.55) |  | 19.78(-2.46,52.83) |  |
| APC model estimates | | | | | | | | | | | | |
| Net drift of YLDs, % per year | 0.84(0.79,0.89) |  | 0.43(0.41,0.45) |  | 0.50(0.46,0.54) |  | 1.03(0.97,1.08) |  | 1.04(0.99,1.09) |  | 0.59(0.51,0.66) |  |
| **Female** | | | | | | | | | | | | |
| Number, n× 1,000 | 4962.43(3256.73,6946.34) | 8960.45(6029.86,12138.65) | 762.5(486.95,1052.47) | 983.69(633.27,1407.14) | 1051.62(696.53,1430.76) | 1470.67(965.59,2006.94) | 1508.74(979.94,2114.69) | 2738.99(1820.71,3743.96) | 1071.47(667.96,1528.62) | 2250.39(1491.55,3045.27) | 564.98(339.7,874.19) | 1511.34(994.39,2139.06) |
| Percentage of global, % | 100 | 100 | 15.37 | 10.98 | 21.19 | 16.41 | 30.40 | 30.57 | 21.59 | 25.11 | 11.39 | 16.87 |
| Percent change of YLDs 1990–2019, % | 80.57(61.91,102.79) |  | 29.01(13.56,45.26) |  | 39.85(22.09,60.11) |  | 81.54(56.95,111.10) |  | 110.03(74.42,160.91) |  | 167.5(117.05,240.84) |  |
| All-age YLDs rate | | | | | | | | | | | | |
| Rate per 100,000 | 186.83(122.61,261.52) | 232.35(156.36,314.76) | 182.78(116.73,252.29) | 193.76(124.74,277.17) | 181.83(120.43,247.38) | 205.43(134.88,280.34) | 178.78(116.12,250.58) | 230.04(152.91,314.44) | 193.59(120.69,276.19) | 256.45(169.98,347.04) | 215.86(129.79,333.99) | 268.68(176.78,380.27) |
| Percent change of rate 1990–2019, % | 24.36(11.51,39.67) |  | 6.01(-6.68,19.36) |  | 12.98(-1.36,29.35) |  | 28.67(11.24,49.62) |  | 32.47(10.01,64.56) |  | 24.47(0.99,58.59) |  |
| Age-standardized YLDs rate | | | | | | | | | | | | |
| Rate per 100,000 | 186.47(122.61,258.68) | 233.88(156.34,316.82) | 182.76(116.97,252.67) | 191.73(123.80,271.04) | 182.21(120.77,246.75) | 208.45(137.29,284.60) | 179.2(117.19,249.75) | 233.84(154.71,318.91) | 197.35(123.43,280.78) | 257.18(170.28,351.04) | 224.29(137.12,344.5) | 266.07(173.51,377.9) |
| Percent change of rate 1990–2019, % | 25.43(13.27,39.85) |  | 4.91(-6.79,17.25) |  | 14.4(0.13,30.11) |  | 30.49(12.79,50.89) |  | 30.32(8.27,61.41) |  | 18.63(-4.04,51.22) |  |
| APC model estimates | | | | | | | | | | | | |
| Net drift of YLDs, % per year | 0.75(0.7,0.8) |  | 0.49(0.46,0.51) |  | 0.4(0.36,0.45) |  | 0.82(0.77,0.87) |  | 0.97(0.91,1.02) |  | 0.53(0.47,0.59) |  |

**Table S3. Trends in epilepsy Prevalence for Male and Female genders across SDI quintiles, 1990−2019.**

|  | **Global** | | **High SDI** | | **High-middle SDI** | | **Middle SDI** | | **Low-middle SDI** | | **Low SDI** | |
| --- | --- | --- | --- | --- | --- | --- | --- | --- | --- | --- | --- | --- |
|  | **1990** | **2019** | **1990** | **2019** | **1990** | **2019** | **1990** | **2019** | **1990** | **2019** | **1990** | **2019** |
| **Male** | | | | | | | | | | | | |
| Number, n× 1,000 | 14016.75(11716.27,16525.35) | 26683.48(22947.01,30742.25) | 2333.95(1898.41,2780.47) | 3225.17(2604.87,3838.09) | 2975.32(2513.69,3460.29) | 4542.92(3784.61,5366.17) | 4145.71(3388.68,5023.5) | 8172.29(6978.69,9577.56) | 3047.59(2373.29,3739.86) | 6607.14(5509.82,7747.56) | 1505.76(1100.43,1960.01) | 4120.54(3371.87,4943.45) |
| Percentage of global, % | 100 | 100 | 16.65 | 12.09 | 21.23 | 17.03 | 29.58 | 30.63 | 21.74 | 24.76 | 10.74 | 15.44 |
| Percent change of Prevalence 1990–2019, % | 90.37(72.42,110.99) |  | 38.19(21.44,55.11) |  | 52.69(34.89,72.37) |  | 97.13(73.83,127.26) |  | 116.8(83.89,161.64) |  | 173.65(127.83,237.33) |  |
| All-age Prevalence rate | | | | | | | | | | | | |
| Rate per 100,000 | 520.34(434.94,613.47) | 687.55(591.27,792.13) | 576.51(468.93,686.81) | 637.75(515.09,758.95) | 520.1(439.41,604.88) | 635.81(529.68,751.03) | 474.97(388.24,575.54) | 677.7(578.72,794.23) | 528.92(411.89,649.07) | 745.32(621.54,873.97) | 565.22(413.07,735.73) | 727.8(595.56,873.15) |
| Percent change of rate 1990–2019, % | 32.13(19.68,46.44) |  | 10.62(-2.79,24.17) |  | 22.25(8,38.01) |  | 42.68(25.82,64.49) |  | 40.91(19.53,70.06) |  | 28.76(7.2,58.72) |  |
| Age-standardized Prevalence rate | | | | | | | | | | | | |
| Rate per 100,000 | 529.67(444.9,620.6) | 694.71(597.31,802.04) | 582.7(474.46,696.04) | 633.31(513.52,749.83) | 529.58(447.95,616.33) | 647.26(539.2,768.27) | 485.18(400.57,580.29) | 692.2(591.88,809.47) | 548.06(435.85,659.76) | 753.43(635.08,877.44) | 596.8(444.82,766.12) | 730.24(602.69,863.65) |
| Percent change of rate 1990–2019, % | 31.16(19.92,44.2) |  | 8.69(-3.68,21.6) |  | 22.22(8.37,37.07) |  | 42.67(26.76,62.93) |  | 37.47(17.79,64.88) |  | 22.36(3.08,49.19) |  |
| APC model estimates | | | | | | | | | | | | |
| Net drift of Prevalence, % per year | 0.93(0.88,0.98) |  | 0.53(0.5,0.55) |  | 0.66(0.62,0.69) |  | 1.2(1.14,1.25) |  | 1.15(1.1,1.21) |  | 0.68(0.6,0.76) |  |
| **Female** | | | | | | | | | | | | |
| Number, n× 1,000 | 13790.03(11579.2,16147.13) | 25860.26(22096.84,29695.01) | 2410.88(1956.89,2866.24) | 3291.61(2679.26,3894.61) | 3029.28(2575.61,3515.62) | 4508.34(3749.02,5348.86) | 4093.04(3385.76,4921.39) | 7931.62(6715.17,9268.7) | 2818.88(2212.54,3419.89) | 6192.61(5204.3,7239.35) | 1429.42(1059.89,1850.14) | 3920.89(3208.89,4694.7) |
| Percentage of global, % | 100 | 100 | 17.48 | 12.73 | 21.97 | 17.43 | 29.68 | 30.67 | 20.44 | 23.95 | 10.37 | 15.16 |
| Percent change of Prevalence 1990–2019, % | 87.53(70.65,105.69) |  | 36.53(21.69,51.43) |  | 48.83(32.39,66.36) |  | 93.78(71.29,121.38) |  | 119.68(88.11,165.52) |  | 174.3(128.91,236.66) |  |
| All-age Prevalence rate | | | | | | | | | | | | |
| Rate per 100,000 | 519.18(435.95,607.93) | 670.56(572.98,770) | 577.91(469.09,687.06) | 648.37(527.75,767.15) | 523.77(445.32,607.85) | 629.75(523.68,747.15) | 485(401.19,583.15) | 666.14(563.98,778.44) | 509.32(399.77,617.91) | 705.71(593.08,824.99) | 546.12(404.94,706.87) | 697.03(570.46,834.59) |
| Percent change of rate 1990–2019, % | 29.16(17.53,41.66) |  | 12.19(0,24.43) |  | 20.23(6.96,34.4) |  | 37.35(21.41,56.91) |  | 38.56(18.64,67.47) |  | 27.63(6.51,56.65) |  |
| Age-standardized Prevalence rate | | | | | | | | | | | | |
| Rate per 100,000 | 524.48(443.04,609.14) | 670.29(571.99,771.7) | 569.37(464.11,678.91) | 621.2(507.27,730.31) | 523.78(445.85,609.75) | 625.6(521.01,747.38) | 495.45(413.23,588.41) | 674.56(570.9,791.16) | 535.4(427.41,644.14) | 713.87(603.43,830.28) | 589.21(442.9,747.63) | 707.48(586.58,836.99) |
| Percent change of rate 1990–2019, % | 27.8(17.3,39.29) |  | 9.1(-2.21,20.57) |  | 19.44(6.79,32.75) |  | 36.15(21.48,54.09) |  | 33.34(14.93,59.91) |  | 20.07(0.93,46.09) |  |
| APC model estimates | | | | | | | | | | | | |
| Net drift of Prevalence, % per year | 0.82(0.77,0.87) |  | 0.62(0.6,0.65) |  | 0.55(0.5,0.59) |  | 0.94(0.89,1) |  | 1.04(0.98,1.09) |  | 0.59(0.52,0.65) |  |

**Table S4. Trends in epilepsy YLDs for both genders in 204 countries and regions, 1990-2019.**

| **SDI quintile** | **Country** | **Deaths** | | **All-age YLDs** | | **Age-standardized YLDs** | | **Net drift of YLDs from APC model,% per year** |
| --- | --- | --- | --- | --- | --- | --- | --- | --- |
|  |  | **Number in 2019** | **Percent change 1990-2019, %** | **Rate in 2019** | **Percent change 1990-2019, %** | **Rate in 2019** | **Percent change 1990-2019, %** |  |
| High SDI | Denmark | 9053.18(4535.94,16330.46) | 19.55(-40.09,154.62) | 156.02(78.17,281.43) | 5.98(-46.89,125.73) | 155.52(81.36,269.9) | 3.43(-45.8,110.7) | 0.45(0.34,0.56) |
|  | Puerto Rico | 12431.39(7109.79,19756.89) | -7.77(-44.86,56.03) | 353.02(201.9,561.05) | -5.36(-43.42,60.1) | 340.92(197.81,531.01) | -8.27(-43.87,53.16) | -0.4(-0.49,-0.31) |
|  | Switzerland | 14401.07(7599.02,25503.21) | 26.34(-34.47,161.23) | 164.11(86.6,290.63) | -1.15(-48.73,104.38) | 163.32(90.07,279.43) | -1.56(-45.08,94.25) | -0.03(-0.12,0.06) |
|  | Russian Federation | 270999.58(179002.97,372769.52) | -4.7(-15.56,7.15) | 184.71(122.01,254.07) | -1.91(-13.08,10.3) | 185.92(123.54,255.43) | -1.81(-12.45,10.39) | -0.16(-0.21,-0.12) |
|  | Germany | 214474.72(97385.66,416540.47) | 38.06(-39.75,199.32) | 252.58(114.69,490.54) | 29.97(-43.28,181.8) | 251.58(122.68,475.82) | 26.64(-39.89,162.04) | 1.2(1.16,1.25) |
|  | Finland | 8547.08(3855.8,15992.4) | 6.67(-50.27,128.69) | 154.44(69.67,288.98) | -3.43(-54.98,107.04) | 155.74(73.7,282.42) | -2.26(-51.59,98.16) | -0.03(-0.14,0.07) |
|  | France | 116836.81(51001.4,227661.04) | 18.63(-47.98,187.79) | 176.48(77.04,343.88) | 3.52(-54.61,151.12) | 168.21(80.9,321.24) | -0.67(-53.9,130.85) | -0.12(-0.18,-0.06) |
|  | Czechia | 25712.71(12570.94,46625.21) | 16.97(-47.97,154.48) | 241.58(118.11,438.06) | 13.17(-49.66,146.21) | 239.82(125.56,423.44) | 11.72(-46.93,131.12) | 0.62(0.55,0.68) |
|  | United Kingdom | 122616.24(78102.31,175024.05) | 17.28(4.05,31.16) | 182.41(116.19,260.37) | 0.27(-11.04,12.14) | 187.67(120.79,268.5) | 1.99(-9.46,13.45) | 0.38(0.26,0.51) |
|  | San Marino | 56.54(30.97,94.49) | 39.54(-18.28,144.06) | 170.83(93.57,285.46) | -0.7(-41.84,73.69) | 167.41(93.26,277.47) | -0.98(-41.9,71.95) | 0.02(-1.42,1.47) |
|  | Brunei Darussalam | 846.16(461.46,1448.27) | 44.93(-23,211.36) | 193.58(105.57,331.32) | -14.28(-54.46,84.16) | 198.42(106.3,344.03) | -11.68(-54.54,96.99) | -0.3(-0.78,0.18) |
|  | Kuwait | 10661.74(6107.94,17176.94) | 142.81(50.36,317.54) | 240.86(137.98,388.04) | -3.49(-40.24,65.96) | 250.99(142.04,408.83) | 3.58(-39,88.15) | 0.11(-0.07,0.29) |
|  | Ireland | 8066.3(3829.85,14896.19) | 41.44(-34.31,204.96) | 164.27(78,303.36) | 3.73(-51.82,123.66) | 164.71(79.76,298.14) | 4.39(-50.14,120.63) | 0.1(-0.02,0.23) |
|  | Republic of Korea | 83999.93(49603.02,133134.18) | 9.9(-36.32,104.57) | 157.31(92.89,249.32) | -8.75(-47.12,69.86) | 158.18(94.88,253.2) | -9.2(-47.65,68.22) | -0.38(-0.42,-0.34) |
|  | Taiwan (Province of China) | 42397.26(22972.28,70219.4) | 45.62(-20.17,150.07) | 179.5(97.26,297.28) | 25.76(-31.06,115.96) | 171.1(94.68,277.96) | 17.08(-34.18,103.41) | 0.85(0.79,0.91) |
|  | Luxembourg | 1278.84(644.33,2280.89) | 73.22(-14.07,270.86) | 206.75(104.17,368.75) | 6.77(-47.04,128.59) | 202.69(105.52,352.76) | 4.66(-45.81,114.4) | 0.31(-0.01,0.63) |
|  | Singapore | 8229.15(4874.75,12879.35) | 96.38(23.62,232.41) | 145.2(86.01,227.25) | 5.59(-33.53,78.73) | 155.36(92.37,244.18) | 12.32(-30.3,92.92) | 0.6(0.46,0.74) |
|  | Slovenia | 4303.01(2368.35,7631.21) | 2.26(-46.33,129.88) | 207.45(114.18,367.9) | -2.83(-49,118.45) | 209.8(121,355.22) | -1.07(-45.36,109.36) | -0.1(-0.25,0.04) |
|  | United States of America | 706907.22(469098.76,981842.64) | 28.01(12.61,43.65) | 215.53(143.03,299.36) | -1.02(-12.93,11.07) | 210.77(139.09,291.81) | -3.23(-14.37,8.58) | 0.42(0.37,0.46) |
|  | Australia | 38934.5(20511.59,66528.97) | 28.61(-28.52,130) | 158.48(83.49,270.79) | -11.74(-50.94,57.84) | 161.78(88.04,271.91) | -10.56(-48.44,56.2) | -0.38(-0.43,-0.33) |
|  | Iceland | 544.73(276.03,959.51) | 45.44(-24.58,192.15) | 157.95(80.04,278.22) | 7.1(-44.46,115.14) | 158.09(82.02,273.4) | 7.3(-43.39,112.68) | 0.37(-0.12,0.85) |
|  | Canada | 50770.54(29331.32,82654.09) | 33.44(-22.9,126.15) | 139.02(80.32,226.33) | -0.41(-42.45,68.79) | 143.33(84.4,228.38) | 1.97(-37.8,67.85) | 0.12(0.07,0.16) |
|  | Japan | 159948.64(101364.11,229351.93) | 2.54(-13.91,22.44) | 125.17(79.32,179.48) | 1(-15.2,20.59) | 135.3(87.58,191.61) | 5.61(-9.66,23.74) | 0.26(0.21,0.32) |
|  | Slovakia | 14856.13(7555.73,26470.53) | 12.66(-41.88,118.06) | 273.23(138.96,486.84) | 9.46(-43.53,111.87) | 251.82(128.52,446.95) | 2.72(-47,98.97) | 0.11(0.03,0.2) |
|  | New Zealand | 7538.99(4310.14,12156.59) | 30.71(-24.57,119.5) | 167.69(95.87,270.41) | -0.64(-42.67,66.85) | 175.45(102.7,279.39) | 3.22(-38.96,71.5) | 0.21(0.09,0.34) |
|  | Estonia | 3397.5(1795.89,5780.82) | -12.06(-53,73.3) | 258.88(136.84,440.49) | 5.12(-43.81,107.15) | 236.6(127.99,401.66) | -1.99(-46.29,88.45) | 0.17(0.01,0.34) |
|  | Belgium | 22407.51(10552.75,41606.06) | 25.45(-40.84,195.65) | 196.23(92.41,364.35) | 9.63(-48.3,158.38) | 189.93(95.77,333.59) | 5.3(-46.46,135.65) | 0.39(0.32,0.46) |
|  | Qatar | 6833.11(4035.4,11003.6) | 504.81(265.91,975.33) | 238.54(140.87,384.13) | -6.02(-43.14,67.1) | 251.53(144.14,411.41) | -1.38(-42.92,85.8) | 0.04(-0.34,0.41) |
|  | Saudi Arabia | 98870.91(50111.14,161922.67) | 155.08(23.81,399.98) | 276.7(140.24,453.16) | 14.54(-44.4,124.51) | 282.17(140.28,469.03) | 21.06(-41.83,147.29) | 0.85(0.79,0.9) |
|  | Latvia | 4537.49(2540.59,7253.61) | -26.86(-58.74,27.9) | 236.91(132.65,378.72) | 1.53(-42.72,77.54) | 226.21(127.55,352.18) | -1.66(-43.01,68.78) | 0.09(-0.04,0.22) |
|  | Austria | 17832.18(9910.39,29142.67) | 19.16(-31.59,114.46) | 200(111.15,326.85) | 3.82(-40.4,86.86) | 191.88(107.75,310.79) | 1.61(-41.79,81.96) | 0.01(-0.07,0.09) |
|  | Andorra | 127.23(66.55,220.87) | 43.6(-26.03,187.35) | 153.17(80.12,265.91) | -6.48(-51.83,87.13) | 156.24(83.74,266.55) | -4.87(-49.79,89.31) | -0.17(-1.11,0.77) |
|  | Netherlands | 30402.18(15362,53818.49) | 24.31(-35.28,148.24) | 177.2(89.54,313.69) | 8.13(-43.7,115.93) | 177.26(94.42,304.19) | 10.65(-39.16,111.1) | 0.35(0.24,0.46) |
|  | United Arab Emirates | 23854.48(12792.7,38098.64) | 335.95(122.73,792.15) | 258.12(138.42,412.25) | -11.69(-54.88,80.72) | 280.54(143.62,456.06) | -7.71(-54.18,106.48) | -0.49(-0.65,-0.33) |
|  | Lithuania | 6810.44(3472.98,11324.05) | -22(-58.15,43.93) | 243.73(124.29,405.27) | 2.55(-44.99,89.23) | 225.01(117.59,366.83) | -3(-46.48,79.94) | 0.08(-0.03,0.19) |
|  | Bermuda | 187.62(105.09,317.65) | -6.03(-49.08,78.49) | 293.02(164.12,496.1) | -12.75(-52.72,65.73) | 272.86(152.96,455.09) | -18.13(-55.26,54.44) | -0.79(-1.49,-0.08) |
|  | Cyprus | 2068.79(1095.09,3570.1) | 58.79(-16.45,204.69) | 157.5(83.37,271.8) | -5.95(-50.51,80.48) | 161.13(87.02,276.2) | -4.25(-48.69,81.18) | -0.08(-0.33,0.17) |
|  | Guam | 381.96(207.7,607.08) | 37.23(-20.96,136.13) | 223.86(121.73,355.79) | 9.99(-36.65,89.26) | 226.04(123.7,359.82) | 11.31(-35.73,91.14) | 0.45(-0.16,1.07) |
|  | Norway | 9603(5881.62,14423.14) | 22.48(-1.55,52.51) | 179.53(109.96,269.65) | -2.76(-21.83,21.09) | 179.77(111,268.3) | -3.07(-21.19,20.32) | -0.06(-0.16,0.05) |
|  | Sweden | 12332.8(6954.65,20329.09) | 11.49(-35.9,97.06) | 120.64(68.03,198.87) | -6.33(-46.15,65.57) | 123.11(72.46,199.06) | -7.24(-44.82,56.2) | -0.19(-0.28,-0.1) |
|  | Monaco | 80.59(41.1,137.96) | 35.5(-27.81,139.87) | 214.48(109.38,367.18) | 9.78(-41.51,94.34) | 194.84(103.49,331.09) | 7.74(-40.4,90.24) | 0.37(-0.9,1.66) |
| High middle SDI | Dominica | 321.85(179.9,506.65) | 3.87(-42.05,95.38) | 468.62(261.93,737.69) | 11.92(-37.56,110.53) | 461.05(260.89,722.06) | 9.47(-38.66,104.46) | 0.16(-0.43,0.75) |
|  | Croatia | 12666.83(7304.84,21058.71) | -16.48(-53.7,57.28) | 298.19(171.96,495.74) | -3.65(-46.59,81.45) | 271.59(159.3,445.5) | -8.21(-48.63,72.24) | -0.27(-0.38,-0.16) |
|  | Ukraine | 85127.29(49708.65,137792.97) | -27.65(-57.62,20.41) | 193.28(112.87,312.86) | -13.49(-49.33,43.97) | 196.56(115.3,314.4) | -12.6(-48.19,43.84) | -0.53(-0.56,-0.49) |
|  | Hungary | 25755.62(14553.12,41356.92) | -14.93(-50.89,49.35) | 266.22(150.43,427.49) | -8.61(-47.25,60.44) | 253.75(146.32,402.78) | -11.48(-48.2,53.47) | -0.48(-0.54,-0.42) |
|  | Argentina | 80332.79(46362.69,127670.63) | 36.51(-18.42,131.01) | 178.06(102.76,282.99) | 0.21(-40.11,69.59) | 179.13(102.7,286.14) | 0.88(-39.59,70.7) | -0.02(-0.08,0.04) |
|  | Saint Kitts and Nevis | 214.42(117.54,346.7) | 32.1(-29.62,149.71) | 360.31(197.51,582.62) | -8.2(-51.1,73.52) | 355.94(196.2,573.16) | -11.42(-52.24,68.1) | -0.76(-1.51,0) |
|  | North Macedonia | 5359.86(3100.53,8453.37) | 12.91(-34.49,103.62) | 248.98(144.03,392.68) | 5.71(-38.67,90.64) | 246.8(145.82,389.22) | 4.22(-39.04,88.56) | 0.12(-0.02,0.26) |
|  | Kazakhstan | 48611.13(24715.25,81054.15) | 16.58(-40.95,155.92) | 264.3(134.38,440.7) | 3.75(-47.45,127.75) | 266.68(135.47,447.13) | 4.33(-46.55,130.57) | 0.15(0.1,0.2) |
|  | Romania | 51217.06(28699.98,84472.64) | -16.69(-53.27,62.03) | 266.24(149.19,439.11) | 1.32(-43.16,97.06) | 251.93(143.28,416.95) | -2.74(-45.07,89.86) | -0.1(-0.15,-0.06) |
|  | Chile | 45364.05(24778.23,75227.85) | 36.25(-29.06,186.1) | 249.28(136.16,413.38) | -0.57(-48.23,108.78) | 246.2(132.75,408.69) | -2.02(-49.24,105.47) | 0.09(0.04,0.14) |
|  | Poland | 98870.36(63099.85,140147.32) | 24.38(-0.4,52.12) | 257.24(164.18,364.64) | 23.48(-1.11,51.02) | 229.57(147.49,326.5) | 11.28(-9.45,35.86) | 0.46(0.42,0.49) |
|  | Montenegro | 1534.74(872.95,2517.7) | -4.58(-46.43,57.42) | 247.4(140.72,405.86) | -3.75(-45.97,58.78) | 244.61(141.51,399.57) | -4.92(-45.89,55.84) | -0.13(-0.38,0.13) |
|  | Greece | 18962.28(10667.48,31670.36) | 12.41(-36.12,100.8) | 183.44(103.2,306.37) | 12.98(-35.8,101.82) | 180.64(102.96,296.06) | 13.01(-34.32,103.41) | 0.6(0.52,0.67) |
|  | Malaysia | 69035.2(38188.34,112755.83) | 73.2(-0.32,205) | 220.55(122,360.23) | -2.31(-43.78,72.03) | 222.92(121.97,365.74) | 1.74(-42.62,84.75) | 0.1(0.05,0.15) |
|  | United States Virgin Islands | 351.16(187.11,558.23) | 8.35(-40.69,91.72) | 337.7(179.94,536.83) | 10.46(-39.54,95.46) | 326.61(178.07,513.11) | 5.3(-42.2,87.36) | 0.13(-0.41,0.67) |
|  | Lebanon | 11463.57(6530.5,18472.2) | 55.2(-11.22,189.96) | 221.43(126.14,356.81) | -1.82(-43.83,83.43) | 224.02(127.79,360.21) | 3.76(-41.67,94.9) | 0.16(0.03,0.29) |
|  | Serbia | 22891(12785.11,38008.59) | -7.21(-46.91,79.95) | 261.71(146.17,434.54) | -0.31(-42.96,93.34) | 240.34(135.15,392.7) | -7.74(-46.35,79.02) | -0.26(-0.32,-0.19) |
|  | Antigua and Barbuda | 276.73(128.49,490.49) | 35.56(-40.05,214.32) | 312.73(145.21,554.3) | -6.98(-58.86,115.7) | 310.62(147.18,543.29) | -6.67(-58.42,117.71) | -0.44(-1.12,0.25) |
|  | Israel | 17674.47(9853.55,29693.9) | 89.98(7.78,230.48) | 189.85(105.84,318.96) | 1.26(-42.55,76.14) | 187.31(104.35,316.59) | 0.23(-42.71,73.77) | 0.2(0.1,0.29) |
|  | Bulgaria | 14929.67(7220.64,26646.03) | -12.57(-56.75,99.72) | 215.29(104.12,384.25) | 9.45(-45.86,150.02) | 221.77(113.18,382.77) | 10.31(-42.17,141.03) | 0.47(0.39,0.55) |
|  | Italy | 88726.93(56264.41,129422.28) | 3.88(-14.16,28.81) | 147.11(93.29,214.58) | -2.18(-19.16,21.31) | 149.58(96.12,214.34) | -3.72(-19.45,16.36) | -0.13(-0.19,-0.08) |
|  | Barbados | 1202.69(700.6,1847.86) | 20.12(-26.77,108.27) | 403.9(235.28,620.56) | 2.42(-37.56,77.58) | 397.71(233.91,605.08) | 2.93(-36.39,75.34) | 0.02(-0.28,0.33) |
|  | Trinidad and Tobago | 5194.79(2868.11,8236.41) | 17.76(-36.97,133.07) | 374.41(206.72,593.63) | 2.11(-45.35,102.08) | 365.08(204.56,577.77) | -2.5(-48.1,91.58) | 0(-0.15,0.15) |
|  | Bahamas | 1272.27(683.49,2018.37) | 39.04(-21.36,155.35) | 337.53(181.33,535.46) | -5.4(-46.49,73.74) | 336.74(181.85,530.75) | -7.79(-46.79,71.85) | -0.34(-0.66,-0.02) |
|  | Bosnia and Herzegovina | 9155.47(5135.7,14694.93) | -28.34(-59.84,22.68) | 277.44(155.63,445.3) | -1.45(-44.77,68.71) | 271.04(148.55,426.7) | -3.86(-45.85,65.66) | 0.05(-0.04,0.15) |
|  | Malta | 736.23(410.09,1249.31) | 24.89(-28.35,125.35) | 167.62(93.37,284.44) | 5.41(-39.53,90.19) | 174.3(100.23,287.39) | 8.05(-36.75,92.09) | 0.5(0.11,0.89) |
|  | Jordan | 28619.97(16551.9,45033.25) | 215.63(87.75,436.06) | 245.95(142.24,386.99) | 2.34(-39.12,73.81) | 236.81(135.65,375.92) | 8.53(-35.77,87.72) | 0.41(0.27,0.54) |
|  | Belarus | 20341.91(11814.77,32110.93) | -13.03(-46.52,46.98) | 214.11(124.36,337.98) | -4.16(-41.07,61.98) | 212.81(126.13,335.68) | -4.81(-40.5,58.29) | -0.26(-0.33,-0.2) |
|  | Seychelles | 265.67(150.08,416.2) | 48.2(-11.63,160.75) | 260.09(146.93,407.46) | 5.96(-36.82,86.42) | 267.46(153.18,413.48) | 12(-33.14,100.05) | 0.29(-0.49,1.08) |
|  | Portugal | 15070.43(8008.16,26257.61) | 20.2(-34.9,120.72) | 141.49(75.19,246.52) | 14.4(-38.04,110.07) | 140.65(78.91,239.01) | 11.43(-36.26,99.63) | 0.42(0.34,0.5) |
|  | Cook Islands | 37.31(19.98,58.86) | -6.09(-46.72,69.13) | 207.42(111.1,327.24) | -0.68(-43.65,78.86) | 210.37(113.73,329.74) | 2.09(-42.18,88.25) | 0.1(-1.66,1.88) |
|  | Libya | 13562.4(8038.21,20757.87) | 31.52(-22.3,133.05) | 201.36(119.34,308.18) | -17.27(-51.12,46.6) | 203.57(119.42,312.48) | -9.93(-48.07,64.71) | -0.39(-0.51,-0.26) |
|  | American Samoa | 123.21(63.02,204.8) | 13.18(-40.16,109.01) | 221.99(113.54,368.97) | -1.24(-47.78,82.38) | 222.85(113.05,376.53) | -1.06(-48.59,87.5) | 0.11(-0.97,1.21) |
|  | Turkey | 231439.36(126139.2,373867.81) | 55.35(-14.23,221.45) | 284.46(155.04,459.52) | 14.13(-36.99,136.16) | 292.9(158.8,474.1) | 23.64(-31.89,158.35) | 1.1(1.02,1.19) |
|  | Bahrain | 3590.46(2021.71,5791.13) | 153.58(38.74,397.33) | 248.87(140.13,401.41) | -10.7(-51.14,75.14) | 266.75(145.89,440.39) | -7.25(-51.73,98.59) | -0.39(-0.66,-0.12) |
|  | Spain | 78161.98(44382.83,129242.41) | 20.07(-31.08,116.99) | 169.84(96.44,280.83) | 1.18(-41.92,82.85) | 156.76(88.22,257.65) | -5.1(-45.76,68.96) | 0.04(0,0.08) |
|  | Oman | 10141.74(6029.08,15810.01) | 151.87(48.76,349.21) | 221.24(131.52,344.9) | 6.77(-36.94,90.42) | 223.69(130.44,356.79) | 17.36(-33.84,120.52) | 0.76(0.55,0.97) |
|  | Greenland | 129.3(57.56,233.75) | -14.53(-61.75,103.87) | 230.11(102.43,416.01) | -15.46(-62.17,101.65) | 227.6(105.53,402.22) | -17.81(-61.89,96.91) | -0.47(-1.33,0.41) |
|  | Niue | 3.38(1.82,5.44) | -26.82(-60.36,33.54) | 202.5(109.02,325.64) | 1.86(-44.82,85.87) | 204.91(112.53,328.4) | 4.01(-43.18,91.77) | 0.11(-5.34,5.86) |
|  | Northern Mariana Islands | 81.08(45,131.94) | -7.98(-50.95,73.72) | 190.8(105.91,310.48) | -1.7(-47.6,85.59) | 197.6(110.35,321.19) | 0.28(-45.97,89.65) | 0.1(-1.12,1.35) |
|  | Palau | 32.08(17.04,53.29) | 13.65(-37.77,110.06) | 178.17(94.6,295.96) | -2.78(-46.77,79.69) | 188.44(101.37,308.86) | 4.25(-42.07,95.22) | 0.1(-1.87,2.11) |
| Middle SDI | Costa Rica | 14040.58(7202.26,23249.47) | 49.58(-23.67,188.96) | 297.68(152.7,492.91) | -3.6(-50.8,86.23) | 294.7(150.18,494.14) | -7.52(-52.63,77.06) | -0.33(-0.43,-0.23) |
|  | Iran (Islamic Republic of) | 187321.9(122611.24,261177.67) | 76.31(39.5,123.86) | 222.21(145.45,309.83) | 22.44(-3.13,55.46) | 227.15(149.26,321.6) | 38.18(9.77,76.97) | 1.46(1.39,1.53) |
|  | Gabon | 9853.14(5159.14,15901.42) | 104.79(5.12,363.85) | 563.02(294.8,908.63) | 16.03(-40.44,162.81) | 571.43(297.36,921.82) | 11.23(-43.09,152.89) | 0.11(-0.03,0.26) |
|  | Armenia | 7854.47(4635.3,12266.36) | -9.91(-46.47,57.41) | 260.11(153.5,406.21) | 1.87(-39.47,78) | 263.41(154.02,411.74) | 2.91(-39.35,83.3) | 0.16(0.04,0.27) |
|  | Azerbaijan | 27737.52(14577.39,46750.89) | 55.63(-23.69,279.86) | 269.86(141.82,454.83) | 11(-45.57,170.93) | 276.08(143.65,468.83) | 17.72(-42.66,188.39) | 0.97(0.89,1.06) |
|  | Grenada | 390.36(219.06,600.35) | 41.15(-23.08,158.33) | 378.2(212.23,581.65) | 17.16(-36.15,114.43) | 375.05(209.97,577.05) | 14.1(-37.12,106.23) | 0.3(-0.28,0.89) |
|  | Iraq | 96893.27(54889.48,152372.56) | 134.65(35.55,320.92) | 230.04(130.32,361.76) | -1.97(-43.37,75.85) | 219.14(122.9,347.91) | 2.91(-41.9,91.27) | 0.22(0.15,0.28) |
|  | Tokelau | 2.69(1.51,4.34) | -13.09(-52.1,56.84) | 190.6(106.77,307.85) | 3.94(-42.72,87.57) | 190.57(106.4,306.27) | 6.64(-42.03,95.65) | 0.3(-6.92,8.07) |
|  | Sri Lanka | 62440.3(34038.1,101315.56) | 43.68(-22.24,197.51) | 285.71(155.75,463.59) | 13.22(-38.72,134.44) | 286.11(156.41,463.16) | 12.04(-39.64,131.98) | 0.4(0.35,0.44) |
|  | China | 2438179.97(1593120.67,3355791.57) | 76.37(42.14,120.53) | 171.42(112.01,235.93) | 46.78(18.29,83.52) | 176.82(116.35,241.6) | 49.9(22.86,84.19) | 1.04(0.94,1.13) |
|  | Georgia | 10249.88(5879.19,15938.77) | -31.13(-60.04,22.93) | 279.69(160.43,434.92) | 3.52(-39.94,84.78) | 278.93(159.76,434.27) | 2.79(-40.3,86.18) | 0.43(0.34,0.53) |
|  | Botswana | 9079.59(5152.18,13966.78) | 119.75(27.79,287.25) | 388.23(220.3,597.2) | 22.31(-28.87,115.54) | 398.41(223.66,619.96) | 20(-30.78,114.36) | 0.61(0.46,0.77) |
|  | Thailand | 160567.44(88412.65,263482.21) | 57.76(-16.84,195.28) | 229.02(126.1,375.8) | 27.98(-32.54,139.54) | 234.04(131.42,379.66) | 30.75(-30.3,140.2) | 1.25(1.21,1.28) |
|  | Uruguay | 8108.54(4266.59,13400.39) | 17.3(-35.13,121.78) | 235.98(124.17,389.98) | 7.17(-40.74,102.63) | 231.87(121.56,384.03) | 5.44(-41.7,97.67) | 0.36(0.24,0.48) |
|  | South Africa | 196420.83(126503.76,276632.63) | 75.9(39.53,127.4) | 353.35(227.57,497.64) | 16.54(-7.56,50.66) | 360.53(234.12,506.89) | 12.8(-10.26,45.63) | -0.12(-0.22,-0.03) |
|  | Saint Lucia | 708.16(406.18,1133.3) | 36.7(-25.39,162.2) | 405.53(232.6,648.99) | 7.56(-41.3,106.31) | 395.23(226.09,630.31) | -0.61(-45.64,89.16) | -0.25(-0.67,0.18) |
|  | Mexico | 482390.24(313426.63,666118.88) | 50.37(21.35,86.52) | 386.1(250.86,533.15) | 2.89(-16.97,27.62) | 387.99(252.59,535.91) | -3.52(-21.12,17.36) | -0.22(-0.25,-0.19) |
|  | Fiji | 2166.76(1141.48,3594.26) | 26.49(-37.35,141.32) | 237.78(125.27,394.43) | 5.41(-47.8,101.09) | 238.94(125.14,395.47) | 6.69(-48.44,106.65) | 0.14(-0.13,0.42) |
|  | Jamaica | 10724.87(6396.63,16983.56) | 49.99(-9.61,149.25) | 381.57(227.58,604.24) | 26.13(-23.98,109.61) | 379.9(226.26,597.39) | 24.63(-24.28,107.46) | 0.86(0.74,0.98) |
|  | Indonesia | 473781.3(310745.74,672518.16) | 120.51(72.28,199.37) | 182.6(119.76,259.19) | 57.54(23.09,113.89) | 185.25(122.12,262.97) | 67.91(30.34,128.85) | 1.98(1.93,2.03) |
|  | Egypt | 218663.5(126466.28,328715.26) | 101.41(12.29,286.68) | 220.72(127.65,331.8) | 13.23(-36.87,117.39) | 211.68(121.08,319.72) | 18.38(-34.86,133.09) | 1.17(1.05,1.29) |
|  | Paraguay | 19452.78(10524.37,31140.19) | 58.88(-10.04,198.81) | 280.69(151.86,449.32) | -7.26(-47.49,74.42) | 282.83(152.92,453.06) | -8.6(-48.67,71.15) | -0.52(-0.62,-0.43) |
|  | Saint Vincent and the Grenadines | 424.6(246,688.68) | 17.72(-34.43,124.87) | 375.27(217.42,608.68) | 14.51(-36.22,118.74) | 370.1(215.91,599.7) | 10.24(-38.76,111.52) | 0.31(-0.24,0.85) |
|  | Algeria | 91004.81(51532.31,143006.84) | 64.25(-13.98,234.2) | 217.47(123.14,341.74) | -0.76(-48.03,101.91) | 217.58(121.11,341.53) | 7.83(-44.19,123.63) | 0.58(0.53,0.63) |
|  | Suriname | 2406.96(1342.36,3717.81) | 72.61(-3.64,236.35) | 417.96(233.09,645.58) | 15.88(-35.31,125.8) | 417.08(232.7,647.22) | 14.04(-35.86,120.25) | 0.55(0.3,0.81) |
|  | Panama | 14523.72(8186.03,23648.03) | 109.89(18.46,287.25) | 349.09(196.76,568.4) | 20.49(-32,122.32) | 349(196.85,569.11) | 18.43(-32.88,118.31) | 0.66(0.54,0.77) |
|  | Turkmenistan | 13541.68(6520.91,23334.58) | 78.69(-18.25,300.3) | 266.41(128.29,459.06) | 30.25(-40.41,191.79) | 264.55(126.15,459.14) | 35.06(-39.18,218.85) | 1.13(1,1.27) |
|  | Peru | 129801.51(77810.58,201274.99) | 94.83(13.64,265.94) | 381.82(228.89,592.07) | 24.53(-27.36,133.91) | 382.5(228.41,593.01) | 19.49(-29.67,120.98) | 0.41(0.35,0.46) |
|  | Mauritius | 4573.99(2350.43,7698.19) | 48.3(-24.72,182.91) | 358.28(184.11,602.99) | 27.78(-35.13,143.78) | 356.54(188.63,594.98) | 26.94(-33.79,142) | 0.78(0.61,0.95) |
|  | Albania | 6427.42(3231.22,11320.13) | -21.73(-62.07,74.04) | 236.27(118.78,416.13) | -4.77(-53.85,111.76) | 240.62(124.68,414.66) | -0.56(-51.65,118.58) | 0.32(0.19,0.45) |
|  | Uzbekistan | 90769.48(45937.73,154623.23) | 101.33(-3.67,362.2) | 269.53(136.41,459.13) | 25.23(-40.08,187.5) | 265.99(131.33,453.77) | 27.59(-40.56,201.98) | 0.92(0.86,0.98) |
|  | Brazil | 674167.71(448072.16,928559.34) | 62.29(27.31,108.74) | 311.16(206.8,428.57) | 11.48(-12.55,43.39) | 310.89(205.48,427.22) | 5.31(-15.9,34.7) | 0.38(0.32,0.45) |
|  | Tonga | 210.7(118.46,337.58) | 10.87(-33.03,94.5) | 205.86(115.74,329.83) | 4.85(-36.67,83.93) | 203.42(113.62,326.92) | 4.75(-36.88,84.15) | 0.08(-0.8,0.97) |
|  | Ecuador | 63426.61(31499.5,102816.56) | 65.17(-18.7,265.7) | 360.62(179.09,584.57) | -5.84(-53.65,108.46) | 364.58(181.42,590.74) | -11.23(-56.33,96.47) | -0.8(-0.87,-0.73) |
|  | Samoa | 429.47(245.5,702.82) | 29.96(-27.86,136) | 203.2(116.16,332.53) | 0.62(-44.15,82.72) | 203.38(115.7,334.51) | 0.22(-45.47,80.72) | 0.06(-0.58,0.71) |
|  | Cuba | 20997.44(11721.77,34384.73) | -10.49(-51.18,61.97) | 184.86(103.2,302.72) | -14.64(-53.45,54.47) | 196.6(112.53,322.12) | -8.37(-49.09,63.3) | -0.11(-0.18,-0.04) |
|  | Equatorial Guinea | 6977.55(3441.24,11648.72) | 456.28(160.04,1209.93) | 491.43(242.37,820.43) | 68.6(-21.18,297.03) | 504.62(249.13,837.05) | 60.84(-24.26,262.54) | 2.1(1.83,2.37) |
|  | Colombia | 162408.46(90851.78,266065.72) | 55.34(-21.87,234.02) | 339.93(190.16,556.89) | 5.83(-46.77,127.55) | 339.7(190.82,552.23) | 6.04(-46.55,123.66) | 0.35(0.31,0.39) |
|  | Tunisia | 22895.12(13441.24,36374.24) | 48.77(-10.63,160.36) | 197.86(116.16,314.34) | 8.5(-34.82,89.88) | 203.96(119.62,322.53) | 18.94(-28.96,109.17) | 0.74(0.65,0.83) |
|  | Republic of Moldova | 8741.03(5110.43,13432.14) | -31.17(-56.32,12.22) | 237(138.56,364.19) | -17.02(-47.34,35.29) | 235.72(138.55,358.08) | -17.41(-47.66,37.01) | -0.76(-0.86,-0.67) |
| Low middle SDI | Maldives | 1201.92(696.62,1879.71) | 117.36(16.72,347.01) | 241.15(139.77,377.14) | -3.22(-48.03,99.03) | 247.53(138.62,393.53) | 2.91(-46.49,126.23) | 0.15(-0.33,0.63) |
|  | Philippines | 244323.59(162279.22,339451.24) | 140.34(103.49,190.82) | 217.87(144.71,302.7) | 35.64(14.84,64.13) | 216.03(142.73,299.47) | 37.37(17.36,64.33) | 1.16(1.1,1.22) |
|  | Ghana | 114130.02(66569.58,177806.43) | 199.26(68.64,530.35) | 361.9(211.09,563.82) | 42.5(-19.7,200.15) | 362.37(208.38,564.81) | 36.18(-22.61,177.23) | 1.01(0.94,1.09) |
|  | Myanmar | 127869.67(66256.41,210057.04) | 83.92(-9.63,328.57) | 233.86(121.18,384.18) | 38.26(-32.06,222.18) | 235.48(121.29,387.51) | 38.58(-32.57,217.6) | 1.3(1.23,1.37) |
|  | Sudan | 75128.77(40127.02,125866.02) | 128.63(13.82,401.9) | 184.1(98.33,308.43) | 13.16(-43.66,148.42) | 171.32(90.57,291.77) | 17.77(-43.05,168.49) | 0.96(0.88,1.03) |
|  | Zambia | 66169.01(33826.76,112781.47) | 168.46(25.73,634.75) | 362.81(185.48,618.4) | 16.92(-45.24,220) | 359.69(180.56,614.61) | 8.59(-49.18,180.17) | 0(-0.09,0.08) |
|  | Democratic People's Republic of Korea | 34952.1(19081.83,55604.08) | 4.83(-40.27,95.96) | 133.24(72.74,211.96) | -15.86(-52.06,57.28) | 137.18(74.95,217.43) | -13.42(-51.11,61.31) | -0.41(-0.46,-0.35) |
|  | Dominican Republic | 40975.61(24351.09,62435.86) | 122.28(34.97,295.56) | 376.55(223.78,573.76) | 47.13(-10.66,161.84) | 376.57(223.05,574.43) | 46.46(-11.3,160.54) | 1.46(1.38,1.53) |
|  | Guatemala | 54019.14(27232.72,91049.14) | 110.93(4.01,349.78) | 303.88(153.2,512.19) | -5.47(-53.39,101.57) | 311.27(159.03,523.08) | -9.22(-54.05,92.95) | -0.31(-0.37,-0.25) |
|  | El Salvador | 16996.19(8712.43,28690.31) | 21.19(-42.5,150.36) | 271.67(139.26,458.59) | 2.04(-51.59,110.8) | 270.9(139.45,455.03) | 0.25(-51.34,105.97) | -0.11(-0.2,-0.01) |
|  | Honduras | 33767.58(17368.66,55389.05) | 100.31(3.2,309.71) | 344.06(176.97,564.37) | -3.9(-50.49,96.56) | 354.68(183.5,583.74) | -6.89(-51.91,83.44) | -0.27(-0.35,-0.19) |
|  | Timor-Leste | 2844.54(1576.35,4442.47) | 113.44(12.91,314.11) | 213.1(118.09,332.81) | 25.2(-33.77,142.9) | 206.64(113.58,325.31) | 21.44(-34.52,136.03) | 1.03(0.71,1.36) |
|  | Guyana | 2918.17(1668.29,4460.02) | 10.57(-36.64,103.79) | 378.64(216.46,578.69) | 10.48(-36.7,103.62) | 378.32(218.76,578.25) | 8.65(-38.22,100.85) | 0.14(-0.08,0.37) |
|  | Namibia | 8528.82(4820.75,13277.58) | 97.9(18.62,260.63) | 354.91(200.6,552.51) | 16.09(-30.42,111.55) | 363.72(203.07,569.64) | 12.13(-34,105.35) | 0.21(0.06,0.37) |
|  | Kyrgyzstan | 16350.21(8756.56,26694.38) | 47.12(-25.74,213.45) | 250.18(133.99,408.45) | 0.45(-49.3,114.01) | 245.98(129.97,403.71) | 1.22(-49.6,120.98) | 0.03(-0.08,0.14) |
|  | Tajikistan | 26246.37(12795.19,43572.74) | 91.12(-18.87,395.74) | 276.5(134.79,459.03) | 8.23(-54.05,180.74) | 273.14(129.31,453.94) | 9.14(-54.89,196.06) | 0.5(0.4,0.6) |
|  | India | 3654567.63(2410463.5,4985958.94) | 122.82(84.34,180.51) | 262.78(173.33,358.52) | 37.08(13.41,72.57) | 264.59(175.66,361.58) | 34.25(11.74,66.37) | 1.16(1.07,1.25) |
|  | Tuvalu | 21.08(10.78,34.2) | 45(-25.91,196.41) | 178.64(91.4,289.89) | 14.87(-41.31,134.81) | 177.37(90.12,289.42) | 14.89(-41.69,136.14) | 0.6(-2.24,3.53) |
|  | Nicaragua | 16219.62(8796.28,26914.03) | 43.33(-26.66,195.45) | 249.14(135.11,413.4) | -14.42(-56.21,76.4) | 252.93(138.09,419.63) | -15.74(-55.76,69.46) | -0.7(-0.8,-0.59) |
|  | Viet Nam | 197301.3(109900.44,308590.63) | 63.95(-3.84,194.5) | 204.73(114.04,320.2) | 15.59(-32.21,107.62) | 210.53(117.1,328.95) | 24.4(-28.3,126.28) | 0.99(0.95,1.02) |
|  | Kenya | 184985(121701.76,256025.96) | 180.74(134.23,244.02) | 368.29(242.3,509.73) | 29.63(8.16,58.85) | 358.33(234.92,495.38) | 24.51(4.42,50.75) | 0.79(0.68,0.89) |
|  | Kiribati | 242.9(115.29,414.81) | 62.42(-23.88,281.71) | 204.77(97.19,349.69) | 1.41(-52.47,138.33) | 202.5(96.06,348.62) | -0.92(-54.34,132.94) | -0.11(-1.06,0.84) |
|  | Nigeria | 612458.78(394301.49,882601.43) | 171.33(115.71,251.55) | 285.1(183.55,410.85) | 13.91(-9.44,47.58) | 288.66(186.42,412.75) | 9.42(-12.51,40.49) | 0.3(0.2,0.39) |
|  | Lesotho | 6134.54(3270.86,9911.46) | 55.96(-21.35,219.07) | 293.3(156.38,473.87) | 34.76(-32.04,175.69) | 300.81(160.46,483.9) | 32.05(-32.67,164.71) | 1.2(1.03,1.37) |
|  | Palestine | 11177.53(6336,17113.84) | 157.96(39.99,386.8) | 225.51(127.83,345.27) | 7.73(-41.53,103.3) | 214.39(120.04,329.15) | 12.24(-40.95,118.29) | 0.47(0.27,0.67) |
|  | Morocco | 81201.87(44075.24,133610.44) | 58.31(-16.91,204.09) | 225.86(122.59,371.63) | 11.39(-41.54,113.96) | 227.09(122.71,374.64) | 20.72(-37.34,139.35) | 0.99(0.94,1.05) |
|  | Mauritania | 15525.38(8816.15,24578.6) | 174.12(37.81,546.46) | 386.75(219.62,612.28) | 41.11(-29.06,232.77) | 369.92(206.11,593.94) | 39.52(-33.56,250.71) | 1.06(0.91,1.21) |
|  | Marshall Islands | 108.5(53.84,177.6) | 27.65(-34.7,163.85) | 190.88(94.72,312.44) | 2.7(-47.47,112.26) | 189.44(93.71,313.7) | 3.88(-47.93,118.59) | 0.01(-1.41,1.45) |
|  | Venezuela (Bolivarian Republic of) | 112901.52(64137.05,181676.24) | 53.78(-10.44,192.7) | 402.23(228.5,647.25) | 3.16(-39.92,96.36) | 402.96(229.45,648.85) | -0.47(-42.82,86.52) | -0.09(-0.13,-0.06) |
|  | Mongolia | 9956.88(5356.92,15652.68) | 106.57(11.25,341.36) | 293.92(158.13,462.06) | 31.33(-29.27,180.6) | 299.23(157.88,473.62) | 30.88(-30.46,171.66) | 0.86(0.71,1.01) |
|  | Eswatini | 3851.57(2054.5,6142.41) | 81.26(-5.42,255.4) | 337.23(179.89,537.81) | 28.03(-33.19,151.03) | 350.37(188.19,565.64) | 19.34(-37.31,127.85) | 0.43(0.2,0.66) |
|  | Micronesia (Federated States of) | 194.62(100.33,316.94) | -5.18(-50.79,95.07) | 190.59(98.26,310.37) | -3.16(-49.74,99.21) | 189.43(96.52,310.09) | -2.55(-50.62,104.9) | -0.18(-1.1,0.75) |
|  | Lao People's Democratic Republic | 14934.34(8174.73,23819.41) | 116.51(8.38,354.94) | 208.63(114.2,332.75) | 25.57(-37.14,163.86) | 209.58(113.45,337.4) | 24.71(-38.1,158.63) | 0.72(0.59,0.85) |
|  | Sao Tome and Principe | 867.5(501.28,1349.22) | 139.02(33.95,378.49) | 422.38(244.07,656.92) | 41.43(-20.74,183.14) | 421.21(242.9,651.51) | 37.17(-22.77,164.9) | 1.14(0.56,1.72) |
|  | Bolivia (Plurinational State of) | 35145.42(19863.07,56669.41) | 78.61(-9.56,321.46) | 292.59(165.36,471.78) | -4.52(-51.66,125.3) | 297.69(169.44,479.3) | -8.96(-53.64,107.4) | -0.18(-0.26,-0.11) |
|  | Cameroon | 97616.06(48165.42,163829.31) | 205.4(40.02,650.27) | 335.43(165.51,562.95) | 9.05(-50,167.89) | 344.87(170.87,578.75) | 3.4(-52.17,137.86) | -0.06(-0.15,0.02) |
|  | Congo | 23410.3(12431.31,38333.56) | 142.14(15.74,467.91) | 444.57(236.07,727.97) | 12.42(-46.26,163.67) | 453.84(241.75,744.9) | 6.95(-49.1,143.39) | -0.02(-0.12,0.09) |
|  | Syrian Arab Republic | 26986.86(14952.41,43603.97) | 5.06(-43.41,95.18) | 186.23(103.18,300.9) | -6.51(-49.65,73.68) | 184.39(100.95,297.15) | 3.66(-44.16,97.1) | 0.02(-0.07,0.11) |
|  | Belize | 1369.93(790.14,2171.38) | 180.51(54.17,405.21) | 334.05(192.67,529.48) | 27.15(-30.12,129) | 334.09(192.79,534.09) | 24.27(-31.9,124.3) | 0.52(0.07,0.96) |
|  | Cabo Verde | 2729.24(1549.24,4211.05) | 113.49(17.69,304.55) | 484.28(274.9,747.22) | 33.2(-26.57,152.4) | 489.2(278.09,750.75) | 28.57(-29.58,141.44) | 0.81(0.54,1.09) |
|  | Nauru | 21.62(11.27,35.55) | -1.6(-50.14,104.2) | 204.94(106.8,336.92) | -4.37(-51.54,98.46) | 200.96(101.72,335.5) | -7.71(-54.51,98.98) | -0.43(-3.62,2.87) |
| Low SDI | Vanuatu | 557.68(288.08,891.42) | 97.28(1.71,275.66) | 189.33(97.8,302.64) | 1.39(-47.73,93.07) | 185.18(94.25,294.94) | -0.42(-49.52,94.82) | -0.16(-0.85,0.53) |
|  | Yemen | 68544.57(39268.13,108264.99) | 144.7(33.42,369.56) | 217.58(124.65,343.67) | 6.64(-41.85,104.64) | 196.43(111.79,315.33) | 12.44(-40.28,126.81) | 0.93(0.82,1.03) |
|  | Uganda | 142923.53(69996.69,238732.53) | 203.35(37.49,679.01) | 347.59(170.23,580.61) | 27.74(-42.1,228.05) | 337.82(167.06,561.09) | 19.13(-45.34,191.83) | 0.3(0.16,0.43) |
|  | Gambia | 6981.87(3800.2,11330.02) | 231.45(69.49,605.3) | 310.88(169.21,504.48) | 46.4(-25.14,211.51) | 293.75(155.57,486.08) | 44.86(-29.3,228.83) | 1.51(1.25,1.78) |
|  | Afghanistan | 80857.64(42485.63,135969.96) | 251.44(69.14,709.25) | 211.24(110.99,355.22) | 4.84(-49.54,141.41) | 188.27(94.72,324.91) | 1.91(-53.48,146.23) | 0.2(0.11,0.28) |
|  | Djibouti | 4100.52(2227.93,6941.44) | 208.58(46.44,716.24) | 340.92(185.23,577.11) | 24.69(-40.83,229.81) | 334.27(174.65,577.61) | 26.91(-43.01,267.14) | 0.72(0.38,1.06) |
|  | Guinea | 32701.34(14802.52,57796.89) | 152.14(1.93,623.02) | 258.65(117.08,457.14) | 23.37(-50.13,253.78) | 251.52(109.19,448.76) | 22.86(-51.95,256.06) | 0.63(0.53,0.73) |
|  | Burundi | 34367.07(16666.81,58880.04) | 121.62(-0.58,483.26) | 287.97(139.65,493.37) | 3.45(-53.59,172.26) | 283.48(139.84,481.43) | -3.69(-56.15,144.71) | -1.35(-1.46,-1.23) |
|  | Bangladesh | 368396.86(214746.92,587813.38) | 52.85(-11.78,174.99) | 231.32(134.84,369.09) | 4.67(-39.59,88.3) | 228.47(132.88,361.23) | 11.45(-38.3,110.32) | 0.71(0.61,0.82) |
|  | Bhutan | 2378.89(1412,3656.71) | 30.38(-24.79,143.62) | 315.4(187.21,484.81) | 5.82(-38.96,97.73) | 316.92(187.86,485.94) | 4.53(-38.86,91.93) | 0.21(-0.07,0.49) |
|  | Eritrea | 23451.26(12709.27,39859.05) | 211.45(43.22,652.98) | 349.43(189.37,593.92) | 39.29(-35.95,236.75) | 338.81(180.17,582.17) | 31.81(-41.21,223.04) | 0.64(0.5,0.77) |
|  | Cambodia | 35293.92(20287.78,56215.31) | 109.73(11.8,347.43) | 212.57(122.19,338.58) | 31.06(-30.14,179.6) | 211.69(120.71,339.41) | 34.56(-28.51,186.39) | 1.22(1.13,1.31) |
|  | Comoros | 2557.93(1421.12,4168.05) | 93.17(-5.13,392.98) | 358.08(198.94,583.47) | 25.95(-38.14,221.42) | 347.27(189.79,563.73) | 28.55(-39.67,252.25) | 0.91(0.57,1.25) |
|  | Guinea-Bissau | 6073.17(3032.05,10008.24) | 140.09(0.35,536.04) | 319.44(159.48,526.42) | 27.21(-46.83,236.99) | 311.07(148.99,515.15) | 19.23(-50.31,211.05) | 0.28(0.05,0.52) |
|  | Liberia | 14765.18(7605.04,25002.73) | 155.76(18.68,511.46) | 308.26(158.77,521.99) | 4.88(-51.33,150.74) | 314.84(163.66,528.55) | 1.91(-50.96,128.69) | 0.35(0.21,0.5) |
|  | Madagascar | 75514.11(39879.04,124828.28) | 137.7(10.37,466.5) | 282.93(149.41,467.69) | 6.44(-50.58,153.67) | 278.05(145.64,464.37) | 2.33(-52.07,139.29) | -0.01(-0.11,0.09) |
|  | Mali | 45552.62(20754.94,81436.3) | 240.5(35.39,783.31) | 207.84(94.7,371.56) | 34.74(-46.43,249.53) | 195.69(84.89,354.75) | 32.29(-49.98,255.07) | 0.36(0.25,0.48) |
|  | Haiti | 32564.11(16725.23,54311.52) | 74.32(-17.28,283) | 262.57(134.86,437.92) | -10.66(-57.61,96.28) | 267.24(137.25,447.96) | -11.86(-57.82,92.53) | -0.54(-0.62,-0.47) |
|  | Pakistan | 618154.63(383149.6,941563.61) | 167.97(61.91,374.67) | 275.88(171,420.22) | 34.95(-18.46,139.04) | 263.57(161.5,408.3) | 30.46(-23.48,146.07) | 0.95(0.85,1.05) |
|  | Ethiopia | 302906.27(189642.59,437345.99) | 253.09(131.21,513.04) | 281.53(176.26,406.49) | 68.66(10.44,192.82) | 268.84(164.82,388.62) | 53.03(1.68,160.45) | 1.11(0.98,1.24) |
|  | Zimbabwe | 45144.79(24841.23,72894.08) | 67.48(-9.1,210.48) | 300.75(165.49,485.61) | 15.34(-37.4,113.83) | 321.75(175.27,515.3) | 8.08(-41.07,99.81) | 0.03(-0.08,0.14) |
|  | Mozambique | 96515.81(46309.48,168316.08) | 215.81(36.76,763.61) | 326.86(156.83,570.02) | 39.8(-39.46,282.29) | 323.21(154.66,569.53) | 32.97(-41.02,243.44) | 0.97(0.79,1.16) |
|  | Angola | 121812.33(60112.99,204623.1) | 247.98(58.93,800.67) | 404.17(199.46,678.94) | 19.13(-45.59,208.36) | 423.36(213.17,705.14) | 13.54(-46.3,171.41) | 0.63(0.57,0.69) |
|  | South Sudan | 26052.47(10806.77,49267.79) | 73.23(-29.86,443.33) | 280.65(116.42,530.73) | 9.34(-55.73,242.96) | 277.13(110.75,529.62) | 7.08(-58.34,244.09) | 0.33(0.23,0.43) |
|  | Nepal | 107600.41(61368.95,167396.23) | 66.16(-5.9,214.55) | 353.76(201.76,550.35) | 6.72(-39.56,102.04) | 355.62(203.46,555.47) | 3.96(-40.66,93.55) | 0.52(0.47,0.58) |
|  | Burkina Faso | 53914.23(23373.27,99474.47) | 160.93(12.73,564.95) | 237.59(103,438.37) | 9.9(-52.52,180.08) | 232.71(99.24,430.41) | 4.75(-55.61,167.27) | 0.14(0.06,0.23) |
|  | Niger | 49510.27(23205.68,90548.79) | 206.49(21.61,797.16) | 212.53(99.62,388.7) | 5.56(-58.12,208.98) | 197.64(89.87,370.61) | 3.36(-60.33,220.4) | 0.17(0.07,0.27) |
|  | Democratic Republic of the Congo | 277287.44(130139.47,480219.72) | 168.5(26.61,516.19) | 316.28(148.44,547.76) | 18.19(-44.27,171.23) | 321.69(152.76,553.96) | 11.47(-45.89,143.29) | 0.43(0.33,0.53) |
|  | Malawi | 53682.09(27511.11,88338.57) | 164.52(27.84,528.13) | 291.08(149.17,479) | 37.06(-33.76,225.46) | 279.83(143.02,462.31) | 25.07(-39.34,187.71) | 0.58(0.45,0.7) |
|  | Papua New Guinea | 17204.49(8224.32,29720.97) | 159.39(21.95,445.72) | 174.37(83.36,301.23) | 7.46(-49.48,126.08) | 173.47(82.34,303.03) | 5.01(-50.93,121.18) | 0.04(-0.09,0.17) |
|  | Central African Republic | 15779.59(7141.54,27761.72) | 91.44(-16.98,347.47) | 297.74(134.75,523.82) | -0.88(-57.02,131.68) | 308.8(143.23,536.14) | -5.58(-56.68,108.24) | -0.39(-0.51,-0.27) |
|  | Benin | 39313.2(19327.24,65253.3) | 165.44(12.52,558.86) | 310.39(152.59,515.19) | 1.7(-56.89,152.43) | 324.04(161.55,545.8) | -4.65(-56.56,112.6) | -0.62(-0.77,-0.48) |
|  | Rwanda | 37862.67(19869.33,61525.53) | 106.86(-1.52,441.88) | 298.41(156.6,484.91) | 16.94(-44.33,206.34) | 295.94(155.95,483.71) | 9.78(-46.87,174.45) | 0.23(0.1,0.36) |
|  | Senegal | 53516.8(28503.4,86288.24) | 194.56(42.05,636.22) | 353.62(188.34,570.16) | 48.33(-28.47,270.72) | 339.07(176,557.5) | 50.84(-30.23,300.59) | 1.22(1.05,1.39) |
|  | Solomon Islands | 1183.93(626.3,2047.9) | 95.67(-1.38,318.41) | 180.58(95.53,312.35) | 1.6(-48.79,117.27) | 176.02(90.98,310.66) | -0.75(-51.49,121.16) | -0.23(-0.73,0.26) |
|  | Togo | 26125.75(14609.53,43299.85) | 99.95(0.09,367.62) | 329.81(184.43,546.61) | -7.54(-53.72,116.23) | 336.47(187.78,552.35) | -11.2(-54.52,92.96) | -0.61(-0.71,-0.51) |
|  | Côte d'Ivoire | 84950.59(43875.3,140450.99) | 158.55(23.59,518.92) | 324.59(167.65,536.66) | 20.78(-42.26,189.14) | 337.08(175.6,557.2) | 13.6(-44.48,155.63) | 0.14(0.03,0.25) |
|  | Sierra Leone | 19188.15(8297.39,34600.22) | 147.48(0.2,666.1) | 231.61(100.15,417.64) | 9.1(-55.83,237.71) | 225.1(95.25,408.79) | 8.61(-57.64,246.16) | 0.17(0.04,0.3) |
|  | United Republic of Tanzania | 168279.65(86058.97,278095.72) | 212.83(41.98,708.26) | 296.6(151.68,490.16) | 42.81(-35.18,268.99) | 286.68(145.86,477.17) | 34.15(-39.53,250.44) | 0.5(0.39,0.61) |
|  | Somalia | 41691.55(18906.84,73908.16) | 246.67(39.66,876.95) | 204.94(92.94,363.31) | 21.8(-50.93,243.25) | 187.81(80.77,332.23) | 16.06(-55.98,249.54) | 0.31(0.19,0.43) |
|  | Chad | 39157.13(16259.6,73718.66) | 229.17(30.22,825.21) | 238.78(99.15,449.54) | 20.88(-52.18,239.76) | 235.15(94.42,444.46) | 18.99(-53.94,234.56) | 0.69(0.58,0.79) |

**Table S5. Trends in epilepsy Prevalence for both genders in 204 countries and regions, 1990-2019.**

| **SDI quintile** | **Country** | **Deaths** | | **All-age Prevalence** | | **Age-standardized Prevalence** | | **Net drift of Prevalence from APC model,% per year** |
| --- | --- | --- | --- | --- | --- | --- | --- | --- |
|  |  | **Number in 2019** | **Percent change 1990-2019, %** | **Rate in 2019** | **Percent change 1990-2019, %** | **Rate in 2019** | **Percent change 1990-2019, %** |  |
| High SDI | Denmark | 31070.35(16030.68,42426.52) | 28.77(-33.5,162.82) | 535.44(276.26,731.15) | 14.15(-41.04,132.99) | 520.02(287.01,702.01) | 9.71(-41.08,116.03) | 0.72(0.66,0.78) |
|  | Puerto Rico | 40269.79(26239.58,51620.16) | -1.27(-39.27,63.83) | 1143.56(745.14,1465.89) | 1.3(-37.68,68.1) | 1061.84(701.93,1366.25) | -5.58(-41.77,56.62) | -0.37(-0.42,-0.32) |
|  | Switzerland | 48878.02(27542.34,65283.73) | 30.49(-26.81,156.75) | 557(313.87,743.96) | 2.09(-42.74,100.87) | 538.66(323.18,712.63) | 0.27(-40.86,89.63) | 0(-0.05,0.05) |
|  | Russian Federation | 847868.44(701123.25,998374) | 1.08(-9.26,11.68) | 577.89(477.87,680.47) | 4.04(-6.59,14.95) | 564.86(467.92,670.88) | 2.13(-8.47,12.91) | 0.01(-0.03,0.06) |
|  | Germany | 772132.97(346476.58,1062021.4) | 53.57(-32.57,216.4) | 909.31(408.03,1250.7) | 44.58(-36.52,197.88) | 881.53(426.66,1197.48) | 38.68(-34.93,175.29) | 1.48(1.43,1.54) |
|  | Finland | 30362.3(14468.61,42034.28) | 18.4(-46.69,149.17) | 548.64(261.44,759.55) | 7.19(-51.74,125.58) | 538.07(272.27,731.42) | 6.58(-48.79,116.92) | 0.19(0.13,0.24) |
|  | France | 416603.43(181405.38,589595.57) | 29.46(-44.66,180.26) | 629.27(274.01,890.57) | 12.96(-51.71,144.56) | 583(275.19,825.38) | 6.34(-51.94,124.67) | 0.15(0.08,0.21) |
|  | Czechia | 85645.27(44336.38,118368.71) | 28.05(-40.66,162.67) | 804.67(416.56,1112.12) | 23.89(-42.58,154.13) | 776.37(433.93,1054.99) | 19.6(-40.55,136.12) | 0.76(0.73,0.8) |
|  | United Kingdom | 411875.38(331287.18,493979.54) | 22.2(9.12,34.82) | 612.72(492.84,734.87) | 4.48(-6.71,15.27) | 619.24(499.77,744.87) | 5.78(-5.46,16.33) | 0.54(0.4,0.68) |
|  | San Marino | 193.68(116.96,253.08) | 43.43(-14.8,138.78) | 585.15(353.34,764.59) | 2.07(-39.37,69.94) | 559.7(339.47,736.85) | 0.6(-40.49,68.23) | 0.1(-0.69,0.9) |
|  | Brunei Darussalam | 2571.89(1505.12,3500.3) | 53.75(-18.31,225.3) | 588.37(344.33,800.77) | -9.06(-51.68,92.41) | 609.05(349.68,830.13) | -6.3(-51.87,104.04) | -0.18(-0.46,0.09) |
|  | Kuwait | 31339.92(20143.86,41037.17) | 150.56(54.83,335.32) | 708(455.07,927.07) | -0.41(-38.46,73.03) | 748.12(464.99,989.4) | 7.35(-35.84,99.13) | 0.26(0.16,0.37) |
|  | Ireland | 28096.28(14262.4,38305.64) | 54.23(-23.51,217.51) | 572.18(290.46,780.1) | 13.12(-43.9,132.86) | 567.1(292.78,769.54) | 12.86(-42.84,130.19) | 0.37(0.31,0.44) |
|  | Republic of Korea | 283260.01(180670.23,363100.83) | 26.42(-23.67,120.67) | 530.47(338.34,679.99) | 4.97(-36.62,83.23) | 523.21(334.6,676.84) | 1.27(-39.41,73.61) | -0.12(-0.14,-0.1) |
|  | Taiwan (Province of China) | 140352.18(81966.49,184894.47) | 64.43(-8.59,182) | 594.2(347.02,782.78) | 42(-21.05,143.55) | 551.15(325.25,725.91) | 27.38(-28.64,121.1) | 1.1(1.03,1.17) |
|  | Luxembourg | 4447.49(2250.67,6018.7) | 89.11(-3.67,263.8) | 719.02(363.86,973.03) | 16.56(-40.62,124.24) | 691.15(363.04,930.45) | 13.22(-39.73,113.52) | 0.52(0.35,0.7) |
|  | Singapore | 25898.99(17166.38,33218.16) | 109.57(34.33,238.02) | 456.98(302.89,586.12) | 12.68(-27.77,81.74) | 489.64(326.44,635.05) | 19.16(-24.26,96.36) | 0.77(0.69,0.85) |
|  | Slovenia | 14355.4(8185.1,19365.84) | 9.71(-41.25,137.66) | 692.07(394.6,933.62) | 4.25(-44.17,125.84) | 678.62(407.36,902.55) | 3.62(-41.47,118.5) | 0.04(-0.04,0.13) |
|  | United States of America | 2322702.64(1936754.8,2693228.88) | 31.06(17.4,45.76) | 708.19(590.51,821.16) | 1.34(-9.22,12.71) | 671.16(563.63,774.31) | -2.86(-12.57,8.19) | 0.45(0.4,0.49) |
|  | Australia | 131042.02(74007.11,174185.91) | 38.13(-24.38,140.42) | 533.38(301.23,708.99) | -5.21(-48.1,65) | 535.44(312.13,704.54) | -5.16(-46.13,61.89) | -0.18(-0.21,-0.14) |
|  | Iceland | 1904.77(984.46,2561.75) | 56.5(-18.49,217.99) | 552.31(285.45,742.8) | 15.25(-39.97,134.17) | 543.91(287.98,735.36) | 14.03(-38.29,127.66) | 0.53(0.26,0.79) |
|  | Canada | 163255.55(101203.3,216171.6) | 38.95(-16.57,127.92) | 447.03(277.12,591.93) | 3.71(-37.73,70.11) | 449.77(291.46,586.06) | 4.27(-35.79,67.42) | 0.14(0.09,0.18) |
|  | Japan | 538414.26(428806.59,653815.74) | 10.56(-4.45,27.1) | 421.33(335.56,511.64) | 8.9(-5.89,25.19) | 440.56(354.91,536.18) | 10.63(-2.74,25.82) | 0.36(0.3,0.43) |
|  | Slovakia | 51000.24(29790.19,67231.21) | 23.21(-32.33,119.49) | 937.98(547.89,1236.5) | 19.72(-34.25,113.26) | 843.02(491.1,1114.29) | 10.16(-40.65,95.71) | 0.36(0.32,0.41) |
|  | New Zealand | 24488.42(15152.32,31858.67) | 33.45(-20.87,133.57) | 544.71(337.04,708.65) | 1.44(-39.85,77.54) | 562.27(351.36,723.31) | 4.43(-36.09,81.25) | 0.21(0.14,0.28) |
|  | Estonia | 11658.05(7119.26,15340.99) | -1.46(-42.75,68.19) | 888.33(542.48,1168.96) | 17.79(-31.57,101.04) | 784.44(473.13,1038.59) | 7.67(-37.24,83.52) | 0.6(0.5,0.69) |
|  | Belgium | 80009.9(38362.52,109812.31) | 39.23(-36.49,191.74) | 700.66(335.95,961.65) | 21.68(-44.49,154.96) | 657.47(343.64,891.86) | 14.51(-44.28,128.3) | 0.64(0.6,0.68) |
|  | Qatar | 20077.62(13357.04,26644.74) | 562.94(294.63,1047.43) | 700.9(466.29,930.16) | 3.02(-38.68,78.3) | 754.32(477.27,1004.79) | 9.27(-38.8,102.24) | 0.47(0.25,0.69) |
|  | Saudi Arabia | 299586.19(168639.19,411712.07) | 191.94(45.56,466.73) | 838.43(471.96,1152.22) | 31.09(-34.64,154.49) | 864.83(472.08,1194.94) | 38.81(-33.47,183.17) | 1.38(1.34,1.41) |
|  | Latvia | 14837.01(9291.27,19290.17) | -20.01(-52.76,25.51) | 774.66(485.11,1007.17) | 11.04(-34.43,74.22) | 710.61(455.65,921.88) | 4.93(-37.28,63.01) | 0.34(0.26,0.42) |
|  | Austria | 61155.67(39032.01,79564.57) | 24.23(-23.84,111.66) | 685.9(437.77,892.36) | 8.24(-33.64,84.42) | 638.2(409.6,837.54) | 4.58(-36.02,77.42) | 0.08(0.04,0.13) |
|  | Andorra | 431.43(230.72,576.22) | 48.56(-21.17,173.03) | 519.4(277.76,693.71) | -3.26(-48.67,77.8) | 523.73(288.09,705.42) | -2.7(-47.58,79.57) | -0.06(-0.58,0.45) |
|  | Netherlands | 105614.27(53381.82,142433.85) | 32.72(-31.92,143.98) | 615.58(311.14,830.19) | 15.44(-40.78,112.21) | 599.59(326.28,806.02) | 16.58(-38.09,107.01) | 0.51(0.37,0.64) |
|  | United Arab Emirates | 66388.43(39439.33,89915.51) | 365.28(148.83,799.85) | 718.36(426.75,972.93) | -5.75(-49.59,82.28) | 790.88(438.71,1081.41) | -1.03(-49.98,107.73) | -0.26(-0.36,-0.16) |
|  | Lithuania | 22436.32(13484.28,29633.29) | -16.68(-52.38,48.85) | 802.95(482.58,1060.52) | 9.53(-37.39,95.69) | 714.77(433.42,931.57) | 0.97(-43.81,80.32) | 0.22(0.16,0.28) |
|  | Bermuda | 644.06(392.44,846.88) | 4.66(-39.36,80.32) | 1005.87(612.89,1322.63) | -2.82(-43.7,67.43) | 904.14(543.9,1188.84) | -11.6(-48.71,54.87) | -0.52(-0.92,-0.13) |
|  | Cyprus | 6744.63(3905.81,9008.42) | 72.8(-6.68,211.44) | 513.49(297.36,685.85) | 2.35(-44.73,84.47) | 521.08(306.68,701.26) | 3.42(-43.45,87.02) | 0.19(0.05,0.34) |
|  | Guam | 1092.39(676.62,1438.69) | 40.94(-14.09,144.51) | 640.22(396.55,843.17) | 12.97(-31.14,95.98) | 644.9(399.91,849.22) | 12.49(-30.99,96.79) | 0.47(0.11,0.84) |
|  | Norway | 33911.07(25588.83,42381.45) | 30.18(9.68,52.89) | 633.99(478.4,792.35) | 3.36(-12.92,21.39) | 623.33(476.79,776.08) | 2.61(-12.84,20.27) | 0.15(0.09,0.2) |
|  | Sweden | 42814.68(25230.46,56904.02) | 16.15(-31.96,95.14) | 418.83(246.81,556.65) | -2.41(-42.83,63.96) | 418.01(255.78,549.9) | -4.16(-41.81,55.89) | -0.11(-0.16,-0.06) |
|  | Monaco | 289.5(174.3,382.17) | 40.19(-14.69,134.73) | 770.52(463.9,1017.17) | 13.58(-30.88,90.18) | 667.39(404.16,880.67) | 10.73(-33.44,85.45) | 0.45(-0.25,1.15) |
| High middle SDI | Dominica | 945.14(607.42,1231.62) | 6.21(-36.73,85.05) | 1376.11(884.4,1793.23) | 14.45(-31.83,99.4) | 1337.26(862.41,1748.74) | 9.88(-34.9,89.59) | 0.12(-0.22,0.46) |
|  | Croatia | 43560.35(27785.63,56190.86) | -13.08(-46.96,49.92) | 1025.46(654.1,1322.79) | 0.28(-38.81,72.96) | 895.38(577.92,1140.44) | -7.16(-43.01,64.49) | -0.25(-0.37,-0.14) |
|  | Ukraine | 271318.5(182194.31,347679.39) | -24.08(-50.91,19.98) | 616.04(413.68,789.42) | -9.22(-41.31,43.46) | 606.56(402.95,772.95) | -9.83(-42.39,41.78) | -0.42(-0.45,-0.39) |
|  | Hungary | 85226.33(52375.44,110607.59) | -7.77(-46.38,56.84) | 880.95(541.38,1143.3) | -0.93(-42.4,68.48) | 808.79(498.57,1050.19) | -5.92(-44.98,60.38) | -0.32(-0.35,-0.29) |
|  | Argentina | 243087.17(158961.84,319418.15) | 45.41(-11.64,133.87) | 538.81(352.35,708) | 6.75(-35.13,71.69) | 538.72(349.59,708.71) | 6.63(-34.89,71.55) | 0.17(0.1,0.24) |
|  | Saint Kitts and Nevis | 671.72(423.61,885.71) | 39.34(-16.33,144.85) | 1128.79(711.86,1488.39) | -3.17(-41.86,70.15) | 1106.59(699.46,1454.83) | -8.17(-44.69,61.57) | -0.65(-1.08,-0.23) |
|  | North Macedonia | 16526.19(10880.79,21446.76) | 20.39(-26.5,103.81) | 767.68(505.44,996.26) | 12.71(-31.19,90.81) | 748.09(489.97,960.49) | 8.9(-33.93,83.4) | 0.31(0.23,0.39) |
|  | Kazakhstan | 149000.47(88362.35,201300.71) | 25.47(-34.07,145.95) | 810.13(480.44,1094.5) | 11.66(-41.32,118.88) | 819.14(483.7,1108.49) | 11.58(-41.49,120.22) | 0.51(0.45,0.57) |
|  | Romania | 169406.82(106375.79,219542.74) | -7.48(-43.95,69.3) | 880.63(552.97,1141.25) | 12.52(-31.83,105.91) | 806.01(507.88,1057.32) | 5.34(-36.27,93.9) | 0.15(0.13,0.18) |
|  | Chile | 150002.85(89509.47,200192.92) | 52.96(-16.51,189.27) | 824.27(491.85,1100.06) | 11.62(-39.07,111.09) | 805.28(469.84,1084.04) | 7.76(-40.7,104.84) | 0.36(0.33,0.39) |
|  | Poland | 338270.46(270697.9,405140.59) | 41.93(19.84,66.85) | 880.12(704.31,1054.11) | 40.91(18.98,65.65) | 759.61(608.6,910.16) | 23.69(5.31,44.54) | 0.82(0.79,0.85) |
|  | Montenegro | 4917.89(3122.67,6391.95) | 0.57(-39.57,61.9) | 792.77(503.38,1030.4) | 1.45(-39.05,63.3) | 767.73(487.38,999.58) | -1.85(-41.04,58.35) | 0.01(-0.14,0.15) |
|  | Greece | 63340.24(40565.18,82044.28) | 14.47(-32.72,98.86) | 612.74(392.42,793.68) | 15.05(-32.38,99.87) | 581.33(366.65,761.46) | 12.52(-34.76,98.24) | 0.57(0.52,0.61) |
|  | Malaysia | 195493.86(120423.11,265313.61) | 89.74(12.49,231.84) | 624.55(384.72,847.61) | 7.02(-36.55,87.17) | 633.75(385.27,866.08) | 11.37(-35.32,96.93) | 0.41(0.38,0.45) |
|  | United States Virgin Islands | 1110.62(692.54,1445.32) | 17.59(-32.48,98.49) | 1068.06(666,1389.93) | 19.88(-31.16,102.36) | 1003.29(635.16,1306.55) | 10.07(-37.61,84.12) | 0.29(-0.02,0.6) |
|  | Lebanon | 34188.03(21611.43,44977.65) | 70.2(-5.28,222.97) | 660.37(417.45,868.79) | 7.67(-40.08,104.31) | 668.82(421.33,885.53) | 13.29(-37,117.08) | 0.53(0.46,0.61) |
|  | Serbia | 76034.47(47974.29,99171.54) | 3.3(-37.85,74.49) | 869.28(548.48,1133.81) | 10.98(-33.23,87.47) | 771.17(488.32,1002.95) | 0.26(-39.22,71.31) | 0(-0.04,0.04) |
|  | Antigua and Barbuda | 839.55(413.1,1193.61) | 46.05(-33.95,227.58) | 948.76(466.84,1348.87) | 0.23(-54.68,124.79) | 938.88(472.64,1329.1) | 0.05(-53.62,124.67) | -0.17(-0.57,0.22) |
|  | Israel | 58243.22(34337.21,76480.08) | 103.06(16.53,235.41) | 625.63(368.84,821.52) | 8.23(-37.89,78.77) | 612.47(362.77,805.9) | 6.21(-38.99,74.57) | 0.37(0.31,0.42) |
|  | Bulgaria | 47009.34(24390.31,65301.12) | -6.46(-52.38,100.8) | 677.89(351.72,941.67) | 17.1(-40.39,151.37) | 682.68(378.48,924.22) | 16.05(-37.12,136.81) | 0.67(0.62,0.71) |
|  | Italy | 301734.53(231778.37,369852.79) | 10.95(-5.41,31.26) | 500.28(384.29,613.22) | 4.48(-10.92,23.61) | 489.29(385.06,598.47) | 0.16(-13.39,16.94) | -0.06(-0.13,0) |
|  | Barbados | 3606.96(2507.71,4694.43) | 23.08(-19.66,101.7) | 1211.32(842.16,1576.52) | 4.94(-31.5,71.98) | 1165.92(810.26,1515.37) | 3.65(-32.16,68.34) | 0.03(-0.15,0.21) |
|  | Trinidad and Tobago | 15621.38(9865.4,20614.09) | 26.53(-26.3,123.72) | 1125.9(711.04,1485.75) | 9.71(-36.1,93.98) | 1081.23(686.72,1431.73) | 1.6(-41.15,80.86) | 0.17(0.08,0.25) |
|  | Bahamas | 3784.85(2413.78,5043.46) | 45.44(-11.46,149.37) | 1004.1(640.36,1338) | -1.04(-39.76,69.67) | 998.39(633.05,1330.41) | -5.65(-42.53,61.54) | -0.24(-0.43,-0.06) |
|  | Bosnia and Herzegovina | 28632.19(18138.7,37439.79) | -20.05(-54.74,33.17) | 867.65(549.66,1134.55) | 9.94(-37.77,83.13) | 824.34(521.51,1078.02) | 3.93(-40.67,71.2) | 0.39(0.34,0.45) |
|  | Malta | 2394.64(1437.18,3180.39) | 35.41(-23.29,144.46) | 545.2(327.21,724.1) | 14.29(-35.26,106.32) | 553.11(348,728.34) | 14.44(-32.4,103.78) | 0.68(0.46,0.9) |
|  | Jordan | 80276.57(53817.41,106323.4) | 236.65(105.29,456.15) | 689.86(462.48,913.69) | 9.16(-33.43,80.33) | 667.13(438.22,883.24) | 15.85(-30.68,94.1) | 0.68(0.6,0.76) |
|  | Belarus | 64746.12(42704.06,82790.17) | -6.48(-40.19,47.26) | 681.48(449.48,871.4) | 3.06(-34.09,62.29) | 659.23(440.7,839.68) | 0.66(-35.75,59.61) | -0.03(-0.06,0.01) |
|  | Seychelles | 726.03(470.51,968.29) | 56.11(-5.23,154.22) | 710.79(460.63,947.95) | 11.61(-32.24,81.75) | 731.28(472.75,973.84) | 17.82(-28.77,96.32) | 0.46(-0.01,0.94) |
|  | Portugal | 49886.37(28192.42,67976.01) | 36.72(-28.02,154.87) | 468.36(264.69,638.2) | 30.12(-31.5,142.56) | 449.07(278.89,598.25) | 23.02(-29.84,119.72) | 0.7(0.65,0.75) |
|  | Cook Islands | 109.59(64.86,145.5) | -0.33(-44.71,73.16) | 609.31(360.63,808.95) | 5.4(-41.53,83.12) | 611.35(365.14,814.49) | 5.9(-39.71,85.79) | 0.21(-0.82,1.25) |
|  | Libya | 38027.5(25531.92,49688.52) | 38.74(-13.76,143.47) | 564.58(379.06,737.71) | -12.73(-45.75,53.15) | 573.32(383.62,748.02) | -5.01(-41.41,72.24) | -0.17(-0.25,-0.1) |
|  | American Samoa | 342.67(192.9,472.13) | 18.5(-37.98,107.28) | 617.37(347.53,850.61) | 3.4(-45.88,80.86) | 622.88(349.42,853.92) | 2.21(-46.55,81.57) | 0.21(-0.43,0.87) |
|  | Turkey | 682405.63(423054.28,893434.97) | 76.54(-2.73,246.24) | 838.75(519.98,1098.13) | 29.7(-28.54,154.37) | 864.73(532.48,1137.02) | 40.41(-22.29,176.95) | 1.64(1.55,1.72) |
|  | Bahrain | 10426.95(6517.97,14002.76) | 179(58.75,423.19) | 722.74(451.79,970.6) | -1.75(-44.1,84.24) | 786.09(468.1,1073.64) | 2.69(-44.52,105.59) | 0.02(-0.14,0.19) |
|  | Spain | 276334.24(168963.33,360431.32) | 30.12(-19.94,127.55) | 600.45(367.14,783.19) | 9.65(-32.54,91.75) | 535.21(329.44,706.92) | 0.72(-38.47,79.9) | 0.27(0.23,0.3) |
|  | Oman | 29388.68(19536.1,38899.82) | 170.88(59.84,371.09) | 641.11(426.18,848.6) | 14.83(-32.24,99.7) | 657.9(418.77,874.8) | 27.22(-27.33,130.79) | 1.03(0.9,1.15) |
|  | Greenland | 389.58(181.21,559.35) | -7.18(-56.54,101.05) | 693.35(322.51,995.5) | -8.19(-57.01,98.86) | 680.82(333.86,967.42) | -11.8(-56.78,95.78) | -0.27(-0.77,0.25) |
|  | Niue | 9.96(6,13.09) | -24.66(-56.42,27.45) | 596.08(358.66,783.17) | 4.87(-39.34,77.39) | 596.57(360,783.19) | 5.47(-38.92,78.01) | 0.17(-3.03,3.48) |
|  | Northern Mariana Islands | 242.06(141.75,327.77) | -2.7(-44.93,67.61) | 569.63(333.58,771.32) | 3.94(-41.16,79.06) | 586.4(350.44,785.43) | 3.35(-41.08,81.54) | 0.14(-0.56,0.85) |
|  | Palau | 93.16(55.44,125.44) | 18.58(-32.75,108.96) | 517.37(307.86,696.58) | 1.43(-42.48,78.75) | 543.67(328.38,729.61) | 6.73(-39.11,89.85) | 0.2(-0.95,1.36) |
| Middle SDI | Costa Rica | 45542.7(27991.25,61576.34) | 63.19(-12.03,198.93) | 965.55(593.44,1305.48) | 5.17(-43.3,92.66) | 951.36(578.64,1288.24) | -1.73(-46.19,77.26) | -0.1(-0.15,-0.04) |
|  | Iran (Islamic Republic of) | 541662.32(448087.93,641275.79) | 86.49(51.19,134.04) | 642.56(531.55,760.73) | 29.51(4.99,62.53) | 659.92(542.26,783.78) | 45.98(18.94,81.08) | 1.57(1.5,1.64) |
|  | Gabon | 26478.75(16918.31,35255.65) | 110.22(17.19,307.27) | 1513.04(966.74,2014.57) | 19.11(-33.6,130.75) | 1563.89(1007.67,2069.62) | 12.53(-36.48,113.78) | 0.18(0.1,0.27) |
|  | Armenia | 23833.55(16066.63,31473.55) | -3.16(-40.28,60.76) | 789.28(532.06,1042.28) | 9.5(-32.47,81.78) | 788.8(527.59,1043) | 8.12(-33.97,82.33) | 0.33(0.27,0.4) |
|  | Azerbaijan | 78847.99(46108.89,109571.93) | 67.18(-13.84,273.61) | 767.1(448.59,1066.01) | 19.24(-38.55,166.47) | 786.95(460.95,1104.21) | 26.49(-35.96,186.23) | 1.26(1.18,1.33) |
|  | Grenada | 1137.63(739.16,1492.54) | 46.33(-13.1,145.16) | 1102.19(716.13,1446.05) | 21.46(-27.87,103.49) | 1088.02(707.83,1424.82) | 16.17(-29.99,92.27) | 0.37(0.03,0.72) |
|  | Iraq | 266876.46(170067.34,357925.35) | 148.87(46.75,341.59) | 633.62(403.77,849.79) | 3.97(-38.69,84.49) | 606.48(376.21,815.64) | 9.31(-37.19,101.22) | 0.55(0.51,0.59) |
|  | Tokelau | 7.28(4.66,9.79) | -9.46(-47.42,58.01) | 516.11(330.33,694.15) | 8.28(-37.12,88.97) | 517.21(328.97,694.83) | 10.48(-36.73,95.57) | 0.44(-3.98,5.07) |
|  | Sri Lanka | 186428.1(106800.57,256981.35) | 59.09(-13.34,206.7) | 853.04(488.69,1175.88) | 25.36(-31.71,141.68) | 850.52(487.74,1172.85) | 21.87(-33.61,140.37) | 0.69(0.67,0.72) |
|  | China | 7267641.93(6033647.93,8648922.66) | 91.33(58.85,131.09) | 510.96(424.2,608.07) | 59.22(32.2,92.32) | 519.88(428.14,620.97) | 58.2(32.05,90.72) | 1.23(1.14,1.33) |
|  | Georgia | 31040.07(21094.29,40617.35) | -31.05(-56.92,20.32) | 846.99(575.6,1108.32) | 3.64(-35.24,80.85) | 823.76(557.86,1073.06) | 0.88(-37.99,76.41) | 0.37(0.3,0.43) |
|  | Botswana | 25899.14(17675.64,33792.96) | 133.56(45.36,272.26) | 1107.41(755.78,1444.93) | 30(-19.09,107.19) | 1160.91(788.94,1504.59) | 24.52(-22.11,99.48) | 0.68(0.59,0.77) |
|  | Thailand | 480095.6(296209.16,644930.66) | 77.1(-0.99,213.27) | 684.76(422.48,919.86) | 43.67(-19.68,154.13) | 689.95(430.14,916.91) | 42.99(-18.77,148.02) | 1.57(1.54,1.61) |
|  | Uruguay | 25673.9(15848.49,34029.54) | 26.93(-24.68,112.76) | 747.17(461.23,990.34) | 15.97(-31.19,94.39) | 719.95(445.24,960.08) | 12.84(-33.63,90.41) | 0.55(0.48,0.62) |
|  | South Africa | 557220.44(452588.88,675962.76) | 81.18(48.78,124.94) | 1002.4(814.18,1216.01) | 20.04(-1.43,49.03) | 1032.38(843.73,1242.35) | 13.75(-6.03,40.19) | -0.08(-0.18,0.02) |
|  | Saint Lucia | 2126.41(1398.79,2795.03) | 45.87(-13.88,152.18) | 1217.7(801.02,1600.59) | 14.78(-32.24,98.43) | 1172.8(770.09,1535.3) | 2.36(-38.98,73.63) | -0.15(-0.39,0.09) |
|  | Mexico | 1509807.18(1238259.01,1770378.88) | 68.05(43.56,98.13) | 1208.42(991.08,1416.98) | 14.98(-1.77,35.57) | 1216.38(1000.64,1426.15) | 4.35(-9.28,20.99) | -0.01(-0.04,0.02) |
|  | Fiji | 5734.46(3478.69,7922.3) | 28.63(-32.9,129.96) | 629.3(381.75,869.39) | 7.18(-44.09,91.62) | 635.95(384.77,877.83) | 6.95(-43.85,94.42) | 0.14(-0.02,0.31) |
|  | Jamaica | 31310.57(22041.32,40217.99) | 52.37(-3.09,146.26) | 1113.96(784.18,1430.86) | 28.14(-18.5,107.09) | 1105.25(778.29,1423.29) | 24.32(-20.95,100.75) | 0.83(0.76,0.9) |
|  | Indonesia | 1230844.21(981716.98,1526153.22) | 133.1(85.74,200.54) | 474.38(378.36,588.19) | 66.54(32.71,114.73) | 482.5(386.83,598.15) | 77.35(40.04,131.31) | 2.19(2.14,2.25) |
|  | Egypt | 599112.96(393695.78,797251.23) | 117.11(27.02,302.37) | 604.74(397.39,804.74) | 22.06(-28.59,126.22) | 582.19(378.36,776.55) | 27.8(-26.23,141.97) | 1.47(1.35,1.59) |
|  | Paraguay | 56524.57(36105.28,76070.6) | 69.87(0.36,194.28) | 815.6(520.97,1097.63) | -0.84(-41.42,71.78) | 828.46(532.5,1108.82) | -3.79(-43.04,64.15) | -0.37(-0.42,-0.31) |
|  | Saint Vincent and the Grenadines | 1237.81(804.67,1638.25) | 22.24(-28.97,118.23) | 1094.01(711.2,1447.94) | 18.91(-30.91,112.28) | 1068.77(696.25,1417.87) | 10.9(-35.33,93.7) | 0.3(-0.02,0.62) |
|  | Algeria | 258665.17(165192.06,343589.85) | 76.6(-4.52,259.49) | 618.12(394.75,821.06) | 6.69(-42.31,117.19) | 620.86(390.03,828.54) | 15.9(-38.39,139.82) | 0.82(0.79,0.86) |
|  | Suriname | 6790.68(4426.53,9099.46) | 78.15(9.96,225.84) | 1179.17(768.64,1580.07) | 19.59(-26.18,118.74) | 1173.87(768.7,1573.12) | 15.33(-27.87,108.61) | 0.59(0.44,0.74) |
|  | Panama | 45035.59(28487.33,59283.51) | 126.86(32.52,293.47) | 1082.47(684.72,1424.93) | 30.24(-23.92,125.89) | 1081.99(685.26,1423.68) | 25.54(-27.35,113.13) | 0.79(0.72,0.85) |
|  | Turkmenistan | 38727.39(20105.61,54846.05) | 91.75(-8.42,319.69) | 761.89(395.54,1078.99) | 39.77(-33.25,205.93) | 759.14(388.22,1077.71) | 44.79(-32.48,225.1) | 1.46(1.38,1.54) |
|  | Peru | 378914.27(261595.69,500434.84) | 108.73(26.04,283.56) | 1114.6(769.5,1472.07) | 33.42(-19.44,145.17) | 1118.38(775.62,1474.64) | 24.51(-23.48,121.99) | 0.55(0.49,0.61) |
|  | Mauritius | 13569.12(7670.54,18477.3) | 60.6(-16.44,188.22) | 1062.86(600.83,1447.31) | 38.39(-28,148.35) | 1046.39(599.17,1419.71) | 34.52(-28.73,145.78) | 0.94(0.84,1.04) |
|  | Albania | 20479.41(10905.34,28496.17) | -9.25(-57.24,89.42) | 752.82(400.88,1047.52) | 10.42(-47.98,130.47) | 757.92(415.39,1045.52) | 13.51(-45.98,140.38) | 0.79(0.71,0.86) |
|  | Uzbekistan | 261048.57(146822.14,362303.84) | 111.03(5.29,371.51) | 775.15(435.97,1075.82) | 31.27(-34.51,193.29) | 769.33(422.61,1077.09) | 33.79(-34.52,206.56) | 1.11(1.06,1.17) |
|  | Brazil | 2010212.88(1650547.48,2378645.74) | 71.89(41.42,112.64) | 927.8(761.8,1097.85) | 18.08(-2.86,46.07) | 921.97(762.01,1092.35) | 8.23(-9.7,32.76) | 0.45(0.38,0.52) |
|  | Tonga | 568.18(373.74,753.52) | 13.01(-31.27,91.97) | 555.13(365.16,736.21) | 6.87(-35,81.54) | 553.26(361.95,731.11) | 5.39(-35.99,79.41) | 0.11(-0.42,0.64) |
|  | Ecuador | 190530.83(108004.61,266975.47) | 87.7(0.07,271.93) | 1083.28(614.07,1517.91) | 7(-42.96,112.02) | 1101.31(629.96,1534.26) | -1.13(-46.81,92.85) | -0.44(-0.51,-0.37) |
|  | Samoa | 1181.17(753.93,1595.74) | 32.82(-21.55,120.17) | 558.86(356.72,755.01) | 2.83(-39.26,70.46) | 568.81(365.01,761.26) | 1.05(-39.81,65.63) | 0.1(-0.28,0.48) |
|  | Cuba | 63548.07(38183.51,85236.32) | -4.16(-45.23,66.09) | 559.48(336.17,750.42) | -8.6(-47.77,58.39) | 589.94(361.65,791.59) | -2.69(-43.51,68.96) | 0.09(0.05,0.13) |
|  | Equatorial Guinea | 18836.71(10961.99,26476.59) | 500.39(196.37,1116.5) | 1326.68(772.06,1864.76) | 81.97(-10.17,268.71) | 1414.54(835.5,1963.38) | 70.8(-10.44,216.67) | 2.35(2.19,2.51) |
|  | Colombia | 495566.99(302819.87,673451.66) | 72.18(-6.51,243.08) | 1037.26(633.82,1409.58) | 17.3(-36.31,133.72) | 1032.21(632.1,1408.86) | 14.89(-37.07,126.42) | 0.57(0.54,0.61) |
|  | Tunisia | 65877.98(43227.92,86250.4) | 58.11(-6.34,170.72) | 569.31(373.57,745.36) | 15.31(-31.69,97.44) | 587.45(384.36,771.52) | 26.09(-26.16,118.03) | 0.96(0.9,1.01) |
|  | Republic of Moldova | 25511.48(17525.58,33001.2) | -29.41(-53.67,11.29) | 691.71(475.18,894.78) | -14.9(-44.14,34.17) | 676.81(462.38,885.41) | -16.67(-45.8,30.02) | -0.74(-0.82,-0.67) |
| Low middle SDI | Maldives | 3444.46(2169.31,4667.73) | 144.2(36.96,388.91) | 691.08(435.24,936.52) | 8.73(-39.02,117.69) | 717.2(431.66,984.73) | 16.81(-36.29,150.23) | 0.63(0.34,0.93) |
|  | Philippines | 649449.69(539868.04,782123.91) | 146.65(112.02,193.04) | 579.13(481.41,697.44) | 39.2(19.65,65.38) | 580.27(486.4,692.37) | 38.69(20.59,62.57) | 1.19(1.13,1.25) |
|  | Ghana | 304923.43(203529.37,410120.24) | 204.76(81.37,466.57) | 966.9(645.38,1300.47) | 45.12(-13.64,169.78) | 990.78(662.29,1326.41) | 35.89(-17.88,142.77) | 0.99(0.91,1.07) |
|  | Myanmar | 337936.96(208977.64,475311.62) | 95.15(3.11,297.71) | 618.06(382.2,869.31) | 46.71(-22.49,198.98) | 625.43(385.17,880.43) | 44.23(-23.23,187.78) | 1.48(1.4,1.55) |
|  | Sudan | 199031.49(116516.23,282546.72) | 143.71(26.2,418.83) | 487.72(285.52,692.37) | 20.63(-37.53,156.8) | 455.7(261.97,653.28) | 25.54(-36.52,175.87) | 1.21(1.16,1.27) |
|  | Zambia | 172615.43(102131.62,252741.72) | 185.23(42.8,580.76) | 946.48(560,1385.82) | 24.23(-37.81,196.49) | 964.53(570.78,1405.5) | 12.83(-42.56,143.6) | 0.17(0.09,0.26) |
|  | Democratic People's Republic of Korea | 96878.94(62061.65,129410.95) | 6.04(-36.88,86.01) | 369.3(236.58,493.32) | -14.89(-49.34,49.29) | 377.62(240.31,503.12) | -13.84(-48.45,51.35) | -0.4(-0.45,-0.36) |
|  | Dominican Republic | 114610.24(79666.25,150414.48) | 132.28(48.33,290.17) | 1053.22(732.1,1382.25) | 53.76(-1.81,158.27) | 1059.4(733.65,1384.44) | 49.74(-3.61,148.25) | 1.48(1.42,1.54) |
|  | Guatemala | 156522.42(93007.74,222703.06) | 131.95(26.56,326.22) | 880.5(523.21,1252.8) | 3.95(-43.28,91.01) | 919.78(558,1283.3) | -2.41(-44.91,72.85) | -0.06(-0.12,0) |
|  | El Salvador | 51796.33(30183.4,72704.37) | 35.37(-28.6,152.8) | 827.93(482.46,1162.13) | 13.98(-39.88,112.85) | 827.1(486.04,1155.58) | 8.98(-41.96,98.61) | 0.22(0.16,0.27) |
|  | Honduras | 97269.99(60277.35,133916.14) | 115.3(20.31,299.36) | 991.1(614.17,1364.49) | 3.29(-42.28,91.6) | 1041.93(658.57,1416.6) | -2(-44.53,73.08) | -0.11(-0.15,-0.06) |
|  | Timor-Leste | 7417.53(4827.44,10133.78) | 124.25(30.01,310.17) | 555.69(361.65,759.19) | 31.54(-23.74,140.59) | 546.6(356.8,744.61) | 23.96(-28.04,120.05) | 1.1(0.9,1.29) |
|  | Guyana | 8110.34(5326.52,10870.57) | 15.44(-30.69,96.06) | 1052.33(691.12,1410.47) | 15.34(-30.74,95.9) | 1059.66(697.51,1410.89) | 11.1(-32.59,87.42) | 0.2(0.08,0.33) |
|  | Namibia | 23624.38(16066.22,31273.44) | 108.59(32.81,238.64) | 983.07(668.56,1301.37) | 22.36(-22.09,98.65) | 1033.71(707.42,1357.94) | 16.52(-26.17,88.49) | 0.38(0.29,0.47) |
|  | Kyrgyzstan | 46996.43(27373.05,64185.32) | 57.12(-18.66,220.74) | 719.1(418.84,982.11) | 7.28(-44.46,118.99) | 710.27(407.95,976.48) | 8.2(-45.36,121.62) | 0.32(0.26,0.39) |
|  | Tajikistan | 71449.86(38821.71,103379.89) | 100.14(-7.34,359.47) | 752.7(408.98,1089.08) | 13.34(-47.53,160.2) | 747.83(391.61,1084.65) | 14.46(-48.96,177) | 0.66(0.6,0.72) |
|  | India | 10089995.36(8394949.41,11860538.57) | 132.37(95.9,181.16) | 725.53(603.65,852.84) | 42.96(20.52,72.97) | 738.02(617.7,859.68) | 36.29(16.82,61.93) | 1.19(1.1,1.29) |
|  | Tuvalu | 56.94(34.35,78.31) | 51.1(-20.84,186.38) | 482.64(291.18,663.8) | 19.7(-37.3,126.86) | 481.46(291.34,659.79) | 18.89(-37.89,125.21) | 0.7(-1.03,2.45) |
|  | Nicaragua | 49625.89(29408.62,70018.72) | 60.1(-14.78,206.82) | 762.26(451.72,1075.5) | -4.41(-49.12,83.19) | 783.72(473.82,1087.51) | -8.47(-50.2,67.98) | -0.39(-0.45,-0.33) |
|  | Viet Nam | 554070.17(354196.1,743041.73) | 77.22(3.67,194.87) | 574.92(367.53,771.01) | 24.94(-26.91,107.88) | 592.6(375.59,800.61) | 32.67(-22.44,118.04) | 1.21(1.17,1.25) |
|  | Kenya | 477962.37(399228.22,568078.05) | 186.19(145.46,245.45) | 951.59(794.84,1131.01) | 32.15(13.35,59.52) | 949.23(797.12,1118.72) | 24.1(7.69,48.13) | 0.79(0.69,0.89) |
|  | Kiribati | 605.84(353.23,869.83) | 65.38(-16.55,267.67) | 510.73(297.78,733.28) | 3.26(-47.89,129.56) | 512.73(295.32,735.77) | 0.23(-49.03,121.07) | -0.04(-0.62,0.55) |
|  | Nigeria | 1648424.47(1300996.76,2029324.15) | 175.6(128.29,243.97) | 767.34(605.61,944.65) | 15.7(-4.16,44.41) | 794.95(635.27,962.2) | 10.9(-7.5,36.87) | 0.37(0.28,0.46) |
|  | Lesotho | 16481.91(10428.08,22406.64) | 57.2(-10.48,180.2) | 788.01(498.57,1071.27) | 35.83(-22.65,142.11) | 827.9(537.57,1118.97) | 30.67(-24.13,126.36) | 1.12(1.01,1.23) |
|  | Palestine | 30978.64(20051.63,42181) | 171.23(49.74,397.92) | 625(404.54,851.01) | 13.27(-37.46,107.95) | 597.18(382.75,811.91) | 18.07(-36.11,124.45) | 0.63(0.51,0.75) |
|  | Morocco | 219158.39(139187.26,301641.13) | 69.82(-5.73,208.49) | 609.58(387.15,839.01) | 19.49(-33.67,117.06) | 614.24(386.02,851.25) | 29.52(-29.21,138.79) | 1.24(1.21,1.27) |
|  | Mauritania | 40204.76(25710.81,55188.24) | 196.71(57.83,542.27) | 1001.55(640.48,1374.8) | 52.73(-18.76,230.62) | 959.59(597.77,1326.19) | 51.69(-23.16,256.67) | 1.37(1.27,1.47) |
|  | Marshall Islands | 279.79(169.19,394.79) | 31.22(-30.78,146) | 492.22(297.65,694.54) | 5.56(-44.31,97.91) | 494.42(297.3,694.63) | 5.2(-44.62,101.91) | 0.05(-0.82,0.92) |
|  | Venezuela (Bolivarian Republic of) | 339305.59(227769.02,445956.11) | 65.38(-0.28,199.56) | 1208.83(811.46,1588.79) | 10.95(-33.1,100.96) | 1208.09(810.7,1595.87) | 3.95(-36.43,82.85) | 0.09(0.06,0.12) |
|  | Mongolia | 28147.04(17599.71,37563.37) | 124.04(26.79,341.75) | 830.89(519.53,1108.85) | 42.43(-19.39,180.85) | 855.81(537.79,1139.25) | 38.73(-20.13,160.57) | 1.19(1.11,1.28) |
|  | Eswatini | 10441.92(6539.39,14050.82) | 85.74(6.08,225.3) | 914.27(572.57,1230.25) | 31.19(-25.07,129.77) | 980.21(619.12,1299.9) | 19.16(-30.51,97.5) | 0.45(0.31,0.58) |
|  | Micronesia (Federated States of) | 513.23(306.8,709.39) | -1.04(-45.62,89.22) | 502.6(300.44,694.69) | 1.06(-44.47,93.24) | 505.35(300.08,689.99) | 0.41(-45.76,91.81) | -0.06(-0.62,0.49) |
|  | Lao People's Democratic Republic | 38561.3(24570.54,53468.24) | 125.56(17.73,321.24) | 538.7(343.25,746.95) | 30.82(-31.72,144.31) | 548.98(351.05,758.87) | 26.57(-31.96,131.47) | 0.77(0.7,0.85) |
|  | Sao Tome and Principe | 2338.11(1598.61,3088.69) | 144.63(44.38,349.44) | 1138.4(778.35,1503.85) | 44.75(-14.57,165.95) | 1161.87(804.94,1514.13) | 37.73(-16.56,138.25) | 1.11(0.77,1.46) |
|  | Bolivia (Plurinational State of) | 97356.75(60821.12,130518.61) | 96.75(7.01,310.78) | 810.51(506.35,1086.59) | 5.17(-42.8,119.59) | 834.41(518.46,1122.02) | -2.11(-45.47,92.86) | 0.08(0.01,0.15) |
|  | Cameroon | 255760.84(150918.32,367054.96) | 212.39(55.34,549.95) | 878.85(518.59,1261.28) | 11.54(-44.54,132.07) | 932.99(556.56,1316.42) | 4.21(-47,101.35) | 0.03(-0.07,0.12) |
|  | Congo | 61579.06(38392.12,84478.68) | 151.67(28.85,423.09) | 1169.4(729.08,1604.28) | 16.85(-40.18,142.86) | 1225.17(769.65,1661.91) | 8.85(-42.79,111.23) | 0.1(0.02,0.18) |
|  | Syrian Arab Republic | 76408.67(48439.96,104440.57) | 14.68(-36.66,105.49) | 527.27(334.27,720.71) | 2.04(-43.64,82.85) | 523.09(327.51,707.97) | 13.11(-38.04,106.39) | 0.39(0.33,0.44) |
|  | Belize | 3876.92(2587.8,5068.55) | 188.01(67.18,381.55) | 945.38(631.03,1235.95) | 30.55(-24.22,118.27) | 958.1(638.39,1240.3) | 25.19(-25.99,106.93) | 0.54(0.29,0.8) |
|  | Cabo Verde | 8002.36(5470.8,10514.53) | 121.68(34.24,287.04) | 1419.96(970.75,1865.72) | 38.31(-16.25,141.47) | 1449.08(991.48,1889.04) | 30.7(-20.67,119.08) | 0.9(0.74,1.06) |
|  | Nauru | 58.66(33.4,81.73) | 0.36(-47.26,101.73) | 555.99(316.53,774.6) | -2.46(-48.74,96.07) | 555.39(309.7,772.9) | -6.4(-51.8,92.18) | -0.41(-2.3,1.52) |
| Low SDI | Vanuatu | 1419.74(854.5,1977.03) | 97.92(7.47,249.42) | 482(290.1,671.2) | 1.72(-44.76,79.58) | 478.65(287.7,663.87) | -1.62(-46.57,73.46) | -0.19(-0.61,0.23) |
|  | Yemen | 179797.25(120511.21,245256.72) | 160.13(48.18,377.11) | 570.73(382.54,778.52) | 13.37(-35.42,107.93) | 516.37(339.08,703.51) | 19.97(-33.66,132.13) | 1.19(1.08,1.3) |
|  | Uganda | 366963.44(209038.39,538758.52) | 211.02(44.95,607.22) | 892.47(508.39,1310.28) | 30.97(-38.96,197.82) | 894.47(522.02,1301.21) | 19.34(-42.17,154.39) | 0.32(0.2,0.44) |
|  | Gambia | 17747.74(11538.25,24331.19) | 239.23(85.24,554.93) | 790.24(513.76,1083.38) | 49.83(-18.18,189.27) | 748.02(473.55,1033.87) | 48.36(-21.94,204.58) | 1.61(1.44,1.78) |
|  | Afghanistan | 199205.25(121137.28,290200.76) | 269.15(92.12,664.55) | 520.42(316.47,758.15) | 10.12(-42.69,128.08) | 466.17(271.48,693.22) | 7.35(-46.01,134.09) | 0.41(0.33,0.5) |
|  | Djibouti | 10426.35(6425.08,14577.47) | 234.13(70.25,710.17) | 866.84(534.18,1211.96) | 35.01(-31.21,227.36) | 853.22(502.69,1203.57) | 37.94(-32.56,257.09) | 1.11(0.89,1.33) |
|  | Guinea | 80916.36(43100.62,122784.11) | 160.56(17.76,552.18) | 640(340.9,971.15) | 27.49(-42.38,219.12) | 625.13(320.42,959.1) | 26.74(-44.88,229.39) | 0.76(0.68,0.85) |
|  | Burundi | 86391.15(52353.68,126594.12) | 130.95(16.2,416.04) | 723.89(438.68,1060.75) | 7.81(-45.76,140.89) | 735.36(448.08,1057.09) | -1.08(-48.54,110.82) | -1.2(-1.32,-1.08) |
|  | Bangladesh | 977717.4(653236.88,1350396.69) | 64.87(1.26,189.28) | 613.91(410.17,847.92) | 12.9(-30.66,98.08) | 607.7(407.8,837.76) | 20.3(-29.41,120.96) | 1.03(0.92,1.13) |
|  | Bhutan | 6697(4567.84,8792.3) | 42.88(-14.02,149.52) | 887.9(605.61,1165.7) | 15.96(-30.22,102.52) | 902.53(612.36,1180.68) | 11.45(-32.49,85.14) | 0.41(0.24,0.57) |
|  | Eritrea | 58691.34(37895.91,83684.08) | 234.94(74.3,604.38) | 874.53(564.67,1246.93) | 49.79(-22.05,215.02) | 866.22(557.61,1227.05) | 38.56(-28.93,183.55) | 0.82(0.7,0.94) |
|  | Cambodia | 92147.85(60436.37,126161) | 121.7(28.78,301.3) | 555(364.01,759.86) | 38.54(-19.52,150.78) | 557.6(364.04,764.75) | 39.11(-20.2,149.28) | 1.38(1.32,1.43) |
|  | Comoros | 6459.07(4110.64,8986.87) | 109.51(6.52,385.47) | 904.19(575.44,1258.05) | 36.6(-30.55,216.52) | 878.53(548.74,1223.13) | 40.01(-31.25,249.58) | 1.29(1.07,1.51) |
|  | Guinea-Bissau | 15222.48(9305.6,22394.02) | 147.26(19.62,465.57) | 800.68(489.46,1177.89) | 31.01(-36.62,199.66) | 791.56(472.48,1153.91) | 20.38(-41.48,163.12) | 0.38(0.23,0.52) |
|  | Liberia | 39240.7(23870.69,57730.93) | 158.49(33.18,425.57) | 819.24(498.35,1205.26) | 6(-45.39,115.52) | 866.5(546.43,1250.75) | 1.98(-44.02,92.16) | 0.35(0.22,0.48) |
|  | Madagascar | 191056.25(117767.2,274520.22) | 146.23(27.29,425.17) | 715.83(441.24,1028.54) | 10.26(-43,135.16) | 722.24(442.39,1029.02) | 4.44(-45.05,109.45) | 0.05(-0.04,0.15) |
|  | Mali | 115014.22(59295.17,178980.9) | 247.94(47.68,692.16) | 524.76(270.54,816.61) | 37.68(-41.56,213.46) | 494.59(241.52,779.82) | 35.06(-45.53,214.73) | 0.51(0.39,0.62) |
|  | Haiti | 85597.43(53972,116150.67) | 82.71(-2.03,233.96) | 690.19(435.18,936.54) | -6.36(-49.79,71.14) | 718.39(460.23,970.22) | -8.89(-50.28,63.21) | -0.41(-0.46,-0.37) |
|  | Pakistan | 1624208.96(1152391.64,2110430.81) | 184.15(79.54,382.68) | 724.89(514.32,941.89) | 43.09(-9.58,143.07) | 696.71(490.6,916.99) | 38.96(-15.92,144.07) | 1.26(1.16,1.36) |
|  | Ethiopia | 772869.64(586948.53,984474.59) | 263.74(155.85,477.59) | 718.34(545.54,915.01) | 73.74(22.21,175.89) | 703.02(535.5,885.93) | 53.97(11.36,135.94) | 1.13(1,1.27) |
|  | Zimbabwe | 120902.31(80568.22,162682.6) | 64.11(-1.26,173.68) | 805.43(536.73,1083.77) | 13.03(-31.99,88.49) | 896.86(605.19,1181.95) | 4(-36.16,68.23) | -0.07(-0.18,0.04) |
|  | Mozambique | 244775.18(140789,371106.22) | 227.9(61.11,658.38) | 828.96(476.8,1256.79) | 45.15(-28.68,235.71) | 849.26(498.35,1262.51) | 36.7(-29.67,198.14) | 1.03(0.84,1.23) |
|  | Angola | 317381.8(188794.89,453404.83) | 264.53(85.12,663.68) | 1053.08(626.42,1504.4) | 24.8(-36.62,161.46) | 1151.32(703.35,1617.45) | 16.93(-37.43,125.08) | 0.73(0.66,0.81) |
|  | South Sudan | 63575.69(31917.06,102364.14) | 80.1(-20.94,395.4) | 684.86(343.82,1102.71) | 13.68(-50.09,212.7) | 684.65(339.02,1116.96) | 10.56(-52.57,208.74) | 0.48(0.38,0.57) |
|  | Nepal | 294598.89(205199.06,394859.16) | 76.03(7.96,196.71) | 968.55(674.63,1298.18) | 13.06(-30.66,90.58) | 987.84(689.67,1313.83) | 7.18(-33.73,76.73) | 0.64(0.59,0.7) |
|  | Burkina Faso | 136771.84(72011.77,216343.22) | 159.24(18.61,497.84) | 602.74(317.35,953.4) | 9.19(-50.04,151.81) | 598.57(312.1,944.54) | 3.03(-52.94,138.72) | 0.14(0.07,0.22) |
|  | Niger | 124162.5(68525.22,195614.13) | 216.7(37.63,687.69) | 532.99(294.16,839.71) | 9.07(-52.6,171.28) | 495.13(254.68,785.83) | 7.17(-54.93,182.54) | 0.3(0.21,0.38) |
|  | Democratic Republic of the Congo | 717674.63(424003.07,1027676.78) | 173.58(44.99,447.78) | 818.6(483.63,1172.2) | 20.42(-36.18,141.12) | 864.88(535.81,1207) | 11.91(-37.52,106.89) | 0.45(0.34,0.56) |
|  | Malawi | 137350.85(84343.01,200107.18) | 167.98(41.55,454.97) | 744.76(457.34,1085.05) | 38.85(-26.66,187.55) | 736.98(456.63,1047.57) | 24.29(-32.03,143.59) | 0.63(0.49,0.76) |
|  | Papua New Guinea | 44059.5(25942.42,65239.17) | 160.32(34.22,397.04) | 446.55(262.93,661.21) | 7.85(-44.39,105.91) | 454.93(268.54,662.64) | 4.01(-45.34,94.1) | 0.02(-0.06,0.1) |
|  | Central African Republic | 39517.23(22898.26,58591.72) | 87.82(-4.3,274.96) | 745.63(432.05,1105.53) | -2.75(-50.45,94.14) | 808.05(485.25,1158.87) | -8.04(-50.41,71.59) | -0.42(-0.52,-0.33) |
|  | Benin | 103235.63(60843.66,148652.73) | 167.51(31.06,458.34) | 815.08(480.38,1173.66) | 2.49(-49.79,113.92) | 887.5(555.69,1258.32) | -5.53(-50.61,80.36) | -0.63(-0.78,-0.48) |
|  | Rwanda | 98030.35(61348.27,137391.32) | 123.7(19.4,388.36) | 772.62(483.51,1082.83) | 26.46(-32.5,176.08) | 783.87(490.46,1091.36) | 15.97(-36.53,140.67) | 0.48(0.37,0.6) |
|  | Senegal | 135396.53(85463.91,189935.69) | 204.75(56.11,584.78) | 894.65(564.71,1255.02) | 53.46(-21.39,244.82) | 858.79(526.13,1208.92) | 56.07(-22.85,273.63) | 1.43(1.25,1.6) |
|  | Solomon Islands | 3097.69(1883.37,4512.44) | 101.51(6.79,286.59) | 472.47(287.26,688.26) | 4.64(-44.55,100.74) | 469.98(282.71,687.5) | 0.97(-46.65,96.3) | -0.18(-0.47,0.11) |
|  | Togo | 69774.2(45243.85,96349.01) | 103(12.72,325.19) | 880.82(571.15,1216.29) | -6.13(-47.88,96.61) | 926.41(607.33,1255.81) | -12.01(-49.1,66.68) | -0.64(-0.74,-0.54) |
|  | Côte d'Ivoire | 220349.44(133000.66,312834.74) | 161.79(38.25,440.35) | 841.94(508.19,1195.32) | 22.3(-35.41,152.43) | 904.86(563.88,1256.26) | 12.57(-38.15,115.48) | 0.16(0.04,0.28) |
|  | Sierra Leone | 47800.92(23905.55,74519.98) | 156.11(7.07,593.35) | 576.97(288.55,899.48) | 12.9(-52.8,205.64) | 564.94(280.2,878.66) | 12.14(-53.93,206.62) | 0.32(0.23,0.4) |
|  | United Republic of Tanzania | 434716.21(253412.62,622226.65) | 222.75(56.6,651.13) | 766.21(446.65,1096.7) | 47.34(-28.51,242.91) | 754.13(432.33,1085.03) | 36.36(-34.45,217.27) | 0.59(0.49,0.69) |
|  | Somalia | 99487.11(53087.8,154381.77) | 265.67(61.92,825.3) | 489.05(260.96,758.89) | 28.48(-43.11,225.1) | 448.56(228.54,698.95) | 22.69(-48.86,229.67) | 0.57(0.46,0.68) |
|  | Chad | 96175.23(46398.77,152710.69) | 232.17(44.58,722.66) | 586.48(282.94,931.23) | 21.98(-46.91,202.1) | 587.44(285.86,939.74) | 19.39(-47.61,188.11) | 0.7(0.63,0.78) |
